# Identification of novel soybean microRNAs involved in abiotic and biotic stresses

**DOI:** 10.1186/1471-2164-12-307

**Published:** 2011-06-10

**Authors:** Franceli R Kulcheski, Luiz FV de Oliveira, Lorrayne G Molina, Maurício P Almerão, Fabiana A Rodrigues, Juliana Marcolino, Joice F Barbosa, Renata Stolf-Moreira, Alexandre L Nepomuceno, Francismar C Marcelino-Guimarães, Ricardo V Abdelnoor, Leandro C Nascimento, Marcelo F Carazzolle, Gonçalo AG Pereira, Rogério Margis

**Affiliations:** 1 Centre of Biotechnology and PPGBCM, Laboratory of Genomes and Plant Population, building 43431, Federal University of Rio Grande do Sul - UFRGS, P.O. Box 15005, CEP 91501-970, Porto Alegre, RS, Brazil; 2 PPGGBM at Federal University of Rio Grande do Sul - UFRGS, Porto Alegre, RS, Brazil; 3 EMBRAPA Soja, Rodovia Carlos João Strass, Distrito de Warta, CEP 86001-970, Londrina, PR, Brazil; 4 Institute of Biology, Laboratory of Genomic and Expression, State University of Campinas, CEP 13083-970, Campinas, SP, Brazil; 5 National Center for High Performance Processing (CENAPAD-SP), State University of Campinas, CEP 13083-970, Campinas, SP, Brazil

## Abstract

**Background:**

Small RNAs (19-24 nt) are key regulators of gene expression that guide both transcriptional and post-transcriptional silencing mechanisms in eukaryotes. Current studies have demonstrated that microRNAs (miRNAs) act in several plant pathways associated with tissue proliferation, differentiation, and development and in response to abiotic and biotic stresses. In order to identify new miRNAs in soybean and to verify those that are possibly water deficit and rust-stress regulated, eight libraries of small RNAs were constructed and submitted to Solexa sequencing.

**Results:**

The libraries were developed from drought-sensitive and tolerant seedlings and rust-susceptible and resistant soybeans with or without stressors. Sequencing the library and subsequent analyses detected 256 miRNAs. From this total, we identified 24 families of novel miRNAs that had not been reported before, six families of conserved miRNAs that exist in other plants species, and 22 families previously reported in soybean. We also observed the presence of several isomiRNAs during our analyses. To validate novel miRNAs, we performed RT-qPCR across the eight different libraries. Among the 11 miRNAs analyzed, all showed different expression profiles during biotic and abiotic stresses to soybean. The majority of miRNAs were up-regulated during water deficit stress in the sensitive plants. However, for the tolerant genotype, most of the miRNAs were down regulated. The pattern of miRNAs expression was also different for the distinct genotypes submitted to the pathogen stress. Most miRNAs were down regulated during the fungus infection in the susceptible genotype; however, in the resistant genotype, most miRNAs did not vary during rust attack. A prediction of the putative targets was carried out for conserved and novel miRNAs families.

**Conclusions:**

Validation of our results with quantitative RT-qPCR revealed that Solexa sequencing is a powerful tool for miRNA discovery. The identification of differentially expressed plant miRNAs provides molecular evidence for the possible involvement of miRNAs in the process of water deficit- and rust-stress responses.

## Background

Small, non-coding RNAs have been characterized in plants as important factors involved in gene expression regulation in developmental processes [1,2], as well as adaption to biotic and abiotic stress conditions [3,4]. In general, small RNAs are grouped into two major classes: microRNAs (miRNAs) and short-interfering RNAs (siRNAs). These two classes of small RNAs cannot be discriminated by either their chemical composition or mechanism of action [5,6]. However, siRNAs and miRNAs can be distinguished by their origin, evolutionary conservation and the types of genes that they silence [5,6]. In this way, miRNAs are well differentiated due to some particular characteristics. These characteristics include the following: derived from genomic loci distinct from other recognized genes, processed from transcripts that can form local RNA hairpin structures, and usually, miRNAs sequences are nearly always conserved in related organisms [6,7].

In plants, *MIRNA *genes are transcribed by RNA polymerase II enzymes (Pol II) generating primary miRNA (pri-miRNA). The pri-miRNA forms an imperfect fold-back structure, which is processed into a stem-loop precursor (pre-miRNA) by nuclear RNaseIII-like enzymes called DICER-LIKE proteins (e.g., DCL1) [8]. The resulting pre-miRNA contains a miRNA:miRNA* intermediate duplex, formed by a self-complementary fold-back structure. A mature miRNA sequence can range from 19 to 24 nucleotides (nt) in length and act as a regulatory molecule in post-transcriptional gene silencing by base pairing with target mRNAs. This leads to mRNA cleavage or translational repression, depending on the degree of complementarity between the miRNA and its target transcript [6,9]. The same mature miRNA can also present several variants of their sequence in length. These populations of miRNA variants are called isomiRNAs, which are isoforms of microRNAs [10]. They are caused by an imprecise or alternative cleavage of Dicer during pre-miRNA processing [10]. IsomiRNAs have been recently identified in both plants and animals [10-12].

The first plant miRNAs were described in *Arabidopsis thaliana *[13,14] and later in other species. Currently, miRNAs have been reported in 41 plants species, and all of their sequences have been deposited in a publicly-available miRNA database, miRBase (http://www.sanger.ac.uk/cgi-bin/Rfam/mirna/browse.pl) [15-18]. Several miRNAs have been identified in plants, and they are characterized in a wide variety of metabolic and biological processes in plants with important functions in development [19,20], phytohormone signaling [21], flowering and sex determination [22] and responses to biotic and abiotic stresses [3,4,19,23-25].

In soybean (*Glycine max *(L.) Merrill), the major legume crop worldwide, Subramanian et al. in 2008 [26] identified 35 novel miRNA families for the first time. In this study, the role of miRNAs in soybean-rhizobial symbiosis was investigated [26]. During that same year, Zhang et al. [27] used a comparative genome-based *in silico *screening of soybean EST databases and quantitative PCR to provide evidence for 69 miRNAs belonging to 33 families. A second study involving miRNAs and soybean root nodules was performed by Wang and colleagues [28]. They identified 32 miRNAs belonging to 11 miRNA families. The identification of nine novel miRNAs in wild soybean (*Glycine soja*) was also reported by Chen et al. [29]. Another study looked at four different soybean tissues (root, seed, flower and nodule) and identified 87 novel soybean miRNAs [30]. Recently, Song and coworkers [31] identified 26 new miRNAs and their related target genes from developing soybean seeds. Although these studies resulted in a large number of miRNAs identified in soybean, none of them looked at microRNAs with respect to biotic and abiotic stresses.

Drought is the major abiotic stress factor to negatively affect soybean productivity around the world. The impact of limited water during the flower formation can cause shorter flowering periods [32,33], and water stress during the later phases of soybean reproductive development has been reported to accelerate senescence, which decreases the duration of the seed-filling period [32,33]. With regards to biotic stress, Asian soybean rust (ASR) is a foliar disease caused by the fungus *Phakopsora pachyrhizi *Sydow & Sydow. This pathogen presents a rapid aerial spread and a high capacity to colonize leaf tissue and, to a lesser extent, stem and pods [34]. ASR is one of the most severe diseases on the soybean culture, which causes damage between 10% and 90% in the different regions where it has been identified [35,36]. This disease is the main threat in soybean-producing countries.

Currently, there are 203 miRNAs identified in *Glycine max *(miRBase database, release 16, http://www.mirbase.org/); however, none of these miRNAs were associated with water deficit or ASR stress conditions. We consider that the identification of these miRNAs is important to understanding small RNA-mediated gene regulation in soybean roots under water deficit stress and in leaves during rust infection. In this context, our goal was to identify new miRNAs and to discover those that may be regulated by water deficit and soybean rust stress. Using high-throughput sequencing, we constructed four libraries of small RNAs from the roots of drought-sensitive and tolerant seedlings in response to control or water deficit conditions. We also constructed four libraries from leaves of rust-susceptible and resistant seedlings with mock and infected conditions. A set of eight small RNAs libraries was analyzed from soybean plants. A total of 256 miRNAs were detected in Solexa sequencing. We discovered 24 novel miRNAs families and also detected several isomiRNAs in soybean. In our RT-qPCR analysis, we verified that the expression profile of several miRNAs varied during abiotic and biotic stresses. This study has important implications for gene regulation under water deficit and pathogen-infection conditions and also contributes significantly to increase the number of identified miRNAs in soybean.

## Methods

### Plant materials and treatments

#### Water deficit assay

For water deficit treatment, we used the soybean (*Glycine max *(L.) Merrill) cultivars 'Embrapa 48' as a drought-tolerant standard and 'BR 16' as a sensitive standard [37]. Plants were grown in a greenhouse at Embrapa-Soybean (Londrina, Brazil) using a hydroponic system compound for plastic containers (30 liters) and an aerated pH 6.6-balanced nutrient solution. Seeds were pre-germinated on moist filter paper in the dark at 25°C ± 1°C and in 65% ± 5% relative humidity. Plantlets were then placed in polystyrene supports so the roots of the seedlings were completely immersed in the nutrient solution. Each seedling tray was maintained in a greenhouse at 25°C ± 2°C and in 60% ± 5% relative humidity under natural daylight (photosynthetic photon flux density (PPFD) = 1.5 × 10^3 ^μmoles m^-2 ^s^-1^, equivalent to 8.93 × 10^4 ^lux) for a 12 h day. After 15 days, seedlings with the first trifoliate leaf fully developed (V2 developmental stage) [38] were submitted to different water-deficit treatments according to Martins et al. [39]. The nutrient solution was removed from each plastic container where the roots were kept in the tray in the dark without nutrient solution or water for 0 minutes (T0 or control), 125 minutes (T125) and 150 minutes (T150). At the end of each water-deficit period, the roots of the seedlings were immediately frozen in liquid nitrogen and stored at -80°C until RNA extraction. The experimental design was a factorial (cultivars × duration of water deficit) with three replicates. Each replicate was composed of five plantlets that were sampled in bulk. Four libraries of small RNAs were constructed for the water deficit-stress assays from the following root tissues: 1) roots of drought-sensitive seedlings submitted to 0 minutes of stress (Drought-Sensitive Root Control (DSRC)); 2) roots of drought-sensitive seedlings submitted to 125 minutes and 150 minutes of stress (Drought-Sensitive Root Treated (DSRT)); 3) roots from drought-tolerant seedlings submitted to 0 minutes of stress (Drought-Tolerant Root Control (DTRC)); and 4) roots of drought-tolerant seedlings submitted to 125 minutes and 150 minutes of stress (Drought-Tolerant Root Treated (DTRT)).

#### Asian Soybean Rust assay

The ASR reaction was evaluated in soybean plants in a greenhouse at Embrapa-Soybean (Londrina, Brazil) using a field population of *Phakopsora pachyrhizi *collected from soybean fields in the state of Mato Grosso, which were maintained for over 10 generations on the susceptible cv. BRSMS-Bacuri. ASR identification was confirmed by ITS-sequencing analysis as described by Silva et al. [40], and it revealed a similarity to the MUT Zimbabwe isolate. The soybean plants were grown in a pot-based system. The 'Embrapa 48' genotype was used as a susceptible host plant, which develops a susceptible lesion (TAN) after *Phakopsora pachyrhizi *infection. The 'PI561356' genotype was used as the resistant host, which carries an ASR resistance gene mapped onto linkage group G (Ricardo V. Abdelnoor, personal communication) and develops a reddish-brown (RB) lesion with few or no spores.

Urediniospores were collected from infected BRSMS-Bacuri plants in a separate greenhouse by tapping infected leaves over a plastic tray. The urediniospores were then diluted in distilled water with 0.05% Tween-20 to a final concentration of 3 × 10^5 ^spores/mL. This spore suspension was sprayed onto three plants per pot at the V2 to V3 growth stages [38]. A solution without the spores was used for the mock inoculations. Following the ASR or mock inoculations, water-misting bags were placed over all plantlets for one day to aid the infection process and to prevent cross-contamination of the mock-infected plants. The third trifoliolate leaves of six plants were collected 12 hours after inoculation (hai) for RNA extraction. The experiment followed a completely randomized design with the three replicates as blocks and a full factorial treatment structure consisting of three treatment factors: hai (12 hours), genotype (resistant or susceptible), and inoculation type (ASR or mock).

For the rust-stress assay, we constructed the other four libraries of small RNAs from leaves which were compounded by: 1) leaves of rust-susceptible seedlings with mock inoculation (Rust-Susceptible Leaf Control (RSLC)); 2) leaves of rust-susceptible seedlings with rust-spore inoculation (Rust-Susceptible Leaf Treated (RSLT)); 3) leaves of rust-resistant seedlings with mock inoculation (Rust-Resistant Leaf Control (RRLC)); and 4) leaves of rust-resistant seedlings with rust-spore inoculation (Rust-Resistant Leaf Treated (RRLT)).

### RNA extraction and sequencing

Total RNA was isolated from fresh leaves and root materials using Trizol (Invitrogen, CA, USA), and the RNA quality was evaluated by electrophoresis on a 1% agarose gel. The amount of the RNA was verified using a Quibit fluorometer and Quant-iT RNA assay kit according to the manufacturer's instructions (Invitrogen, CA, USA). Total RNA ( > 10 μg) was sent to Fasteris Life Sciences SA (Plan-les-Ouates, Switzerland) for processing and sequencing using Solexa technology on the Illumina Genome Analyzer GAII. The libraries were constructed from the eight bar-coded samples (DSRC, DSRT, DTRC, DTRT, RSLC, RSLI, RRLC and RRLI) sequenced in a total of two channels. Quality scores were generated from Illumina's data analysis pipeline, which are similar to SAGE Phred scores with a maximum value of 40. Quality scores are based on the relative confidence of base calls using elements of cluster generation and image quality. Briefly, the processing by Illumina for the miRNA analyses consisted of the following successive steps: acrylamide gel purification of the RNA bands corresponding to the size range 20-30 nt, ligation of the 3' and 5' adapters to the RNA in two separate subsequent steps each followed by acrylamide gel purification, (3) cDNA synthesis followed by acrylamide gel purification, and a final step of PCR amplification to generate cDNA colonies template library for Illumina sequencing. After removing the adapter sequences, the sequences were trimmed into different read lengths from 19 to 24 nt for further analysis.

### Prediction of miRNAs

The reads were grouped into unique sequences, and the read counts were calculated for each library. The sequences that presented low read counts (read count < = 2) were discarded from the final list of unique sequences, which are referred to as a tag. The sequences were mapped into the soybean genome (http://www.phytozome.net) assembly using the SOAP program [41], which returns information concerning the alignment position, chromosome number and strand. No mismatches were allowed in the alignments. The tag alignment position's upstream and downstream genomic sequences (200 bp each) were extracted from the genome assembly using homemade Perl scripts. These genomic regions were then aligned against the reverse complement of its respective tag (rc-tag) using the Smith-Waterman algorithm [42]. To ensure that these pre-miRNA sequences could be precisely processed into mature miRNA, the candidates were examined according the following criteria [43]: i) the miRNA and anti-sense miRNA should derive from the opposite stem-arms and must be entirely within the arm of the hairpin; ii) base-pairing between the miRNA and anti-sense miRNA were restricted to four or fewer mismatches; and iii) the frequency of asymmetric bulges was restricted to less than one and the size should be less than two bases. The genomic regions that were not possible to align the tag and rc-tag were discarded. Finally, the genomic regions that were limited between the alignment positions of the tag and rc-tag were considered as pre-microRNA candidates. From all the pre-microRNA candidate sequences that we selected, only the ones with no more than five matches to the soybean genome were selected for analyzing the secondary structure using the RNA-folding program Mfold [44]. If a perfect stem-loop structure was formed, the small RNA sequence was at one arm of the stem, and the respective anti-sense sequence was at the opposite arm; then, the small RNA was consisted as a novel soybean miRNA.

### miRNA validation and expression analysis by RT-qPCR

To validate predicted new miRNAs, RT-qPCR in respect to eleven miRNAs was performed to examine their expression across the eight different libraries. From those, six were new miRNAs belonging to conserved soybean miRNAs families (MIR166a-5p, MIR166f, MIR169f-3p, MIR482bd-3p, MIR1513c, MIR4415b); one new miRNA pertencing to conserved miRNAs families in other plants species (MIR397ab); and four were miRNAs belonging to novel miRNAs families (MIR-Seq07, MIR-Seq11, MIR-Seq13, MIR-Seq15ab). The forward miRNAs primers were designed based on the full miRNAs sequence, and the reverse primer was the universal reverse primer for miRNA [45]. The stem-loop primer, used for miRNA cDNA synthesis, was designed according to Cheng et al. [45]. The stem-loop sequence consisted of 44 conserved and six variable nucleotides that were specific to the 3' end of the miRNA sequence (5' GTCGTATCCAGTGCAGGGTCCGAGGTATTCGCACTGGATACGACNNNNNN 3'). The RT-qPCR was performed in an ABI 7500 Real-Time PCR System (Applied Biosystems) using SYBR Green I (Invitrogen) to detect double-stranded cDNA synthesis. Reactions were completed in a volume of 24 μL containing 12 μL of diluted cDNA (1:50), 1X SYBR Green I (Invitrogen), 0.025 mM dNTP, 1X PCR Buffer, 3 mM MgCl_2_, 0.25 U Platinum Taq DNA Polymerase (Invitrogen) and 200 nM of each reverse and forward primer. The universal reverse primer (5' GTGCAGGGTCCGAGGT 3') was used in all RT-qPCR reactions. Samples were analyzed in biological triplicate in a 96-well plate, and a no-template control was included. We used MIR156b (5'- TGACAGAAGAGAGAGAGCACA - 3'), MIR172ab (5'- AGAATCTTGATGATGCTGCAT - 3') and MIR1520d (5'- ATCAGAACATGACACGTGACAA - 3') as reference genes, which has been demonstrated as optimal normalizers for water deficit and rust-stress analysis in *Glycine max *[46]. The conditions were set as the following: an initial polymerase activation step for 5 minutes at 94°C, 40 cycles for 15 seconds at 94°C for denaturation, 10 seconds at 60°C for annealing and 25 seconds at 72°C for elongation. A melting curve analysis was programmed at the end of the PCR run over the range 65-99, increasing the temperature stepwise by 0.4°C. Threshold and baselines were manually determined using the ABI 7500 Real-Time PCR System SDS Software v2.0. To calculate the relative expression of the miRNAs, we used the 2^-ΔΔCt ^method. Student's *t*-test was performed to compare pair-wise differences in expression. The parameters of two-tailed distribution and two samples assuming unequal variances were established. The means were considered significantly different when *P *< 0.05.

### Prediction of miRNA targets

Target prediction for miRNAs is straightforward because it is assumed that most of them match their targets with almost perfect complementarity [8,9]. The putative target genes for all miRNAs identified were searched for by using the web-based computer psRNA Target Server (http://biocomp5.noble.org/psRNATarget/) [47] which can identify putative targets that may be regulated at post-transcriptional or at translational levels. Mature miRNA sequences were used as queries to search for potential target mRNAs in the *Glycine max *database (DFCI gene index release 15). The total scoring for an alignment was calculated based on the miRNA length, and the sequences were considered to be miRNA targets if the total score were less than 3.0 points (mismatch = 1 and G:U = 0.5). Results from these analyses were individually inspected on the Phytozome, where the loci and protein annotation were obtained. In order to look for evidences of the predicted targets of the novel identified miRNA, we searched for the miRNA targets sites in the soybean degradome libraries published by Song et al. [31] available under NCBI-GEO accession nμ. GSE25260. Finally, all putative targets regulated by soybean new miRNAs which were analyzed by RT-qPCR were subjected to AgriGO database to investigate the gene ontology [48].

## Results

To identify miRNAs from soybean under water deficit and rust stresses, we generated eight libraries of small RNAs species. From these libraries, a total of 256 miRNAs ranged from 19 to 24 nt-long sequence sizes were identified (Table 1). All pre-miRNA sequence candidates that were selected by the parameters stipulated during the miRNA prediction and those that had no more than five matches on the soybean genome were folded using the Mfold program. All miRNA sequences with the respective precursor sequence originating at a hairpin structure were submitted to the miRBase to determine if they were a new or known miRNA. We separated the results of these miRNAs according the following classes: novel miRNAs belonging to miRNAs families never detected before (29 miRNAs); new miRNAs belonging to conserved miRNA families in other plants species detected for the first time in soybean (15 miRNAs); miRNAs belonging to conserved miRNA soybean families (71 miRNAs); different isoforms of new and known miRNAs (121 isoforms); and known miRNAs already deposited into the miRBase database (20 miRNAs) (Table 1).

**Table 1:** The amount of different miRNA classes detected by high-throughput sequencing.

Class	Size (nt)						Total
		
	19	20	21	22	23	24	
Novel miRNAs	4	3	12	5	1	4	29
New miRNAs pertencing to conserved miRNAs families in other plants species	1	2	9	3	-	-	15
New miRNAs identified in conserved soybean miRNAs families	1	7	52	9	2	-	71
Isoforms of new and known miRNAs	24	50	26	16	4	1	121
Known miRNAs	-	1	16	3	-	-	20

miRNAs detected	30	63	115	36	7	5	256

### Identification of novel miRNAs from soybean

A total of 29 new miRNAs belonging to 24 novel families (Table 2) were identified by Solexa sequencing in libraries from water deficit and rust infections of *Glycine max*. These families were provisory nominated Seq01 to Seq25 (Table 2). The precursor miRNA sequences varied from 55 to 239 nt in length. Precursors of these novel miRNAs were identified, and they formed proper secondary hairpin structures, with MFEs ranging from -16.50 to -153.80 kcal/mol (Additional file 1). The most abundant mature miRNAs were 21 nt in length. We also evaluated the genomic location of the new miRNAs (Table 2). Of the 29 new miRNAs genes identified in soybean, around 86% (25) were located in intergenic regions and the rest were situated inside genes. The mature miRNAs sequences were localized inside the stem-loop sequence with almost half in each arm: 17 miRNAs were localized in the 3' arm and 12 miRNAs were in the 5' arm. More than 63% of the pre-miRNA sequences were in the same sense direction (+) as the soybean genome annotation. For all 24 novel families identified, four were compounded by miRNAs provided from two loci, and we detected only one miRNA member for the rest. Sense and anti-sense miRNAs were detected only in one family, the Seq10, and both were nominated according the arm localization (3p or 5p). Most of the new mature miRNA sequences presented a uracil (U) as their first nucleotide, which is in agreement with previous results for soybean root sequences [26].

**Table 2:** The novel soybean microRNA families determined from Solexa sequencing.

Sequence Code^a^	Mature miRNA		Pre-miRNA						Region^b^
				
	Sequence	Size (nt)	Ch	Start	End	Length (nt)	Sense	Arm	
gma-MIR-Seq01	GGACAGUCUCAGGUAGACA	19	Gm04	30764003	30764171	169	-	3p	intergenic
gma-MIR-Seq03	UGAGAAAAGGAGGAUGUCA	19	Gm11	29821812	29821926	115	+	3p	intergenic
gma-MIR-Seq04a	GCUGGAUGUCUUUGAAGGA	19	Gm08	46853906	46853991	86	+	3p	intergenic
gma-MIR-Seq04b	GCUGGAUGUCUUUGAAGGA	19	Gm18	61624611	61624690	80	-	3p	intergenic
gma-MIR-Seq05	AACCCUCAAAGGCUUCCUAG	20	Gm18	61626669	61626771	103	+	5p	intergenic
gma-MIR-Seq06	AGUGGAACUUUGAGGCCUGC	20	Gm08	46848259	46848354	96	+	3p	intergenic
gma-MIR-Seq07	AAAUGACUUGAGAGGUGUAG	20	Gm01	44787899	44787988	90	+	5p	intergenic
gma-MIR-Seq08	CUAAAGAUUGUCCAAAAGGAA	21	Gm14	6763304	6763456	153	+	5p	intergenic
gma-MIR-Seq09	GUAGUGGAUGCCUAGAGGUCC	21	Gm18	61655979	61656075	97	-	3p	intergenic
gma-MIR-Seq10-5p	UAGGAAUUAGUCACUCAGAUC	21	Gm15	31542836	31543058	223	+	5p	intergenic
gma-MIR-Seq10-3p	AUCUCAGUGACUAAUUUCUAG	21	Gm15	31542836	31543058	223	+	3p	intergenic
gma-MIR-Seq11	UUGUUCGAUAAAACUGUUGUG	21	Gm16	5744795	5744863	69	-	5p	intergenic
gma-MIR-Seq12	UCUCUUGAUUCUAGAUGAUGU	21	Gm16	27653048	27653102	55	+	3p	CDS
gma-MIR-Seq13	UGUUGCGGGUAUCUUUGCCUC	21	Gm04	28578972	28579075	104	-	5p	intergenic
gma-MIR-Seq14a	UGAGAAUUUGGCCUCUGUCCA	21	Gm09	28264427	28264515	89	+	5p	intergenic
gma-MIR-Seq14b	UGAGAAUUUGGCCUCUGUCCA	21	Gm09	28272488	28272562	75	+	5p	intergenic
gma-MIR-Seq15a	UUAGAUUCACGCACAAACUUG	21	Gm02	1041996	1042084	89	+	3p	intergenic
gma-MIR-Seq15b	UUAGAUUCACGCACAAACUUG	21	Gm10	1085223	1085322	100	+	3p	intergenic
gma-MIR-Seq16	UUAUAGUCUGACAUCUGGAAU	21	Gm05	9279518	9279737	220	+	5p	intergenic
gma-MIR-Seq17	ACUAUAGAAGUACUUGUGGAGC	22	Gm16	2916844	2917034	191	+	5p	CDS/intronic
gma-MIR-Seq18	CCUCAUUCCAAACAUCAUCUAA	22	Gm09	16565935	16566025	91	-	3p	intergenic
gma-MIR-Seq19	UGAAGAUUUGAAGAAUUUGGGA	22	Gm15	16900161	16900327	167	+	5p	intronic
gma-MIR-Seq20	CAUCGUUGACGCUGACUGUACG	22	Gm04	35428794	35428950	157	-	5p	5'UTR/intronic
gma-MIR-Seq21	CUGAAGGAUCGAUGUAGAAUGCU	23	Gm02	39825520	39825641	122	+	3p	intergenic
gma-MIR-Seq22	CAUCUGAAGGAUAGAACACAUA	22	Gm09	29816467	29816705	239	+	3p	intergenic
gma-MIR-Seq23	AGUUUCGUGACUACAACUUCUGAA	24	Gm15	16900193	16900294	102	-	3p	intergenic
gma-MIR-Seq24	AUGAAAAUCAUUCAUUAUGAUAUC	24	Gm16	28536014	28536181	168	-	3p	intergenic
gma-MIR-Seq25a	GAAAAUGAAUGAUGAGGAUGGGGA	24	Gm11	7787358	7787494	137	-	3p	intergenic

^a ^The number refers to a new family and the letter refers to the new gene in that family. ^b ^CDS: codon sequence.

### Identification of homologues miRNAs of other plant species

To determine whether any of the miRNAs identified in our libraries were conserved among other plant species, we searched miRBase for homologues. Besides the novel families identified, we also detected 15 miRNAs belonging to six conserved families in other plants species (Table 3). The families MIR170, MIR395, MIR397, MIR408, MIR2118 and MIR3522 were detected for the first time in soybean. For families MIR170 and MIR3522, only a single locus was identified, and for MIR408, three genes were found. In two families, MIR408 and MIR2118, we detected sense and antisense miRNAs (Table 3). MIR170 was only conserved in *Arabidopsis lyrata *and *Arabidopsis thaliana*. MIR408 was found in more different plants species than the other families. It was found in 17 species: *Arabidopsis thaliana, Populus trichocarpa, Pinus taeda, Vitis vinifera, Arachis hypogaea, Arabidopsis lyrata, Citrus sinensis, Oryza sativa, Saccharum officinarum, Zea mays, Physcomitrella patens, Selaginella moellendorffii, Triticum aestivum, Sorghum bicolor, Brachypodium distachyon, Ricinus communis and Aquilegia coerulea *(Table 3). We observed two families (MIR2118 and MIR3522) to be conserved between *Glycine max *and *Glycine soja*; however, we expect that more miRNA families could be conserved between these species considering that they are closely related. This low number is probably due to *Glycine soja *showing fewer miRNAs identified to date.

**Table 3:** New *Glycine max *miRNA families conserved in other plants species.

Family	Acronym	miRNA Sequence	Size (nt)	Species
MIR170	gma-MIR170	UAUUGGCCUGGUUCACUCAGA	21	*ath, aly*

MIR395	gma-MIR395a	CUGAAGUGUUUGGGGGAACUC	21	*ath, ptc, vvi, sly, rco, aly, csi, osa*,
	gma-MIR395b	CUGAAGUGUUUGGGGGAACUC	21	*sbi, mtr, zma, tae, pab*
	gma-MIR395c	CUGAAGUGUUUGGGGGAACUC	21	

MIR397	gma-MIR397a	UCAUUGAGUGCAGCGUUGAUG	21	*ath, osa, ptc, bna, vvi, sbi, bdi, rco*,
	gma-MIR397b	UCAUUGAGUGCAGCGUUGAUG	21	*aly, csi, zma, pab, sly, hvu*

MIR408	gma-MIR408a	AUGCACUGCCUCUUCCCUGGC	21	*ath, ptc, pta, vvi, ahy, aly, csi, osa*,
	gma-MIR408b-5p	CUGGGAACAGGCAGGGCACG	20	*sof, zma, ppt, smo*,
	gma-MIR408b-3p	AUGCACUGCCUCUUCCCUGGC	21	*tae, sbi, bdi, rco, aqc*
	gma-MIR408c	AUGCACUGCCUCUUCCCUGGC	21	

MIR2118	gma-MIR2118a-5p	GGAGAUGGGAGGGUCGGUAAAG	22	
	gma-MIR2118a-3p	UUGCCGAUUCCACCCAUUCCUA	22	*pvc, gso, mtr, osa, zma*
	gma-MIR2118b-5p	GGAGAUGGGAGGGUCGGUAA	20	
	gma-MIR2118b-3p	UUGCCGAUUCCACCCAUUCCUA	22	

MIR3522	gma-MIR3522a	UGAGACCAAAUGAGCAGCUGA	21	*gso*

*Arabidopsis lyrata (aly), Arabidopsis thaliana (ath), Brassica napus (bna), Ricinus communis (rco), Medicago truncatula (mtr), Phaseolus vulgaris (pvu), Arachis hypogaea (ahy), Glycine soja (gso), Aquilegia coerulea (aqc), Seleginella moellendorffii (smo), Physcomitrella patens (ppt), Pinus taeda (pta), Picea abies (pab), Populus trichocarpa (ptc), Citrus sinensis (csi), Vitis vinifera (vvi), Solanum lycopersicum (sly), Brachypodium distachyon (bdi), Hordeum vulgare (hvu), Oryza sativa (osa), Saccharum officinarum (sof), Selaginella moellendorffii (smo), Sorghum bicolor (sbi), Triticum aestivum (tae)*, and *Zea mays (zma)*.

### Identification of conserved soybean miRNAs

To identify conserved soybean miRNAs, all 256 sequences were searched using BLASTn against the soybean miRNAs in miRBase. We identified 22 families of conserved soybean miRNAs in our libraries. Only 20 miRNA soybean genes that were registered in the miRBase were observed (indicated by the number five in Table 1). From the remaining 71 miRNA genes, 12 were miRNAs antisense (in the opposite arm) to the miRNAs presents in miRBase (indicated as group four in Table 4), and 59 were new members detected from new loci of known families (indicated by number three in Table 4). Of the 12 miRNAs identified from the opposite strand of previously known miRNAs, six were in the 5' arm and six in the 3'arm. For the 59 new members of conserved soybean families, 45 miRNAs were 21 nt in length. The family with the largest number of new miRNA genes (nine genes) was MIR319 (Table 4). Interestingly, in family MIR166, we found three new members with sense and antisense miRNAs. Also, in MIR159, two new genes with sequences originated from both the 3'and 5'arms were identified. One new gene was detected in MIR169, MIR172, MIR396 and MIR482 with mature sequences originated from both the 3'and 5'arms (Table 4). Similar to the observation for the novel soybean miRNAs (Table 2), the new genes in these conserved soybean families were compounded for a majority of mature miRNAs with a uracil as the first nucleotide in the 5' end.

**Table 4:** Families of conserved soybean miRNAs.

Group^a^	miRNA ID	miRNA ID sequence	Size(nt)	Ch	Start	End	Arm	Members registered in miRBase^b^
5	gma-MIR156d	UUGACAGAAGAUAGAGAGCAC	21	Gm08	3891365	3891489	5'	a*,b*,c*,d,e*,f*,g*
3	gma-MIR156h	UUGACAGAAGAUAGAGAGCAC	21	Gm02	7812526	7812628	5'	
3	gma-MIR156i	UUGACAGAAGAUAGAGAGCAC	21	Gm05	38621690	38621813	5'	
3	gma-MIR156j	UUGACAGAAGAGAGUGAGCAC	21	Gm06	4699149	4699240	5'	
3	gma-MIR156k	UUGACAGAAGAUAGAGAGCAC	21	Gm07	9347139	9347259	5'	
3	gma-MIR156l	UUGACAGAAGAUAGAGAGCAC	21	Gm09	37843750	37843864	5'	
3	gma-MIR156m	UUGACAGAAGAGAGUGAGCAC	21	Gm14	10664512	10664600	5'	
3	gma-MIR156n	UUGACAGAAGAGAGUGAGCAC	21	Gm17	37759446	37759535	5'	

5	gma-MIR159a-3p	UUUGGAUUGAAGGGAGCUCUA	21	Gm09	37672410	37672586	3'	a(3'),b(3'),c*,d*
4	gma-MIR159a-5p	GAGCUCCUUGAAGUCCAAUUG	21	Gm09	37672410	37672586	5'	
5	gma-MIR159b-3p	AUUGGAGUGAAGGGAGCUCCA	21	Gm07	5386107	5386292	3'	
4	gma-MIR159b-5p	GAGUUCCCUGCACUCCAAGUC	21	Gm07	5386107	5386292	5'	
3	gma-MIR159e-3p	UUUGGAUUGAAGGGAGCUCUA	21	Gm07	9524917	9525127	3'	
3	gma-MIR159e-5p	GAGCUCCUUGAAGUCCAAUU	20	Gm07	9524917	9525127	5'	
3	gma-MIR159f-3p	AUUGGAGUGAAGGGAGCUCCA	21	Gm16	2794128	2794307	3'	
3	gma-MIR159f-5p	GAGUUCCCUGCACUCCAAGUC	21	Gm16	2794128	2794307	5'	

5	gma-MIR162a	UCGAUAAACCUCUGCAUCCAG	21	Gm06	20176238	20176339	3'	a
3	gma-MIR162b	UCGAUAAACCUCUGCAUCCAG	21	Gm05	7692594	7692698	3'	
3	gma-MIR162c	UCGAUAAACCUCUGCAUCCAG	21	Gm17	10181489	10181607	3'	

5	gma-MIR166a-3p	UCGGACCAGGCUUCAUUCCCC	21	Gm16	1912570	1912715	3'	a(3'),b*
4	gma-MIR166a-5p	GGAAUGUUGUCUGGCUCGAGG	21	Gm16	1912570	1912715	5'	
3	gma-MIR166c-3p	UCGGACCAGGCUUCAUUCCCC	21	Gm02	14340767	14340863	3'	
3	gma-MIR166c-5p	GGAAUGUCGUCUGGUUCGAG	20	Gm02	14340767	14340863	5'	
3	gma-MIR166d-3p	UCGGACCAGGCUUCAUUCCCG	21	Gm08	14990547	14990731	3'	
3	gma-MIR166d-5p	GGAAUGUUGUUUGGCUCGAGG	21	Gm08	14990547	14990731	5'	
3	gma-MIR166e-3p	UCGGACCAGGCUUCAUUCCCG	21	Gm15	3688764	3688931	3'	
3	gma-MIR166e-5p	GGAAUGUUGUUUGGCUCGAGG	21	Gm15	3688764	3688931	5'	
3	gma-MIR166f	UCUCGGACCAGGCUUCAUUCC	21	Gm20	43105394	43105500	3'	

5	gma-MIR167c	UGAAGCUGCCAGCAUGAUCUG	21	Gm07	39778512	39778886	5'	a*,b*,c,d*,e*,f*,g*
3	gma-MIR167h	UGAAGCUGCCAGCAUGAUCUG	21	Gm20	44765096	44765173	5'	

5	gma-MIR168a	UCGCUUGGUGCAGGUCGGGAA	21	Gm09	41353226	42353350	5'	a
3	gma-MIR168b	UCGCUUGGUGCAGGUCGGGAA	21	Gm01	48070311	48070420	5'	

5	gma-MIR169a	CAGCCAAGGAUGACUUGCCGG	21	Gm09	35771804	35771924	5'	a,b*,c*,d*,e*
3	gma-MIR169f-3p	UUUCGACGAGUUGUUCUUGGC	21	Gm02	46876643	46876727	3'	
3	gma-MIR169f-5p	UAGCCAAGAAUGACUUGCCGG	21	Gm02	46876643	46876727	5'	
3	gma-MIR169g	CAGCCAAGAAUGACUUGCCGG	21	Gm09	5263992	5264096	5'	
3	gma-MIR169h	CAGCCAAGAAUGACUUGCCGG	21	Gm14	5324798	5324911	5'	
3	gma-MIR169i	CAGCCAAGGAUGACUUGCCGG	21	Gm10	40332790	40332926	5'	
3	gma-MIR169j	CAGCCAAGGAUGACUUGCCGG	21	Gm13	368563	368441	5'	
3	gma-MIR169k	CAGCCAAGGGUGAUUUGCCGG	21	Gm15	14150069	14150183	5'	
3	gma-MIR169l	CAGCCAAGGAUGACUUGCCGG	21	Gm17	4861963	4861816	5'	

3	gma-MIR171d	UUGAGCCGUGCCAAUAUCACG	21	Gm06	48920631	48920715	3'	a*,b*,c*
3	gma-MIR171e	CGAUGUUGGUGAGGUUCAAUC	21	Gm13	26271135	26271232	5'	
3	gma-MIR171f	CGAUGUUGGUGAGGUUCAAUC	21	Gm17	9101701	9101798	3'	

4	gma-MIR172b-5p	GUAGCAUCAUCAAGAUUCAC	20	Gm13	40401688	40401809	5'	a*,b(3')*,c,d*,e*,f*
5	gma-MIR172c	GGAAUCUUGAUGAUGCUGCAG	21	Gm18	2968986	2969138	3'	
3	gma-MIR172g	GCAGCACCAUCAAGAUUCAC	20	Gm10	31592576	31592689	5'	
3	gma-MIR172h-3p	AGAAUCUUGAUGAUGCUGCAU	21	Gm10	43474725	43474831	3'	
3	gma-MIR172h-5p	GCAGCAGCAUCAAGAUUCACA	21	Gm10	43474725	43474831	5'	
3	gma-MIR172i	GCAGCAGCAUCAAGAUUCACA	21	Gm15	2892962	2893122	5'	
3	gma-MIR172j	GCAGCAGCAUCAAGAUUCACA	21	Gm20	40895747	40895836	5'	

3	gma-MIR319d	UUGGACUGAAGGGAGCUCCUUC	22	Gm02	43885398	43885595	3'	a*,b*,c*
3	gma-MIR319e	UUGGACUGAAGGGAGCUCCCU	21	Gm02	45704227	45704412	3'	
3	gma-MIR319f	UUGGACUGAAGGGGAGCUCCUUC	23	Gm04	46348798	46348991	3'	
3	gma-MIR319g	UUGGACUGAAGGGAGCUCCCU	21	Gm11	1374020	1374198	3'	
3	gma-MIR319h	UUGGACUGAAGGGAGCUCCCU	21	Gm11	32902062	32902247	3'	
3	gma-MIR319i	UUGGACUGAAGGGAGCUCCCU	21	Gm14	47959350	47959535	3'	
3	gma-MIR319j	UUGGACUGAAGGGAGCUCCUUC	22	Gm14	45953433	45953649	3'	
3	gma-MIR319k	UUGGACUGAAGGGAGCUCCUUC	22	Gm17	9436178	9436279	3'	
3	gma-MIR319l	UUGGACUGAAGGGAGCUCCCU	21	Gm18	4278883	4279072	3'	

4	gma-MIR396a-3p	UUCAAUAAAGCUGUGGGAAG	20	Gm13	26338134	26338273	3'	a,b(5'),c,d(3')*,e*
5	gma-MIR396a-5p	UUCCACAGCUUUCUUGAACUG	21	Gm13	26338134	26338273	5'	
4	gma-MIR396b-3p	GCUCAAGAAAGCUGUGGGAGA	21	Gm13	26329931	26330056	3'	
5	gma-MIR396b-5p	UUCCACAGCUUUCUUGAACUU	21	Gm13	26329931	26330056	5'	
5	gma-MIR396c	UUCCACAGCUUUCUUGAACUU	21	Gm13	43804777	43804893	5'	
4	gma-MIR396d-5p	UUCCACAGCUUUCUUGAACUU	21	Gm17	9053051	9053155	5'	
3	gma-MIR396f	UCCACAGCUUUCUUGAACUG	20	Gm14	13971419	13971566	5'	
3	gma-MIR396g	UUCCACAGCUUUCUUGAACUU	21	Gm15	556707	556796	5'	
3	gma-MIR396h-3p	GUUCAAUAAAGCUGUGGGAAG	21	Gm17	9044850	9044984	3'	
3	gma-MIR396h-5p	UUCCACAGCUUUCUUGAACUG	21	Gm17	9044850	9044984	5'	

4	gma-MIR482b-3p	UCUUCCCUACACCUCCCAUACC	22	Gm20	35360312	35360406	3'	a*,b(5')
5	gma-MIR482b-5p	UAUGGGGGGAUUGGGAAGGAAU	22	Gm20	35360312	35360406	5'	
3	gma-MIR482c	AUUUGUGGGAAUGGGCUGAUUGG	23	Gm18	61452904	61453003	5'	
3	gma-MIR482d-3p	UCUUCCCUACACCUCCCAUACC	22	Gm10	48569629	48569723	3'	
3	gma-MIR482d-5p	UAUGGGGGGAUUGGGAAGGAAU	22	Gm10	48569629	48569723	5'	

5	gma-MIR1507a	UCUCAUUCCAUACAUCGUCUGA	22	Gm13	25849777	25849883	3'	a,b
5	gma-MIR1507b	UCUCAUUCCAUACAUCGUCUG	21	Gm17	6190604	6190701	3'	

5	gma-MIR1508b	UAGAAAGGGAAAUAGCAGUUG	21	Gm09	28530168	28530271	3'	a*,b

5	gma-MIR1509a	UUAAUCAAGGAAAUCACGGUCG	22	Gm17	10099759	10099869	5'	a, b*

4	gma-MIR1510b	AGGGAUAGGUAAAACAACUACU	22	Gm02	6599299	6599392	5'	a*,b(3')
5	gma-MIR1510b	UGUUGUUUUACCUAUUCCACC	21	Gm02	6599299	6599392	3'	

3	gma-MIR1512b	UAACUGGAAAUUCUUAAAGCAU	22	Gm02	8618692	8618781	5'	a*

5	gma-MIR1513a	UGAGAGAAAGCCAUGACUUAC	21	Gm07	43245809	43245901	5'	a
3	gma-MIR1513b	UAUGAGAGAAAGCCAUGAC	19	Gm17	1401433	1401523	5'	
3	gma-MIR1513c	AAAGCCAUGACUUACACACGC	21	Gm20	223679	223766	3'	

4	gma-MIR2109a	GGAGGCGUAGAUACUCACACCU	22	Gm04	28532441	28532537	3'	a(5')*

4	gma-MIR4376a-3p	AGCAUCAUAUCUCCUGCAUAG	21	Gm13	40845925	40846034	3'	a(5')*

5	gma-MIR4413a	AAGAGAAUUGUAAGUCACUG	20	Gm19	1788518	1788617	5'	a
3	gma-MIR4413b	UAAGAGAAUUGUAAGUCACU	20	Gm13	5170460	5170527	5'	

4	gma-MIR4415a-3p	UUGAUUCUCAUCACAACAUGG	21	Gm18	60474198	60474369	3'	a(5')*
3	gma-MIR4415b	UUGAUUCUCAUCACAACAUGG	21	Gm08	23142767	23142922	3'	

^a ^The group number refers to: (3) the new miRNAs identified in the conserved soybean miRNAs families; (4) miRNAs originated from the opposite arm of miRNAs previously identified; and (5) miRNAs registered in the miRBase that were detected in our libraries. ^b ^* miRNAs registered in the miRBase database that were not detected in our libraries.

### Identification of miRNAs isoforms

Isoforms of microRNAs (isomiRNAs) are a population of known miRNA variants. They are caused by an imprecise or alternative cleavage of Dicer during pre-miRNA processing [10]. We detected numerous miRNAs with additional nucleotides in the 5'or 3' terminus compared to the recorded mature miRNAs. As isomiRNAs were previously reported in soybean high-throughput sequencing [31], we found 121 isomiRNAs in our libraries (Table 5). These isoforms were observed in 22 conserved miRNA families and in four novel families. These miRNA isoforms occurred in both strands from the 5' or 3' arm. The conserved MIR1507a and MIR1507b were found with the most isomiRNAs detected (eight isoforms each). MIR1507a showed a variation of three nucleotides in the 5'end and six nucleotides in the 3'end, and MIR1507b showed a variation of three and five nucleotides in the 5'and 3' terminal region respectively (Table 5). From the novel miRNAs identified, the MIR-Seq07 was the read with the most isoforms detected in our sequencing. This miRNA presented a total of 14 different sequences with 14 varying nucleotides in both the 5'and 3' ends from six fixed nucleotides (Table 5). All isoforms and their respective nominated mature miRNAs can be found in Additional File 1.

**Table 5:** miRNA isoforms identified in the soybean.

Group^a^	Acronym	Sequence^b^	N isos^c^	Pre-miRNA		
				Ch	Start	End
				
5	gma-MIR156g	**+2**/ACAGAAGATAGAGAGCAC/**+2**	2	Gm19	8895390	8895493
5	gma-MIR159a-3p	**+2**/TGGATTGAAGGGAGCTCT/**+1**	4	Gm09	37672410	37672586
4	gma-MIR159a-5p	GAGCTCCTTGAAGTCCAATT/**+1**	2	Gm09	37672410	37672586
3	gma-MIR159e-3p	**+2**/TGGATTGAAGGGAGCTC/**+2**	5	Gm07	9524917	9525127
3	gma-MIR166f	TCTCGGACCAGGCTTCATTC/**+1**	2	Gm20	43105394	43105500
5	gma-MIR167g	TGAAGCTGCCAGCATGATCTG/**+1**	2	Gm10	39044877	39044954
3	gma-MIR169g	**+1**/AGCCAAGAATGACTTGCCGG	2	Gm09	5263992	5264096
3	gma-MIR169h	**+1**/AGCCAAGAATGACTTGCCGG	2	Gm14	5324798	5324911
5	gma-MIR172c	**+1**/GAATCTTGATGATGCTGCAG	2	Gm18	2968986	2969138
5	gma-MIR172d	**+1**/GAATCTTGATGATGCTGCAG/**+3**	3	Gm14	5548752	5548901
5	gma-MIR172e	**+1**/GAATCTTGATGATGCTGCAG/**+3**	3	Gm11	35957808	35957960
3	gma-MIR172h-5p	GCAGCAGCATCAAGATTCAC/**+1**	2	Gm10	43474725	43474831
3	gma-MIR172i	GCAGCAGCATCAAGATTCAC/**+1**	2	Gm15	2892962	2893122
3	gma-MIR172j	GCAGCAGCATCAAGATTCAC/**+1**	2	Gm20	40895747	40895836
5	gma-MIR319a	TTGGACTGAAGGGAGCTCCC/**+1**	2	Gm05	40832097	40832279
5	gma-MIR319b	TTGGACTGAAGGGAGCTCCC/**+1**	2	Gm08	1647815	1647987
3	gma-MIR319d	**+2**/GGACTGAAGGGAGCTCCTTC	2	Gm02	43885398	43885595
3	gma-MIR319f	**+1**/TGGACTGAAGGGGAGCTCCTTC	2	Gm04	46348798	46348991
3	gma-MIR319j	**+2**/GGACTGAAGGGAGCTCCTTC	2	Gm14	45953433	45953649
3	gma-miR319k	**+2**/GGACTGAAGGGAGCTCCTTC	2	Gm17	9436178	9436279
4	gma-MIR396a-3p	**+1**/TTCAATAAAGCTGTGGGA/**+2**	3	Gm13	26338134	26338273
5	gma-MIR396a-5p	**+1**/TCCACAGCTTTCTTGAACTG	2	Gm13	26338134	26338273
4	gma-MIR396b-3p	**+1**/CTCAAGAAAGCTGTGGGAGA	2	Gm13	26329931	26330056
5	gma-MIR396d-3p	**+4**/AAGAAAGCTGTGGGAGA/**+7**	3	Gm17	9053051	9053155
4	gma-MIR396d-5p	TTCCACAGCTTTCTTGAACT/**+1**	2	Gm17	9053051	9053155
5	gma-MIR396e	**+1**/TCCACAGCTTTCTTGAACT/**+2**	4	Gm17	35366535	35366668
3	gma-MIR396g	TTCCACAGCTTTCTTGAACT/**+1**	2	Gm15	556707	556796
3	gma-MIR396h-3p	**+1**/TTCAATAAAGCTGTGGGA/**+2**	3	Gm17	9044861	9044973
3	gma-MIR396h-5p	**+1**/TCCACAGCTTTCTTGAACT/**+1**	3	Gm17	9044850	9044984
5	gma-MIR482a-5p	**+12**/AATGGGCTGATTGG/**+5**	5	Gm01	7783819	7783913
5	gma-MIR482b-5p	**+1**/ATGGGGGGATTGGGAAGGA/**+2**	4	Gm20	35360312	35360406
3	gma-MIR482d-5p	TATGGGGGGATTGGGAAGGA/**+2**	3	Gm10	48569629	48569723
5	gma-MIR1507a	**+3**/CATTCCATACATCGTC/**+6**	8	Gm13	25849777	25849883
5	gma-MIR1507b	**+3**/CATTCCATACATCGTC/**+5**	8	Gm17	6190604	6190701
5	gma-MIR1508a	**+4**/GAAAGGGAAATAGCAGT/**+2**	6	Gm16	32903737	32903831
5	gma-MIR1508b	**+2**/GAAAGGGAAATAGCAGTTG	3	Gm09	28530168	28530271
5	gma-MIR1509b	TTAATCAAGGAAATCACGGTT/**+1**	2	Gm05	7774098	7774206
5	gma-MIR1510a	**+3**/TTGTTTTACCTATTCCA/**+6**	7	Gm16	31518908	31519000
5	gma-MIR1510b-3p	TGTTGTTTTACCTATTCCA/**+3**	4	Gm02	6599299	6599392
4	gma-MIR1510b-5p	**+3**/GATAGGTAAAACAACTA/**+2**	5	Gm02	6599299	6599392
5	gma-MIR1511	AACCAGGCTCTGATACCATG/**+1**	2	Gm18	21161236	21161334
5	gma-MIR1514a	TTCATTTTTAAAATAGGCATT/**+1**	2	Gm07	43175810	43175908
5	gma-MIR1523	**+1**/ATGGGATAAATGTGAGCTC/**+1**	2	Gm02	12253303	12253397
5	gma-MIR2109a-5p	TGCGAGTGTCTTCGCCTCTG/**+1**	2	Gm04	28532441	28532537
4	gma-MIR2109a-3p	**+2**/AGGCGTAGATACTCACAC/**+2**	4	Gm04	28532441	28532537
5	gma-MIR4345	**+9**/ACTTACAAAGAT/**+12**	3	Gm14	49069099	49069193
5	gma-MIR4413a	**+1**/AAGAGAATTGTAAGTCACT/**+1**	3	Gm19	1788518	1788617
3	gma-MIRSeq07	**+14**/GACTTG/**+14**	14	Gm01	44787899	44787988
3	gma-MIRSeq14b	**+2**/AGAATTTGGCCTCTGTCCA	2	Gm09	28272488	28272562
3	gma-MIRSeq10-3p	**+20**/G/**+20**	4	Gm15	31542836	31543058
3	gma-MIRSeq20	CATCGTTGACGCTGACTGT/**+3**	2	Gm04	35428794	35428950
2	gma-MIR408a	**+1**/TGCACTGCCTCTTCCCTGGC	2	Gm02	837416	837548
2	gma-MIR408c	**+1**/TGCACTGCCTCTTCCCTGGC	2	Gm10	36557005	36557130
2	gma-MIR2218a-5p	GGAGATGGGAGGGTCGGTAA/**+2**	2	Gm10	48574017	48574137
2	gma-MIR3522a	**+8**/AGACCAAATGAGC/**+6**	4	Gm15	4318787	4318873

^a ^The group number refers to: (2) the miRNAs previously identified in other plant species as described in Table 2; (3) the new miRNAs identified in the families of conserved miRNAs in soybean; (4) miRNAs originated from the opposite arm of miRNAs previously identified; and (5) miRNAs registered in the miRBase database that were detected in our libraries. ^b ^Sequence conserved between all isoforms and the number of nucleotide variations in each end. ^c ^Total number of isoforms (isos) including the typical member for that gene.

### Validation of miRNAs validation and expression profile by RT-qPCR

**Figure 1:**
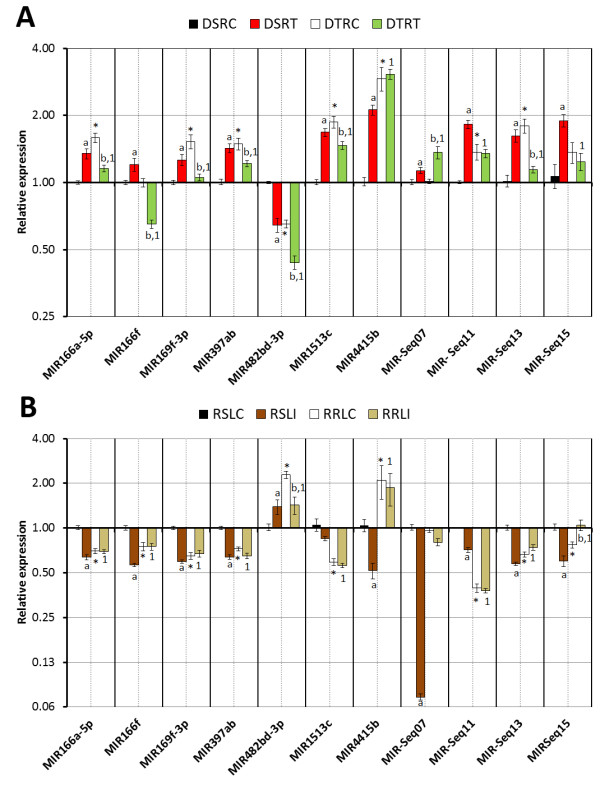
**Effects of biotic and abiotic stresses on miRNA relative expression evaluated by RT-qPCR**. A) Comparative analyses of four libraries from the water deficit experiment. For the water deficit-stress assay, the four libraries were named as: DSRC (drought-sensitive seedlings root submitted to 0 minutes of stress); DSRT (drought-sensitive seedlings root submitted to 125 minutes and 150 minutes of stress); DTRC (drought-tolerant seedlings root submitted to 0 minutes of stress) and DTRT (drought-tolerant seedlings root submitted to 125 minutes and 150 minutes of stress). B) Comparative analyses of four libraries from the rust infection experiment. For the rust-stress assay, the four libraries were named as: RSLC (rust-susceptible seedlings leaves mock inoculation); RSLI (rust-susceptible seedlings leaves with rust-spore inoculation); RRLC (rust-resistant seedlings leaves with mock inoculation) and RRLT (rust-resistant seedlings leaves with rust-spore inoculation). Samples that significantly differs (P < 0.05) according to a Students t-test statistical analysis, were label as: "*" effective differences between cultivars in control conditions; "a" effective differences between control and stressed conditions for sensitive or susceptible plants; "b" effective differences between control and stressed conditions for tolerant or resistant plants and "1" when an effective difference was also observed between sensitive or susceptible and tolerant or resistant under stress conditions.

The stem-loop RT-qPCR was used to validate and measure the expression of the respective miRNAs: MIR166a-5p, MIR166f, MIR169f-3p, MIR397ab, MIR482bd-3p, MIR1513c, MIR4415b, MIR-Seq07, MIR-Seq11, MIR-Seq13 and MIR-Seq15ab, detected by Solexa sequencing. These miRNAs were validated in all genotypes analyzed during dehydration and rust stress. The relative expressions of these miRNAs in the same eight conditions are shown in Figure 1.

#### Expression patterns of miRNAs during water deficit

To identify water deficit-responsive miRNAs, we compared the expression profiles of the 11 miRNAs in both genotypes before and after stress (Figure 1A). A set of five different miRNAs (*MIR166*-*5p*, *MIR169f*-*3p*, *MIR1513c*, *MIR397ab *and *MIR*-*Seq13*) presented the same behavior during the water deficit stress. These miRNAs were commonly up-regulated during the stress condition in the sensitive genotype, and the opposite occur in the tolerant genotype, where they were down-regulated during the water deficit. *MIR*-*Seq11 *and *MIR*-*Seq15 *demonstrated a similar expression across the four conditions. Water deficit significantly increased *MIR*-*Seq11 *and *MIR*-*Seq15 *expression in the roots compared to the control condition in the sensitive genotype, but both miRNAs did not vary in the tolerant plants. *MIR166f *had its level increased in the sensitive genotype and decreased in the tolerant during the stress compared to the control situation. Interestingly, both genotypes presented the same level during the control condition. In the sensitive plants, *MIR*-*482bd*-*3p *showed a strong decrease when submitted to water deficit, being this low level equally observed in the tolerant genotype during the control condition and decreasing when subjected to stress. *MIR4415b *presented an effective rise in its expression level during the water deficit in the sensitive plants, and its high level was also observed in the tolerant genotype independent of the condition. Both sensitive and tolerant genotype exhibited the same expression pattern for *MIR*-*Seq07 *and its level was increased during the stress compared to the control situation.

#### Expression patterns of miRNAs during soybean rust stress

The RT-qPCR analyses of four libraries from the rust assays are shown in Figure 1B. The differential expression analyses revealed that *MIR166a*-*5p*, *MIR166f*, *MIR169*-*3p*, *MIR397ab *and *MIR*-*Seq13 *were dow-regulated in the susceptible genotype during pathogen infection, and equally expressed in the resistant plants. The level of *MIR482bd*-*3p *did not vary significantly between the two different conditions in the susceptible. However in the resistant genotype, its level is higher during the control condition and decrease with the pathogen attack. *MIR1513c *presented unchangeable expression in the control and stressed condition for both genotypes, but when we compared the two genotypes; the resistant was down-regulated compared to the susceptible. A strong decrease was observed for *MIR4415b *in the rust infection when compared with the control in the susceptible plants, and its level is higher in the resistant genotype showing no expression alteration between the conditions. *MIR*-*Seq07 *was down-regulated with respect to the soybean rust infection in both genotypes. Significant difference was observed in *MIR-Seq11 *expression between the mock and infected plants from the susceptible genotypes. This miRNA presented a low expression level after rust inoculation, and its level decreased in the resistant genotypes remaining similar in the both conditions. *MIR*-*Seq15ab *expression level was significantly decreased in the rust compared to the mock treatment in the susceptible genotype, the opposite occurs in the resistant genotype, when the control showed a lower level of expression compared to the stressed condition.

### Target prediction of the soybean miRNAs

MiRNAs suppress gene expression by inhibiting translation, promoting mRNA decay or both [9]. Target gene identification is challenging due to many factors including the following: binding to their target mRNAs by partial complementarity over a short sequence, suppression of an individual target genes is often small, and targeting rules are not completely understood. We predicted the potential miRNAs targets in the psRNA database using all identified miRNAs as queries. The results of the analysis were divided into two tables, showing the targets predicted for the novel (Table 6) and for the conserved miRNAs families (Additional file 2).

**Table 6:** Predicted *Glycine max *mRNA targets for the novel miRNAs.

miRNA ID	Locus target^a^	Target description^a^	miRNA/mRNA pairing^b^
gma-MIR-Seq01	Glyma13g01690	glucuronosyl/glucosyl transferase	- | | | | | | | | | | | | | | | - : |
	Glyma14g35220	glucuronosyl/glucosyl transferase	- | | | | | | | | | | | | | | | - : |
	Glyma15g00330	GTPase-activating protein	| | | - | | : | | | | | | | | | | - |
gma-MIR-Seq03	Glyma08g22900	LRR-containing proteins	- | | | | | | | | | : | | | | | | : |
	Glyma07g03200	LRR-containing proteins	- | | | | | | | | | : | | | | | | : |
	Glyma05g33790	methyltransferase	| - | : | | | | | | | | | | | : | : |
	Glyma04g00810	EF-hand-containing proteins	| | : | | | | | - : : | | | | | | | |
	Glyma11g34320	EF-hand-containing proteins	| | : | | | : | | | : - | | | | | | |
	Glyma10g06740	triosephosphate isomerase	-- | | | | : | | | | | | | | | | | |
gma-MIR-Seq05	Glyma07g18570	pyruvate decarboxylase	| - | | - : | | | | | | | | | | | | | |
	Glyma01g29190	pyruvate decarboxylase	| - | | - : | | | | | | | | | | | | | |
	Glyma18g43460	pyruvate decarboxylase	| - | | - : | | | | | | | | | | | | | |
gma-MIR-Seq06	Glyma08g37480	mt transcription factor	| : : - | | | | | | | : | | | | | | : |
	Glyma16g26070	serine carboxypeptidase	| | | | | - | - | : | : | | | | | | | |
gma-MIR-Seq07	Glyma04g01020	fructose-bisphosphate aldolase	| | | | - | : | | | | | | | | | | | - |
	Glyma16g05500	LRR-containing proteins	| | : | | : : | : | : | : | | | | | | |
	Glyma19g27280	LRR-containing proteins	| | : | | : : | : | : | : | | | | | | |
	Glyma19g07240	translation elongation factor	| | - | | | - | | | | | | | | | | | | -
gma-MIR-Seq08	Glyma14g23860	oxidoreductase activity	| | - | | | | | | | | | | | | | | | | - |
	Glyma13g03430	oxidoreductase activity	| | - | | | | | | | | | | | | | | | | - |
	Glyma01g20670	nucleotide excision repair factor	| | | | | | | | | - | | | : | | | | | : -
gma-MIR-Seq10	Glyma04g09770	mt oxoglutarate/malate carrier	| : | | | : : | | | | | | : | : | | | | :
gma-MIR-Seq11	Glyma15g13500	peroxidase activity	: : | | - | | | | | | | : | : | | | | | |
	Glyma09g02600	peroxidase activity	| : | | - | | | | | | | : | : | | | | | |
gma-MIR-Seq12	Glyma08g20670	ATP-dependent RNA helicase	: | | | | | | | | | | : | | : | | | | | -
	Glyma07g01260	ATP-dependent RNA helicase	: | | | | | | | | | | : | | : | | | | | -
	Glyma20g16950	predicted alpha/beta hydrolase	| | - | | | | | | | | : | | | | | | : | :
	Glyma10g23470	predicted alpha/beta hydrolase	| | - | | | | | | | | : | | | | | | : | :
	Glyma19g35390	serine/threonine protein kinase	| | | | | | | | | -- | | - | | | | | | |
	Glyma03g32640	serine/threonine protein kinase	| | | | | | | | | -- | | - | | | | | | |
gma-MIR-Seq13	Glyma02g26160	oxidoreductase activity	| - | | - | | | | | | | | : | | | | | : |
	Glyma10g31690	transcription regulator activity	| | | | | | | | - | - | | | | | : : | | |
gma-MIR-Seq15	Glyma20g02820	translation initiation factor	-- | | | | | | | | | | | | | | | | | | |
gma-MIR-Seq16	Glyma17g20860	LRR-containing proteins	| | | | | | | | | | | | - | | : | | | | |
	Glyma05g09440	LRR-containing proteins	| | | | | | | | | | | | - | | : | | | | |
gma-MIR-Seq18	Glyma11g21200	LRR-containing proteins	| | : | | | | | | | | | | | | | | | | | --
gma-MIR-Seq19	Glyma15g37290	LRR-containing proteins	- | | | | | - | | | | | | | | | | | | | | -
	Glyma09g34200	LRR-containing proteins	| : | | | : | | | : | - | | | | | : | | | |

^a ^The Data from Phytozome version 6.0. ^b ^Pairing obtained in psRNATarget Server: "|" indicates a Watson-Crick base pairing; ":" is a G:U base pairing, and "-" indicates a mismatch.

Among the 24 novel identified miRNAs families, only 14 families had targets predicted (Table 6). The miRNAs families MIR-Seq01, MIR-Seq03, MIR-Seq06, MIR-Seq07, MIR-Seq08, MIR-Seq12 and MIR-Seq13 had multiple distinct targets. MIR-Seq10, MIR-Seq15 and MIR-Seq18 targeted only one locus. Although, MIR-Seq05, MIR-Seq11, MIR-Seq16 and MIR-Seq19 presented several loci as targets, all of them are coding for the same proteins. Fructose-bisphosphate aldolase, LRR (leucine-rich-repetitions)-containing proteins, translation elongation factor were predicted to be potential targets of the novel MIR-Seq07 which was investigated by RT-qPCR. The search for a target of the novel MIR-Seq11, also analyzed by RT-qPCR, showed a match to *Glycine max *peroxidase precursors mRNAs as potential targets. The oxidoreductase and a transcription regulator factor were predicted to be targeted by MIR-Seq13; and for the MIR-Seq15 only a translation initiator factor was predicted as a target.

After a comparative analysis of our novel identified miRNAs and the degradome libraries of developing soybean seeds it was possible to identify specific sequences in the degradome that corresponds to the downstream sequence of the predicted miRNA recognition site. We identified target sequences to six among the 24 novel soybean miRNAs (MIR-Seq01, MIR-Seq 06, MIR-Seq07, MIR-Seq11, MIR-Seq12 and MIR-Seq16). The list of the 10 identified genes is composed by a glucosyl transferase, serine carboxypeptidase, fructose biphosphate aldolase, three leucine-rich repeat protein, two peroxidases and two ATP dependent RNA helicases (Additional file 3).

Although many soybean conserved miRNAs targets have been predicted and validated by previous studies [26,27,30,31], we also investigated the possible targets for the 28 known families of miRNAs detected in our sequencing. Of these, only 21 families had predicted targets and they are listed in the Additional file 2. The conserved miRNA families showed multiples targets, however families MIR156, MIR172, MIR396, MIR397, MIR1510 and MIR1513 were highly conserved about their targets. For example, all members from the MIR156 family (which had a predicted target) targeted SBP (squamosa promoter binding)-domain protein. AP(2) APETALA 2 transcription factors were targeted by MIR172 family. The same occur with MIR396, MIR397, MIR1510 and MIR1513 families that targeted various genes families as GRF (growth regulating factor) transcription factor, multicopper oxidases, LRR (leucine-rich-repetitions)-containing proteins and F-BOX domain proteins respectively. These results were already observed across several plant species (except for MIR1510 and MIR1513) [25,49-53].

### Gene Ontology analysis

**Figure 2:**
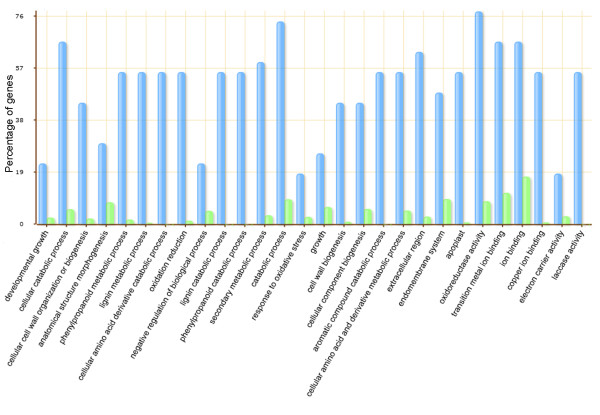
**Go analysis of miRNAs target genes**. Blue bars indicate the enrichment of miRNA targets in GO terms. Green bars indicate the percentage of total annotated soybean genes mapping to GO terms. Only the predicted target genes for miRNAs analyzed by RT-qPCR were considered.

The targets of those miRNAs which the expression was analyzed by RT-qPCR were also investigated in respect to their gene ontology (GO) [48]. Among the 11 miRNA genes, six presented target predictions, which were: MIR397ab, MIR1513c, MIR-Seq07, MIR-Seq11, MIR-Seq13 and MIR-Seq15ab. The putative soybean miRNAs targets presented diverse functions, however the most representative group was the proteins involved in oxidoreductase activity followed by the proteins involved in the catabolic process (Figure 2). The result demonstrates that more than 76% of the target proteins are involved in oxidoreductase activity is consistent with the fact that some of the miRNAs libraries are originated from stressed plants. A consequence of many environmental stresses - including water deficit and pathogen attack - is a oxidative stress, i. e. the accumulation of reactive oxygen species (ROS), which damage cellular structures [49,54]. As miRNAs MIR397, MIR-Seq11 and MIR-Seq13 were predicted to match proteins with oxidative activity, they may act in some level of regulation during water deficit or ASR stress.

## Discussion

The use of deep-sequencing technology was efficient to identify 256 miRNAs of *Glycine max*. These miRNAs were identified from eight different libraries from precursors with stem-loop secondary structures that also map to the soybean genome (Additional file 1). They were detected from water deficit and rust libraries and were characterized as following: detected for the first time, already detected in some plant species, conserved in soybean, or a variant of a known miRNA (isoform). From these analyses, we found 24 novel families that had not been detected before, six families that had already been detected in Coniferophytes, Embryophytes and Magnoliophytes (dicotyledons and monocotyledons), and 22 conserved soybean families. In terms of conserved soybean miRNAs, we only detected 20 known miRNAs in our sequencing. This small number of known miRNA genes detected in our libraries could be due to the two filters used in our processing. These filters may have missed some known, conserved soybean miRNAs because they discarded reads with low frequency and those with more than five matches in the genome.

We detected 121 miRNAs with additional nucleotides in the 3' or 5' terminus compared to the recorded mature miRNA. These miRNA variants (isomiRNAs) were very common in our population of small, detected RNAs. Out of the isomiRNAs, we observed 21 pairs of sense and antisense miRNAs. The duplex presents the antisense strand paired to the corresponding miRNA with two nucleotides 3' overhangs (Additional file 1). This shows that the sense and antisense miRNAs originated from DCL1 processing and supports their validation as true miRNAs [26,55,56].

In addition, we validated the conserved miRNAs in our libraries based on homology to known miRNAs in miRBase. The phylogenetic conservation of miRNA sequences is one rule proposed by Ambros et al. [7] to characterize miRNAs. In this study, we established new miRNAs in soybean that were already detected in other plants species. However, as opposed to some studies that only blast the candidate to the known miRNA mature sequence, our identifications were determined by precursor sequence folding and verification of the genuine hairpin structures.

The complexity of the plant response to biotic and abiotic stresses involves many genes and biochemical and molecular mechanisms, and adaptation to these stresses is achieved through regulating gene expression at the transcriptional and post-transcriptional levels. With regard to post-transcriptional regulation, miRNAs are associated with water deficit response in others plants, but this was the first time that differential expression of these small RNAs were observed in soybean during water deficit. In order to validate 11 of the novel miRNAs detected in sequencing by the RT-qPCR method, we constructed primers stem-loop and analyzed their expression during abiotic and biotic stresses (Figure 1). We observed that several miRNAs were up-regulated during the water deficit in the sensitive genotype (Figure 1A). However, during the same stress, these miRNAs had a different expression in the tolerant genotype. This distinct miRNAs behavior between the two contrasting genotypes under the same conditions could be involved with the drought-tolerance that is observed in the tolerant genotype. One of these miRNAs with this expression pattern is the new MIR-Seq11. Interestingly, MIR-Seq11 was predicted to target peroxidase protein. As known, stress conditions can produce excess concentrations of reactive oxygen species (ROS), resulting in oxidative damage at the cellular level [57]. The increase of this miRNA in the sensitive genotype, when subjected to water deficit, could be one of the factors associated with vulnerability of these sensitive plants. Whereas in tolerant genotype during the two conditions, the expression levels of MIR-Seq11 are lower than in the sensitive cultivar during stress. This situation could indicate that the unchangeable MIR-Seq11 levels in the tolerant genotype may be related to its drought-tolerance capacity.

Another interesting point is the expression of a novel miRNA MIR-Seq07 that showed increased expression levels during the water deficit stress for both genotypes. This result allows us to associate this miRNA with water deficit stress mechanism independently of the genotype background. Our computational approach showed that one of the loci targeted by MIR-Seq07 corresponds to a fructose-bipfosphato-aldolase enzyme which is a constituent of both the glycolytic/gluconeogenic pathway and the pentose phosphate cycle in plants [58]. Therefore increase and/or activation of aldolase appear to be implicated in the plant growth mainly through promotion of the glycolytic pathway function to synthesize ATP [58]. Since, MIR-Seq07 expression was increased during the stress condition in both genotypes and assuming that it can inhibit or degrade aldolases, it could be associated to metabolism decreasing during water deficit in roots.

Plants possess several adaptive traits to support pathogen attacks. In *Glycine max*, ASR is responsible for significant losses in soybean growth areas. Nevertheless, no study investigating miRNAs and ASR disease had been preformed to date. To determine if miRNAs act as key factors during rust infection or for resistance maintenance, we performed expression analyses with the same 11 miRNAs during mock and infected conditions in two different genotypes (Figure 1B). In general the miRNAs under the fungus infection were down-regulated in the susceptible genotype (except MIR482bd-3p). For example, MIR-Seq11, MIR-Seq13 and MIR-Seq15 which had predicted peroxidases, oxidoreductases and translational initiation factor respectively as targets proteins, were down regulated when infected with ASR. The peroxidases enzymes help to metabolize H_2_O_2 _in higher plants, and these proteins, as also others proteins with oxidoreductase activity, have already been reported to be up-regulated after pathogen infection and especially after ASR [57], indicating a possible involvement of MIR-Seq11 and MIR-Seq13 with the responses to ASR infection. Considering, that a translational initiator factor was predicted to be targeted by MIR-Seq15, we could speculate about the participation of this miRNA in the protein synthesis machinery.

In the resistant plants, most of the miRNAs analyzed by RT-qPCR (except MIR482bd-3p, MIR-Seq07, MIR-Seq15ab) did not vary across the mock and rust infection. Surprisingly, MIR-Seq07 was the unique miRNA that was down-regulated during the fungi infection for both genotypes analyzed in our study. We already mentioned that the MIR-Seq07 had predicted protein target related to metabolism and thus its possible association with water stress. However MIR-Seq07 also had predicted LRRs (leucine-rich repeats)-domain target which are known to be present in disease resistance proteins [59,60]. This suggested a good candidate for the investigation of the miRNAs' regulatory mechanisms during ASR stress. Although we investigated the expression patterns of some miRNAs detected in our sequencing and predicted the target genes that it regulates, additional experimental approaches must be addressed to confirm these hypotheses.

## Conclusions

The present study detected a large number of small RNA sequences that were characterized as novel and as already known soybean miRNAs. We grouped some of these unique sequences into 24 novel soybean miRNAs and further classified several of new members in known families or as new loci in the soybean genome. Validation of new miRNA with quantitative RT-qPCR revealed that Solexa sequencing is a powerful tool for miRNA discovery. Many miRNA expression patterns were up- or down-regulated by water deficit and rust stresses, which is an important discovery. Future investigations should use supplementary experimental approaches to verify the targets and to understand the complex gene regulatory network of these miRNAs. This work will contribute to improve systems to support soybean crop production and to mitigating crop losses during biotic or abiotic stresses.

## Authors' contributions

FRK and RM conceived and designed the study. FRK performed the sequence analyses to identify the miRNAs and secondary structures and to predict the target genes conceived, executed the RT-qPCR, performed the data management and processing, and wrote the draft manuscript. RM was the supervisor of this study, provided critical revision, obtained financial support and performed data interpretation. LFVO contributed to the data assembly, prediction and identification de new miRNAs. LM and MA contributed to the analysis of the miRNA secondary structures and processing of the data. FR, JM, JFB and RSM performed the plant experiments and RNA extractions. ALN, FCMG and RVA provided the studied material, critically revised the article for important intellectual content and obtained funding. MFC, GAGP and LCN created the Perl scripts to identify the microRNAs. MFC participated in writing the methods section. All authors read and approved the final version of manuscript.

## Supplementary Material

Additional file 1 **Predicted precursor structures of all miRNAs identified**. The mature miRNAs (red) and pre-miRNA sequences with chromosome and locus information. The pre-miRNA length (nt) and its directional information (sense (+) or anti-sense (-) compared to the soybean genome sequence) is provided. The fold-back structure with respect to the free energy value (dG) was predicted using the Mfold program. Click here for file

Additional file 2 **Identified targets of known conserved plant miRNAs families**. ^a ^The Data from Phytozome version 6.0. ^b ^Pairing obtained in psRNATarget Server: "|" indicates a Watson-Crick base pairing; ":" is a G:U base pairing, and "-"indicates a mismatch. Click here for file

Additional file 3 **The soybean transcript loci which were identified as new-miRNA families target by degradome sequencing**. The miRNA target site is indicated in red and underlined while the degradome sequence is highlighted. Click here for file
