# Supplementary material for: Identification of novel soybean microRNAs involved in abiotic and biotic stresses
Source: BMC Genomics. 2011 Jun 10;12:307. doi: 10.1186/1471-2164-12-307 (PMC3141666; doi:10.1186/1471-2164-12-307)
Supplement: Additional file 1 — Predicted precursor structures of all miRNAs identified. The mature miRNAs (red) and pre-miRNA sequences with chromosome and locus information. The pre-miRNA length (nt) and its directional information (sense (+) or anti-sense (-) compared to the soybean genome sequence) is provided. The fold-back structure with respect to the free energy value (dG) was predicted using the Mfold program. [file 1471-2164-12-307-S1.PDF]

Additional file 1. Predicted precursor structures of all miRNAs identified.

The miRNAs (red) and pre-miRNA sequences with chromosome and locus information. The pre-miRNA length (nt) and its direction information (sense (+) or anti-sense (-) compared to the soybean genome sequence) is provided. The fold-back structure with respect to the free energy value (dG) was predicted using the Mfold program.

Identification of novel miRNAs from soybean

SEQ01 GGACAGUCUCAGGUAGACA

Gm04:30764003-30764171, 169nt, (-)

ACUGUUUCCUGGGAUUGGCUUUGGGCUUUCCUGCACAGCUUAGGUGGAGGGCAAAGAAGACUUCCUUCUGAGGGGGCCAGAGCCAUCAGAGAGAUACCACUCUGGACGAGCUAGAAUUCUAAC
CUUGUGCCAGGACCUAUGGGCCGAGGGACAGUCUCAGGUAGACAGU

```
Structure 4 Folding bases 1 To 169 of 10Sep29-16-53-28     Initial dG = -67.90

         10        20             30         40           50            60
       -          G    G     -----    -     GCUU    -    .-G   AA----     AC
ACUGUUU CCUGGGAUUG CUUU GGCUU     UCCU GCACA    AGG UGGA   GGC      AGAAG  \
||||||| |||||||||| |||| |||||     |||| |||||    ||| ||||   |||      |||||
UGACAGA GGACUCUGAC GGGA CCGGG     AGGA CGUGU    UCC AUCU   CCG      UCUUC  U
       U          A    G     UAUCC    C     ----    A    \ -   GGGGAG     CU
       160       150       140       130           120           70
                                                          80             90
                                                          AG---|  CA--      AGA
                                                                AGC    UCAGAG   U
                                                                |||    ||||||
                                                                UCG    GGUCUC   A
                                                          UAAGA^  AGCA      ACC
                                                                110       100
```

SEQ03 UGAGAAAAGGAGGAUGUCA

Gm11:29821812-29821926, 115nt, (+)

AGCUGAAUCUUUUUUUUCUGAGAGCAUGAUCCCAUGCUGGUUGGCUAUCUCAAAGAAUUGAUGCAACAAUUUUUUAUGAGAGGCAAUAUCUUUUGAGAAAAGGAGGAUGUCAUCU

```
Structure 1 Folding bases 1 To 115 of 10Sep29-17-04-58     Initial dG = -34.80

         10        20          30
  C   -             G     |    A
AG UGA AUCUUUUUUUUCU AGA--GCAUG U
|| ||| ||||||||||||| |||  |||||
UC ACU UAGGAGGAAAAGA UUU  CGUAC C
  U   G             G   \ ^    C
```

```
   110       100
                           40         50          60
                          U   UG-   A     --        AU
                           GGU   GCU UCUCA  AAGAAUUG  G
                           |||   ||| |||||  ||||||||
                           CUA   CGG AGAGU  UUUUUAAC  C
                          U   UAA   -     AU        AA
                          90         80        70
```

SEQ04a GCUGGAUGUCUUUGAAGGA

Gm08:46853906-46853991, 86nt, (+)

GUUCCCUCAAAGGCUUCCAGUAUUCCCGUAUUCAUUCAUAUAUACCUAGCUAGAAUUGAUUGAAUGCUGGAUGUCUUUGAAGGAAC

```
Structure 1 Folding bases 1 To 86 of 10Sep29-17-09-38 Initial dG = -32.60

        10        20         30        40
     C        U          CCGU-|   AUUCAUAUA
GUUCC UCAAAGGC UCCAGUAUUC     AUUC         \
||||| |||||||| ||||||||||     ||||
CAAGG AGUUUCUG AGGUCGUAAG     UAAG         U
     A        U          UUAGU^   AUCGAUCCA
     80        70        60        50
```

SEQ04b GCUGGAUGUCUUUGAAGGA

Gm18:61624611-61624690, 80nt, (-)

UCGUGUUCCCUCAAAGGCUUCCAGUAUUCAUUCAUACCUAACUAGUUGCUUGAAUGCUGGAUGUCUUUGAAGGAAUUUGA

```
Structure 1 Folding bases 1 To 80 of 10Sep29-17-11-26      Initial dG = -35.40

        10        20        30        40
   U     C        U           UU  UACCU
UCG GUUCC UCAAAGGC UCCAGUAUUCA  CA     A
||| ||||| |||||||| |||||||||||  ||
AGU UAAGG AGUUUCUG AGGUCGUAAGU  GU     A
   U     A        U           UC  UGAUC
         70        60        50
```

SEQ05 AACCCUCAAAGGCUUCCUAG

Gm18:61626669-61626771, 103nt, (+)

CAGUAACCCUCAAAGGCUUCCUAGACUCCAUGUUACGGUCAAAUCAAUUAAUCGUUGAUUAGGAAUAAUUAAGAGUUUCGGAAGUAACUUUGGGGGUUAACUG

Structure 1 Folding bases 1 To 103 of 10Sep29-17-12-48   Initial dG = -41.30

```
         10          20        30        40        50
   -            --      U      CAU    CGG  A       UA
CAG UAACCCUCAAAG  GCUUCC AGACUC   GUUA   UC AAUCAAU  \
||| ||||||||||||  |||||| ||||||   ||||   || |||||||
GUC AUUGGGGGUUUC  UGAAGG UUUGAG   UAAU   GG UUAGUUG  A
   A            AA      C      AAU    AA-  A       CU
 100        90        80        70         60
```

SEQ06 AGUGGAACUUUGAGGCCUGC

Gm08:46848259-46848354, 96nt, (+)

CUUUAGCAACCCUCAAAGGCUUCCACUACUCCAUAUUUCAGUCUAGUGAAUGUCCACAAACAUGGAGGAGUAGUGGAACUUUGAGGCCUGCUGAAG

Structure 2 Folding bases 1 To 96 of 10Sep29-17-14-16 Initial dG = -45.10

```
        10        20        30        40        50
        AC        GC            AUAU|   GUCUA   AA
CUUUAGCA  CCUCAAAG  UUCCACUACUCC    UUCA     GUG  U
||||||||  ||||||||  ||||||||||||    ||||     |||
GAAGUCGU  GGAGUUUC  AAGGUGAUGAGG    AGGU     CAC  G
        CC        --            ----^   ACAAA   CU
     90        80          70            60
```

**SEQ07**	AAAUGACUUGAGAGGUGUAG
**SEQ07(iso1)**	CGACUUUGUGAAAUGACUUG
**SEQ07(iso2)**	UGAAAUGACUUGAGAGGUGUAG
**SEQ07(iso3)**	GAAAUGACUUGAGAGGUGUAGG
**SEQ07(iso4)**	GAAAUGACUUGAGAGGUGUAG
**SEQ07(iso5)**	AAUGACUUGAGAGGUGUAGGAU
**SEQ07(iso6)**	AAAUGACUUGAGAGGUGUAGGA
**SEQ07(iso7)**	AAUGACUUGAGAGGUGUAGGA
**SEQ07(iso8)**	AAUGACUUGAGAGGUGUAGG
**SEQ07(iso9)**	AAUGACUUGAGAGGUGUAG
**SEQ07(iso10)**	AUGACUUGAGAGGUGUAGG
**SEQ07(iso11)**	UGACUUGAGAGGUGUAGGA
**SEQ07(iso12)**	GACUUGAGAGGUGUAGGAUA
**SEQ07(iso13)**	UUGAGAGGUGUAGGAUAAG

Gm01:44787899-44788252, 90nt, (+)

UUAGUUCGACUUUGUGAAAUGACUUGAGAGGUGUAGGAUAAGUGGGGAGCAAUCCUCACCUUAUAAGUCGGUUUUGUAGGGUUGAGUUAA

Structure 1 Folding bases 1 To 90 of 10Sep29-17-19-09 Initial dG = -36.60

```
        10         20         30         40
U  GU         UG    -       A      U     AA   GG
 UA  UCGACUUUG  AAA UGACUUG GAGGUG AGGAU  GU   \
 ||  |||||||||  ||| ||||||| |||||| |||||  ||
 AU  AGUUGGGAU  UUU GCUGAAU UUCCAC UCCUA  CG   G
A  UG         GU    G       A      -     A-   AG
         80         70         60          50
```

SEQ08 CUAAAGAUUGUCCAAAAGGAA

Gm14:6756499-6763456,153nt, (+)

GUUUGCACUAAAGAUUGUCCAAAAGGAAUAUUGUUUUAUGCAGAAGACAAAUUACAUCGGAUAAGAAAAAUCACACCACAUAAUUGCUGCAUUGCUUUAGAUCUCUAUUUCUGCAUAAAACAA
UAUUCCUUUUGGACAAUCUUUAGUGCAAAC

Structure 1 Folding bases 1 To 153 of 10Sep29-17-22-09 Initial dG = -73.40

```
        10        20        30        40          50        60
                                              .-ACAAAUUACA|  GG
GUUUGCACUAAAGAUUGUCCAAAAGGAAUAUUGUUUUAUGCAGAAG            UC   A
||||||||||||||||||||||||||||||||||||||||||||||            ||
CAAACGUGAUUUCUAACAGGUUUUCCUUAUAACAAAAUACGUCUUU            AG   U
                                              \ ----------^ AA
 150       140       130       120       110

                                                  70        80        90
                                                AAAAUCACACCACAUAAUU  UG
                                                                   GC   C
                                                                   ||
                                                                   CG   A
                                                AUCUCUAGAUUU-------  UU
                                                     100
```

SEQ09 GUAGUGGAUGCCUAGAGGUCC

Gm18:61655979-61656075, 97nt, (-)

GCUUUUUAGUGGCCUCAAAGGCUUCCACUACUGCAUGUUUCUGUGGUUUAAUAUCCUGAAACAAGUGGUAGUGGAUGCCUAGAGGUCCAUUAAAAGC

Structure 1 Folding bases 1 To 97 of 10Sep29-17-30-58 Initial dG = -48.90

```
        10         20        30        40        50
      U     -    AA    U        UG A      UGU  UUU
GCUUUU AGUGG CCUC  AGGC UCCACUAC  C UGUUUC   GG    A
|||||| ||||| ||||  |||| ||||||||  | ||||||   ||
CGAAAA UUACC GGAG  UCCG AGGUGAUG  G ACAAAG   CC    A
      -     U    A-    U        GU A      U--  UAU
       90         80        70        60
```

SEQ10-5p UAGGAAUUAGUCACUCAGAUC
SEQ10-3p UCUCAGUGACUAAUUUCUAGA
SEQ10-3p iso1 AUCUCAGUGACUAAUUUCUAG
SEQ10-3p iso2 GAAUUUGAGGACUAGGGACCUC
SEQ10-3p iso3 UAAUUUCUAGAAUUUGAGGACU

Gm15:31542836-31543058, 223nt, (+)
GGGAGGCCCCUAGUCCUCGAAUUCUAGGAAUUAGUCACUCAGAUCCUAACCUCUUUGGUUCUUUCAUUGAGAUCCAUGGUUGAGUCUACUCGUUCCAAGCCUAAUAUGGACUGCAUCGAAGAG
GCCAUCGCGAAACUUACCUCCAAUCAACUCAACCUCAUCGCUGCAAAGAUGUUAGGAUCUCAGUGACUAAUUUCUAGAAUUUGAGGACUAGGGACCUCCC

Structure 3 Folding bases 1 To 223 of 10Sep29-18-10-02 Initial dG = -110.20

```
        10        20        30        40        50          60         70                 80
      C                                C          C          UC    AU  -  A    ---------   U-|   U
GGGAGG CCCUAGUCCUCGAAUUCUAGGAAUUAGUCACU AGAUCCUAAC UCUUU--GGU  UUUC  UG AG UCCA         UGG  UGAG C
|||||| |||||||||||||||||||||||||||||||| |||||||||| |||||  |||  ||||  || || ||||         |||  ||||
CCCUCC GGGAUCAGGAGUUUAAGAUCUUUAAUCAGUGA UCUAGGAUUG AGAAA  CCG  GAAG  AC UC AGGU         ACC  GCUC U
      A                                C          U     \    GA    CU  G  -    AUAAUCCGA   UU^   A
 220       210       200       190       180       170        120        110       100        90

                                                           130       140
                                                          AUC    AACUUACCUCCAA
                                                             GCGA            \
                                                             ||||
                                                             CGCU            U
                                                          CGU    ACUCCAACUCAAC
                                                               160       150
```

SEQ11 UUGUUCGAUAAAACUGUUGUG
Gm16:5743687-5744863, 65nt, (-)
UUGCAUGGUUGUUCGAUAAAACUGUUGUGAUAAUGUACAACACAAUUAUCGAUAGCUUAUGCAAA

Structure 1 Folding bases 1 To 65 of 10Sep29-17-37-15 Initial dG = -23.40

```
          10        20        30
-      -      U      AAC       AU
 UUGCAU GGUUGU CGAUAA   UGUUGUG  A
 |||||| |||||| ||||||   |||||||
 AACGUA UCGAUA GCUAUU   ACAACAU  A
A      U      -      AAC       GU
    60         50        40
```

SEQ12 UCUCUUGAUUCUAGAUGAUGU

Gm16:27653048-27653102, 55nt, (+)

UCAUAGAGUCUAGGCUCACGGGAAAGAAGAUUCUCUUGAUUCUAGAUGAUGUUGA

Structure 1 Folding bases 1 To 55 of 10Sep29-17-40-29 Initial dG = -16.50

```
         10        20
  -   GA       C   C     AG
UC AUA  GUCUAGG UCA GGGAA  A
|| |||  ||||||| ||| |||||
AG UGU  UAGAUCU AGU CUCUU  A
  U   AG       U   U      AG
    50        40        30
```

SEQ13 UGUUGCGGGUAUCUUUGCCUC

Gm04:28578972-28579075, 104nt, (-)

GCCAGCAAAACUGUUGCGGGUAUCUUUGCCUCUGAAGGAAAGUUGUGCCUAUUAUUAUGGCUUAUUGCUUUAGUGGCGUAGAUCCCCACAACAGUUAUGCUUGC

Structure 1 Folding bases 1 To 104 of 10Sep29-17-44-58 Initiald G = -47.10

```
        10        20        30          40        50
  C    A        C   U    U    U      G --    UG  CCU
GC AGCA AACUGUUG GGG AUCU UGCC CUGAAG A  AAGU  UG   A
|| |||| |||||||| ||| |||| |||| |||||| |  ||||  ||
CG UCGU UUGACAAC CCC UAGA GCGG GAUUUC U  UUCG  AU   U
  U    A        A   C    U    U      G UA    GU  UAU
  100       90        80        70        60
```

SEQ14a UGAGAAUUUGGCCUCUGUCCA

Gm09:28264427-28264514, 88nt, (+)

AAAUUUUCUUGAGAAUUUGGCCUCUGUCCAUGUCUAAUUAUUAAUUCCAAUAAUUGAGAUGGAUAGAGCCCAAAUUCUCAAGAGAAUUU

Structure 1 Folding bases 1 To 89 of 10Sep29-17-47-47 InitialdG = -48.50

```
        10        20        30        40
                    C          G  U       AA
AAAUUUUCUUGAGAAUUUGG CUCUGUCCAU UC AAUUAUU  U
|||||||||||||||||||| |||||||||| || |||||||
UUUAAGAGAACUCUUAAACC GAGAUAGGUA AG UUAAUAA  U
                    C          G  -       CC
        80        70        60         50
```

SEQ14b UGAGAAUUUGGCCUCUGUCCA
SEQ14b(iso) AGAAUUUGGCCUCUGUCCA

Gm09:28272488-28272562, 75nt, (+)

CUUGAGAAUUUGGCCUCUGUCCAUGUCUAAUUAUUAAUUCCAAUAAUUCAGAUGGACAGAGCCCAAAUUCUCAAG

Structure 1 Folding bases 1 To 75 of 10Sep29-17-49-1 Initial dG = -38.80

```
        10        20         30
             C         UG    -       AA
CUUGAGAAUUUGG CUCUGUCCA  UCU AAUUAUU  U
||||||||||||| |||||||||  ||| |||||||
GAACUCUUAAACC GAGACAGGU  AGA UUAAUAA  U
             C         --    C       CC
    70        60          50         40
```

SEQ15a UUAGAUUCACGCACAAACUUG

Gm02:1041996-1042084, 89nt, (+)

AAAUUGCAGGUUCGUGCGUGAAUCUAACGAAAGUUUCUCGUUCUCUCCAUUCCACUUCUGCGUUAGAUUCACGCACAAACUUGUCAUUU

Structure 1 Folding bases 1 To 89 of 10Sep29-17-53-59 Initial dG = -34.80

```
        10        20        30          40
    U       C                A--    UUCUCGUUC
AAAU GCAGGUU GUGCGUGAAUCUAACG   AAGU         \
|||| ||||||| ||||||||||||||||   ||||
UUUA UGUUCAA CACGCACUUAGAUUGC   UUCA         U
    C       A                GUC    CCUUACCUC
        80        70        60        50
```

SEQ15b UUAGAUUCACGCACAAACUUG

Gm10:1085223-1085322, 100nt, (+)

GAGACAGAGGCAAAUCGCAGGUUCGUGCGUGAAUCUAAUCAAAGUUUCUCAUUAACUUCUGCGUUAGAUUCACGCACAAACUUGUCAUUUCCCUUUUCUU

Structure 1 Folding bases 1 To 100 of 10Sep29-17-55-46 Initial dG = -43.90

```
          10        20        30         40
    C     C    C       C               -  -     UCU
GAGA AGAGG AAAU GCAGGUU GUGCGUGAAUCUAAU CA AAGUU   \
|||| ||||| |||| ||||||| ||||||||||||||| || |||||
UUCU UUUCC UUUA UGUUCAA CACGCACUUAGAUUG GU UUCAA   C
    -     C    C       A               C  C     UUA
          90        80        70        60
```

SEQ16 UUAUAGUCUGACAUCUGGAAU

Gm05:9279518-9279737, 220nt, (+)

CAUCGAGAUUUUAUAGUCUGACAUCUGGAAUUUAAAUGUCUCAACAAGGGCGUUAGAUCCAACAACGUUCCACAAUGCUAAUGGGAGAAUUCAAAUUUGGGACCUCUCUCUCAUUUCUUCUCC
UUUCACGCUUUGAAACAAAUAAAGGACACCCGAGAUCCCCGUAGAAGCUCUUGUUGGAAAAUUUAGAUUUCAAAUGUCAGAUUAUAAAACAUGGGUG

Structure 1 Folding bases 1 To 220 of 10Sep29-17-57-12 Initial dG = -67.30

```
        10        20        30        40        50               60        70        80         90       100
    GAGA                C            GUC           GUUA-----    .-CAACAAC|  CCA   U  UAAU-       UUC    UU    C
CAUC    UUUUAUAGUCUGACAU UGGAAUUUAAAU   UCAACAAGGGC         GAUC         GUU   CAA GC     GGGAGAA   AAAU  GGGA C
||||    |||||||||||||||| ||||||||||||   |||||||||||         ||||         |||   ||| ||     |||||||   ||||  ||||
GUGG    AAAAUAUUAGACUGUA ACUUUAGAUUUA   GGUUGUUCUCG         CUAG         CAA   GUU CG     UCCUCUU   UUUA  CUCU U
    GUAC                A            AAA           AAGAUGCCC    \ -------^  A--   U  CACUU       C--    CU    C
.       210       200       190       180       170       160                     130       120         110
```

```
140       150
  AAAUAAA  A
         GG C
         ||
         CC A
  AG-----  C
```

SEQ17 ACUAUAGAAGUACUUGUGGAGC

Gm16:2916844-2917034, 191nt, (+)

GUAUUUAGCAAAUUGAUUACUAUAGAAGUACUUGUGGAGCUCUUGUGCAACUCUCCUUUCCAUCUUGGGGCAGUUUCUCAGAAUCAAUAUUCUUAUGAUGGGAAUCUGAAUUCACAGUUUUUG
UAACUGCAAAAACUUGUGAGGUUUGAUAGCUCCACAAGUAAAUCUAUAGUAAUUAAUUUGCUAAAUAC

Structure 3 Folding bases 1 To 191 of 10Sep29-18-02-46 Initial dG = -83.00

```
        10        20        30        40          50        60         70
                          AG             C   U  .-AAC  UCC        UU-|   CAGUUUCUC
GUAUUUAGCAAAUUGAUUACUAUAGA  UACUUGUGGAGCU UUG GC     UC   UUUCCAUC   GGGG         \
||||||||||||||||||||||||||  ||||||||||||| ||| ||     ||   ||||||||   ||||
CAUAAAUCGUUUAAUUAAUGAUAUCU  AUGAACACCUCGA AGU UG     AG   AAGGGUAG   UCUU         A
                          AA             U   U  \ ---  UCU        UAU^   AUAACUAAG
         180       170       160       150                  100        90
```

```
110        120
  AU     -        A
    UCACA GUUUUUGU A
    ||||| ||||||||
    AGUGU CAAAAACG C
  G-     U        U
    140       130
```

SEQ18 CCUCAUUCCAAACAUCAUCUAA

Gm09:16565935-16566025, 91nt, (-)

GUUUGCUAGAGGUGUUUGGGAUGAGAGAAUAGAAUUUUUUCAAAUGCUUGAAAGUGAUCUCUUCCCUCAUUCCAAACAUCAUCUAACACAC

Structure 1 Folding bases 1 To 91 of 10Sep29-18-04-30 Initial dG = -36.30

```
        10         20         30         40
  U  C    -               A   U   AUU       A
```

```
GU UG UAGA GGUGUUUGGGAUGAG GAA AGA   UUUUCAA U
|| || |||| ||||||||||||||| ||| |||   |||||||
CA AC AUCU CUACAAACCUUACUC CUU UCU   GAAAGUU G
  C  A    A               C   C   AGU       C
90        80        70        60        50
```

SEQ19 UGAAGAUUUGAAGAAUUUGGGA

Gm15:16900161-16900327, 167nt, (+)

UGAAGAUUUGAAGAAUUUGGGAGAAGGACGCCGUCAAGGUCGAGGGUUUCGUGACUACAGCUUCUGAAGCACGUCAUCUUCACAUAAAUAAGACGCGUUUCAGAAGUUGUAGUCACGAAACUC
UCGACCUAGACCGCAGCCCUUCUCCCAAAUUCUUCAAAUCUUCA

Structure 1 Folding bases 1 To 167 of 10Sep29-18-07-13 Initial dG = -119.10

```
        10        20         30        40        50        60        70        80
U                          AC-| C   A                                 A    AUCUUCA
 GAAGAUUUGAAGAAUUUGGGAGAAGG   GC GUC AGGUCGAGGGUUUCGUGACUACAGCUUCUGAAGC CGUC       C
 ||||||||||||||||||||||||||   || ||| |||||||||||||||||||||||||||||||||| ||||
 CUUCUAAACUUCUUAAACCCUCUUCC   CG CAG UCCAGCUCUCAAAGCACUGAUGUUGAAGACUUUG GCAG       A
A                          CGA^ C   A                                 C    AAUAAAU
     160       150       140       130       120       110       100        90
```

SEQ20 CAUCGUUGACGCUGACUGUACG

SEQ20(iso) CAUCGUUGACGCUGACUGU

Gm04:35428794-35428950, 157nt, (-)

UUUUCUAGUGGUCGCCGCAUCGUUGACGCUGACUGUACGUACUUCAUCUCCUUCAGAAUUCGGAAGGAUGCCAUAAACAAGCCACAUUAUGGUAUAGAAAUUUUUGGGAGUAAGGGUACGUCC
CGUCAGUGUCAAAGAUGUGGCGAAUACUAGAAAA

Structure 1 Folding bases 1 To 157 of 10Sep29-18-11-54 Initial dG = -75.00

```
        10        20        30        40        50        60
U         G          G           UGU          CAU     UC        |G
 UUUCUAGUG UCGCCGCAUC UUGACGCUGAC   ACGUACUU   CUCCU  AGAAUU--C G
 ||||||||| |||||||||| |||||||||||   ||||||||   |||||  ||||||   |
 AAAGAUCAU AGCGGUGUAG AACUGUGACUG   UGCAUGGG   GAGGG  UUUUAA   G A
A         A          A           CCC          AAU     U-       \ ^A
     150       140       130       120       110        100
```

```
        70        80
    G--          ACAA
       AUGCCAUAA    G
       |||||||||
       UAUGGUAUU    C
    AGA          ACAC
           90
```

SEQ21 CUGAAGGAUCGAUGUAGAAUGCU

Gm02:39825520-39825641, 122nt, (+)

GACCGUCUUAGAAUGCUCAGCAUUCUGCAAAGGUUCUUCAACAACCGUCGUAGAAUGUUGAGUUUUCUACAUCGAUCACAACUGAAGGAUCGAUGUAGAAUGCUGAGCAUUCUAAGACGGUC

Structure 1 Folding bases 1 To 122 of 10Sep29-18-13-11 Initial dG = -70.70

```
        10        20        30        40          50
                             AA          ACAAC---| UC       UGUU
GACCGUCUUAGAAUGCUCAGCAUUCUGCA  GGUUCUUCA        CG  GUAGAA    \
|||||||||||||||||||||||||||||  |||||||||        ||  ||||||
CUGGCAGAAUCUUACGAGUCGUAAGAUGU  CUAGGAAGU        GC  CAUCUU    G
                             AG          CAACACUA^ UA       UUGA
120       110       100        90        80        70
```

SEQ22 CAUCUGAAGGAUAGAACACAUA

Gm09:29816467-29816705, 239nt, (+)

CCUAAAAUUAUUUGUAAUAUUUGUGUUCUAUCCUUCAAAUGACUUCCUCAAUAAUAAACUUCGGACUUUGGUAUACAAAGAAAAAACUAUAAACCAAAUUUAUGGUAAAGUCCGAAGUUACCA
AAUAAUGUUUACCAUAAAUUUGGUUUAUAGUUUUUUCUUUGUAUACCAAAGUCCGAAAUUUAUUAUUGAGGAAGUCAUCUGAAGGAUAGAACACAUAUAUUACAAAUAAUUUUAGG

Structure 1 Folding bases 1 To 239 of 10Sep29-18-15-19 Initial dG = -153.80

```
        10        20        30        40        50        60        70        80        90        100       110
                    U                A                    C                                                  GUCCGAAGUU
CCUAAAAUUAUUUGUAAUAU UGUGUUCUAUCCUUCA AUGACUUCCUCAAUAAUAAA UUCGGACUUUGGUAUACAAAGAAAAAACUAUAAACCAAAUUUAUGGUAAA          \
|||||||||||||||||||| |||||||||||||||| |||||||||||||||||||| ||||||||||||||||||||||||||||||||||||||||||||||||||
GGAUUUUAAUAAACAUUAUA ACACAAGAUAGGAAGU UACUGAAGGAGUUAUUAUUU AAGCCUGAAACCAUAUGUUUCUUUUUUGAUAUUUGGUUUAAAUACCAUUU          A
                    U                C                    A                                                  GUAAUAAACC
       230       220       210       200       190       180       170       160       150       140       130
```

SEQ23 AGUUUCGUGACUACAACUUCUGAA

Gm15:16900193-16900294, 102nt, (-)

GUCUAGGUCGAGAGUUUCGUGACUACAACUUCUGAAACGCGUCUUAUUUAUGUGAAGAUGACGUGCUUCAGAAGCUGUAGUCACGAAACCCUCGACCUUGAC

Structure 1 Folding bases 1 To 102 of 10Sep29-18-22-02 Initial dG = -63.30

```
        10        20        30        40        50
   U        A              A        A      UUA   A
GUC AGGUCGAG GUUUCGUGACUACA CUUCUGAA CGCGUC   UUU U
||| |||||||| |||||||||||||| |||||||| ||||||   |||
CAG UCCAGCUC CAAAGCACUGAUGU GAAGACUU GUGCAG   AAG G
   U        C              C        C      UAG   U
100        90        80        70        60
```

SEQ24 AUGAAAAUCAUUCAUUAUGAUAUC

Gm16:28536014-28536181, 168nt, (-)

CACGUCAUCACAGACAUCAUAAUGAAUGAUUUUCAUGUUUAUAUUUAAAUUAGUUGUUAUAUGUUUGUCAUUUGAAUUUGACACUUAAUUUGUUCAAAUUUAUACUUACAUACAACAAAUUUA
AACAGAGACAUGAAAAUCAUUCAUUAUGAUAUCCGUGAUGACGUG

Structure 1 Folding bases 1 To 168 of 10Sep29-18-24-25 Initial dG = -73.40

```
        10        20        30        40        50        60        70        80
           A  C                         AUA        A     UAU    UU  CAUU         C  UUA
CACGUCAUCAC GA AUCAUAAUGAAUGAUUUUCAUGUUU   UUUAAAUU GUUGU   AUGU  GU    UGAAUUUGA AC   \
||||||||||| || |||||||||||||||||||||||||   |||||||| |||||   ||||  ||    ||||||||| ||
GUGCAGUAGUG CU UAGUAUUACUUACUAAAAGUACAGA   AAAUUUAA CAACA   UACA  CA    AUUUAAACU UG   A
           C  A                         GAC        A     ---    UU  U---         -  UUU
      160       150       140       130       120          110          100         90
```

SEQ25a GAAAAUGAAUGAUGAGGAUGGGGA

Gm11:7787358-7787494, 137nt, (+)

GUAACUCUUUUAGAGUUACUUCUCAUCCUCACCAUUCAUUUUUUUAAGAUCUAAUGGUUAAAAUUAGUUACUUAUUACAACCAUUAGAUUUUAAGAAAAUGAAUGAUGAGGAUGGGGAGUAAC
UCUAAAAAAGUUAC

Structure 1 Folding bases 1 To 137 of 10Sep29-18-26-31 Initial dG = -77.60

```
        10        20        30        40        50        60
      C                        C                           AA   U  U
GUAACU UUUUAGAGUUACUUCUCAUCCUCA CAUUCAUUUUUUUAAGAUCUAAUGGUU  AAU AG \
|||||| |||||||||||||||||||||||| |||||||||||||||||||||||||||  ||| ||
CAUUGA AAAAUCUCAAUGAGGGGUAGGAGU GUAAGUAAAAGAAUUUUAGAUUACCAA  UUA UC U
```

```
      A                        A                           CA   U  A
     130       120       110       100        90        80        70
```

SEQ25b GAAAAUGAAUGAUGAGGAUGGGGA

Gm11:7821070-7821206, 137nt, (-)

GUAACUCUUUUAGAGUUACUCCUCAUCCUCACCAUUCAUUUUCUUAAGAUGUAAUGGUUAAAAUUAGUUACUUAUUACAACCAUUAGAUUUUAAGAAAAUGAAUGAUGAGGAUGGGGAGUAACUCUAAAAAAGUUAC

```
Structure 1 Folding bases 1 To 137 of 10Sep29-18-27-35     Initial dG = -79.00

        10        20        30        40        50        60
      C                        C                  G        AA   U  U
GUAACU UUUUAGAGUUACUCCUCAUCCUCA CAUUCAUUUUCUUAAGAU UAAUGGUU  AAU AG \
|||||| |||||||||||||||||||||||| |||||||||||||||||| ||||||||  ||| ||
CAUUGA AAAAUCUCAAUGAGGGGUAGGAGU GUAAGUAAAAGAAUUUUA AUUACCAA  UUA UC U
      A                        A                  G        CA   U  A
     130       120       110       100        90        80        70
```

Identification of homologues miRNAs of other plant species

gma-MIR170 UAUUGGCCUGGUUCACUCAGA

Gm02:4014001-4014153, 153nt, (-)

AUGAAGUAGUUAUUGUGAUAUUGGCCUGGUUCACUCAGACAUGUAUACCACGGCACGGUUGUGUCCUUGCUAGAAAGAUUUAAAACAAAAUACAUGUUGUGAUUGUGGUUUUGGGUUUGAUUGAGCCGUGCCAAUAUCUCAGUGCUAUUUCAU

```
Structure 1 Folding bases 1 To 153 of 10Sep29-18-30-26     Initial dG = -61.30

        10        20        30        40        50          60        70
         U     U         C       C      AUGUAU       -   GG -     CC   C--|    A
AUGAAGUAG UAUUG GAUAUUGGC UGGUUCA UCAGAC      ACCACGG CAC  U UGUGU  UUG   UAGA A
||||||||| ||||| ||||||||| ||||||| ||||||      ||||||| |||  | |||||  |||   ||||
UACUUUAUC GUGAC CUAUAACCG GCCGAGU AGUUUG      UGGUGUU GUG  G ACAUA  AAC   AUUU G
```

```
         -     U         U       U      GGUUU-       A   UU U     A-   AAA^   A
 150        140       130       120       110        100        90         80
```

gma-MIR395a CUGAAGUGUUUGGGGGAACUC
Gm01:4818581-4818690, 110nt, (+)
UCAGGUUUUCCCUAGAGUUCCCCUGAACGCUUCAUUAAAGGGCUUUAUUAUCAUAUAGUCCCAAGUUAGUCCAUACUGAAGUGUUUGGGGGAACUCCCGGUGAUACUUGA

Structure 2 Folding bases 1 To 110 of 10Sep29-18-31-25 Initial dG = -47.50

```
        10        20        30        40        50
      U UC  UA         UG         U  AA|    UUAUUAUCAUA
UCAGGU U  CC  GAGUUCCCC  AACGCUUCA UA  GGGCU           \
|||||| |  ||  |||||||||  ||||||||| ||  |||||
AGUUCA A  GG  CUCAAGGGG  UUGUGAAGU AU  CCUGA           U
      U GU  CC         GU         C  A-^    UUGAACCCUGA
        100        90        80         70        60
```

gma-MIR395b CUGAAGUGUUUGGGGGAACUC
Gm02:1723449-1723543, 95nt, (-)
UCCCUAGAGUUUCCCUGAACACUUCAUUAAAGGGCUUUAUUGAAAUAUAAUCCAAAGUUAGUCUAUACUGAAGUGUUUGGGGGAACUCCUGGUGA

Structure 1 Folding bases 1 To 95 of 10Sep29-18-34-15 Initial dG = -38.30

```
         10        20        30         40
  -| UA         UG         U  AA     UUA-   AAAU
UC CC  GAGUUUCCC  AACACUUCA UA  GGGCU    UUG    A
|| ||  |||||||||  ||||||||| ||  |||||    |||
AG GG  CUCAAGGGG  UUGUGAAGU AU  UCUGA    AAC    U
  U^ UC         GU         C  A-     UUGA   CUAA
    90        80        70         60        50
```

gma-MIR395c CUGAAGUGUUUGGGGGAACUC
Gm08:40840226-40840312, 87nt, (+)
CCUAGAGUUCCCCUUAAUGCUUCAUUGAGGAUUCUGUUUAGGUCCAAUUUAACUAGUCCCUACUGAAGUGUUUGGGGGAACUCCGGG

Structure 1 Folding bases 1 To 87 of 10Sep29-18-35-47 Initial dG = -38.30

```
        10        20        30        40
```

```
| UA          U         U  A     CU   U  GU
CC  GAGUUCCCCU AAUGCUUCA UG GGAUU  GUU AG  C
||  |||||||||| ||||||||| || |||||  ||| ||
GG  CUCAAGGGGG UUGUGAAGU AU CCUGA  CAA UU  C
^ GC          U         C  C     U-   U  AA
      80        70        60          50
```

gma-MIR397a UCAUUGAGUGCAGCGUUGAUG

Gm08:4639046-4639153, 108nt, (-)

AGAGAAACAUCAUUGAGUGCAGCGUUGAUGAAGUUUCACUCUCAUCUCAGGUAGAUGCUUAAUUUAUAGUGUUAUUGUCAUCGACACUGCACUCAAUCAUGUUUUUCU

Structure 1 Folding bases 1 To 108 of 10Sep29-18-37-16   Initial dG = -46.10

```
        10        20        30         40
          C           C        -   UU    CU--------|    CA
AGAGAAACAU AUUGAGUGCAG GUUGAUGA AGU  CACU          CAUCU  \
|||||||||| ||||||||||| |||||||| |||  ||||          |||||
UCUUUUUGUA UAACUCACGUC CAGCUACU UUA  GUGA          GUAGA  G
          C           A        G   UU    UAUUUAAUUC^    UG
      100        90        80        70        60
```

gma-MIR397b UCAUUGAGUGCAGCGUUGAUG

Gm13:34382999-34383120, 122nt, (-)

GGAGAAACAUCAUUGAGUGCAGCGUUGAUGAAGUCCUAAAUAGUAAAUAGCAACUACCCCUCGUGUUUCUCAGGUAGAUGCUUAAAUGGUUUUAUUGACGCUGCACUCAAUCAUGUUUUUUU

Structure 1 Folding bases 1 To 122 of 10Sep29-18-40-16   Initial dG = -51.10

```
        10        20        30        40        50        60
          C              UG      U  UAAAUAGUAAAU    A     CCUCGU
GGAGAAACAU AUUGAGUGCAGCGU  AUGAAG CC            AGCA CUACC      G
|||||||||| ||||||||||||||  |||||| ||            |||| |||||
UUUUUUUGUA UAACUCACGUCGCA  UAUUUU GG            UCGU GAUGG      U
          C              GU      -  UAAAU-------    A     ACUCUU
120       110       100        90                80        70
```

gma-MIR408a AUGCACUGCCUCUUCCCUGGC

gma-MIR408a(iso) UGCACUGCCUCUUCCCUGGC

Gm02:837416-837548, 133nt, (+)

GAGACAGGACAAAGCAGGGGAACAGGCAGAGCAUGGAUGGAGCUAUCAACACAAUAUUGUCAAGAAACUGAGAGUGAGAGGAGAAAUAUGUUGUGGUUCUGCUCAUGCACUGCCUCUUCCCUG
GCUCUGUCUC

```
Structure 1 Folding bases 1 To 133 of 10Sep29-18-42-13     Initial dG = -58.30

        10        20        30        40        50         60        70
       GACAA    A      C      A     GA         U     CAA    G-   AAGAA   UG
GAGACAG     AGC GGGGAA AGGCAG GCAUG  UGGAGCUA CAACA   UAUU  UC     AC  \
|||||||     ||| |||||| |||||| |||||  |||||||| |||||   ||||  ||     ||
CUCUGUC     UCG UCCCUU UCCGUC CGUAC  GUCUUGGU GUUGU   AUAA  AG     UG  A
       -----   G      C      A     UC         -     ---    AG   GAGAG   AG
 130            120       110       100         90            80
```

gma-MIR408b-5p CUGGGAACAGGCAGGGCACG
gma-MIR408b-3p AUGCACUGCCUCUUCCCUGGC
Gm03:44626696-44626827, 132nt, (-)
GACAAAGGCUGGGAACAGGCAGGGCACGAAUGGAGCUAUCAACAGAAAAUGGUAAAGUGAGAAUGAAAGGAGAGAGAGAGAGAGAGAGAUCUGUUGUGGCUACGCUCAUGCACUGCCUCUUCCCU
GGCUCUGUC

```
Structure 1 Folding bases 1 To 132 of 10Sep29-18-43-09     InitialdG = -45.36

        10         20        30        40        50        60
    AA     -     C      G   C  A  G     U       AAAUGGUAAAGUGAGAAUGA
GACA  GGCU GGGAA AGGCAG GCA GA UG AGCUA CAACAGA                    \
||||  |||| ||||| |||||| ||| || || ||||| |||||||
CUGU  UCGG CCCUU UCCGUC CGU CU GC UCGGU GUUGUCU                    A
    C-    U     C      A   A  C  A     -       AGAGAGAGAGAGAGAGAGGA
130       120       110       100        90        80        70
```

gma-MIR408c AUGCACUGCCUCUUCCCUGGC
gma-MIR408c(iso) UGCACUGCCUCUUCCCUGGC
Gm10:36557005-36557130, 126nt, (-)
GACAAAGCAGGGGAACAGGCAGAGCAUGGAUGGAGCUAUCAACACAAUAUUGUCAAGAAAGUGAGAAAGUGAGAGGAGAAAUCUGUUGUGGUUCUGCUCAUGCACUGCCUCUUCCCUGGCUUU
GUC

```
Structure 1 Folding bases 1 To 126 of 10Sep29-18-44-58     Initial dG = -59.60

        10        20        30        40          50
        A      C      A     GA         U    .-A| AUA  G   AG
GACAAAGC GGGGAA AGGCAG GCAUG  UGGAGCUA CAAC   CA   UU UCA  A
|||||||| |||||| |||||| |||||  |||||||| ||||   ||   || |||
CUGUUUCG UCCCUU UCCGUC CGUAC  GUCUUGGU GUUG   GU   AA AGU  A
        G      C      A     UC         -    \ -^ GA-  G   GA
    120       110       100        90         70         60


                                              AGA   G
```

```
                                          GGA A
                                          |||
                                          UCU A
                                       ---   A
                                             80
```

gma-MIR2218a-5p GGAGAUGGGAGGGUCGGUAAAG
gma-MIR2218a-5p(iso) GGAGAUGGGAGGGUCGGUAA
gma-MIR2218a-3p UUGCCGAUUCCACCCAUUCCUA
Gm10:48574017-48574137, 121nt, (-)
GAGCUUGAGGAAGUGAUGGGAGAUGGGAGGGUCGGUAAAGGAUAACAGCGUCUCUAUGAUUAAUUGUUGUGUUGUUUAUUCUUUUGCCGAUUCCACCCAUUCCUAUGAUUUUCUUUGGUUC

Structure 1 Folding bases 1 To 121 of 10Sep29-18-46-18 Initial dG = -52.00

```
        10        20        30          40          50
     U        G      G     A  -             U--|      UCUCU  GA
GAGCU GAGGAAGU AUGGGA AUGGG GG GUCGGUAAAGGA    AACAGCG     AU  \
||||| |||||||| |||||| ||||| || ||||||||||||    |||||||     ||
CUUGG UUCUUUUA UAUCCU UACCC CC UAGCCGUUUUCU    UUGUUGU     UA  U
     U        G      -     A  U             UAU^      GUUGU  AU
         110        100        90        80        70
```

gma-MIR2218b-5p GGAGAUGGGAGGGUCGGUAA
gma-MIR2218b-3p UUGCCGAUUCCACCCAUUCCUA
Gm20:35349741-35349881, 141nt, (+)
GAGCUUGAGGAAGUGAUGGGAGAUGGGAGGGUCGGUAAAGAAUAUAUCUGAGACUCGACUCAAUCUCGAUCUCUCUCAGUGUUGUGUUGUUUUGUUUAUCCUUUUGCCGAUUCCACCCAUUCC
UAUGAUUUCCUUCGGUUC

Structure 2 Folding bases 1 To 141 of 10Sep29-18-46-46 Initial dG = -53.80

```
        10        20        30          40        50        60          70
     U        G      G     A  -           AAU|  UC      UC   U    -  C  UC
GAGCU GAGGAAGU AUGGGA AUGGG GG GUCGGUAAAG   AUA  UGAGAC  GAC CAAU CU GA  U
||||| |||||||| |||||| ||||| || ||||||||||   |||  ||||||  ||| |||| || ||
CUUGG UUCCUUUA UAUCCU UACCC CC UAGCCGUUUU   UAU  GUUUUG  UUG GUUG GA CU  C
     C        G      -     A  U           CC-^  UU      --   U    U  -  CU
 .       130        120       110       100         90          80
```

gma-MIR3522a AGACCAAAUGAGCAGCUGA

gma-MIR3522a(iso1) GAGACCAAAUGAGCAGCUGA
gma-MIR3522a(iso2) UGAGACCAAAUGAGCAGCUGA
gma-MIR3522a(iso3) UCGUCCUGAGACCAAAUGAGC
Gm15:4318787-4318873, 87nt, (+)
AGGAUCGUCCUGAGACCAAAUGAGCAGCUGACCACAUGAUGCAGCUAUGUUUGCUAUUCAGCUGCUCAUCUGUUCUCAGGUCGCCCU

Structure 1 Folding bases 1 To 87 of 10Sep29-18-49-35 Initial dG = -40.20

```
        10        20        30        40
   AU   U       C  A            CCACAUGAU   G
AGG  CG CCUGAGA CA AUGAGCAGCUGA         GCA C
|||  || ||||||| || ||||||||||||         |||
UCC  GC GGACUCU GU UACUCGUCGACU         UGU U
   C-   U       U  C            UAUCGUU--   A
       80        70        60          50
```

Identification of conserved soybean miRNAs

gma-MIR156d UUGACAGAAGAUAGAGAGCAC
Gm08:3891365-3891489, 125nt, (+)
AAGGUUGUUGACAGAAGAUAGAGAGCACAGAUGAUGAUAUGCAUAUUAUAUAAUAUAUAGCAGGGAACUCAUGAUGAAUUGUGCAUCUUACUCCUUUGUGCUCUCUAUACUUCUGUCAUCACC
UU

Structure 1 Folding bases 1 To 125 of 10Sep29-10-05-37 Initial dG = -50.60

```
        10         20        30         40          50
    U  U         -              U  -   U       .-U|     A
AAGG UG UGACAGAAG AUAGAGAGCACAGA GA UGA AUGCAUA   UAUAU \
|||| || ||||||||| |||||||||||||| || ||| |||||||   |||||
UUCC AC ACUGUCUUC UAUCUCUCGUGUUU CU AUU UACGUGU   AUAUA A
    -  U         A              C  C   C       \ -^     U
    120       110       100        90        80
```

```
                                                      GC--------  G
                                                                AG G
                                                                ||
                                                                UC A
                                                      UAAGUAGUAC  A
                                                              70
```

gma-MIR156g(iso) UUGACAGAAGAUAGAGAGCAC

Gm19:8895390-8895493, 104nt, (+)

GAGAAGUACAUUGACAGAAGAUAGAGAGCACAAAACUGCUCACACACAAAAGCUUUUUUGGGUAUGAUCACCUGUGCUCUCUAUCUUCUGUCAACAAACUGUUC

```
Structure 1 Folding bases 1 To 104 of 10Sep29-10-13-50 Initial dG = -45.30
        10        20        30        40        50
|  A   ACA                      AAAC   C   C  A     G
GAG AGU   UUGACAGAAGAUAGAGAGCACA    UG UCA AC CAAAA C
||| |||   ||||||||||||||||||||||    || ||| || |||||
CUU UCA   AACUGUCUUCUAUCUCUCGUGU    AC AGU UG GUUUU U
^  G   AAC                      CC--  U   A  G     U
  100       90        80          70        60
```

gma-MIR156h UUGACAGAAGAUAGAGAGCAC

Gm02:7812526-7812628, 103nt, (+)

GGUGAUGCUGUUGACAGAAGAUAGAGAGCACAGAUGAUGAAAUGCAAGAAAGGAAAUGGCAUCUUACUCUUUUGUGCUCUCUAGUCUUCUUGUCAUCAUCAUU

```
Structure 1 Folding bases 1 To 103 of 10Sep29-09-32-40 Initial dG = -41.80

        10         20         30         40        50
|      CUGU     -      -            U  -   A    AAGAAA
GGUGAUG    UGACA GAAGAU AGAGAGCACAGA GA UGA AUGC      \
|||||||    ||||| |||||| |||||||||||| || ||| ||||
UUACUAC    ACUGU CUUCUG UCUCUCGUGUUU CU AUU UACG      G
^      U---     U      A            U  C   C    GUAAAG
 100           90        80        70        60
```

gma-MIR156i UUGACAGAAGAUAGAGAGCAC

Gm05:38621690-38621813, 124nt, (+)

AAGGUUGUUGACAGAAGAUAGAGAGCACAGAUGAUGAUAUGCAUAUUAUAUAAAAAGCAGCUAGGGAACUCAUGAAUUGUGCAUCUCACUCCUUUGUGCUCUCUAUACUUCUGUCAUCACCCU
U

```
Structure 1 Folding bases 1 To 124 of 10Sep29-09-41-56 Initial dG = -53.40

        10         20        30         40        50        60
    U  U         -              U  -|  U       UUAUAUAAAAAG  GCU  G
AAGG UG UGACAGAAG AUAGAGAGCACAGA GA UGA AUGCAUA            CA   AG G
|||| || ||||||||| |||||||||||||| || ||| |||||||            ||   ||
UUCC AC ACUGUCUUC UAUCUCUCGUGUUU CU ACU UACGUGU            GU   UC A
    C  U         A              C  C^  C       UAA---------  AC-  A
  120       110       100        90        80                  70
```

gma-MIR156j UUGACAGAAGAGAGUGAGCAC
Gm06:4699149-699240, 92nt, (+)
GAAAUUGACAGAAGAGAGUGAGCACACAGAGGCACUUGGUAUAGUAUAUAUACUAUUGCUUUUGCGUGCUCACUUCUCUUUCUGUCAACUUC

```
Structure 1 Folding bases 1 To 92 of 10Sep29-09-45-47 Initial dG = -51.00
        10          20        30        40
   A       -     -|        A        CU       GU
GAA UUGACAG AAGAG AGUGAGCAC CAGAGGCA  UGGUAUA  \
||| ||||||| ||||| ||||||||| ||||||||  |||||||
CUU AACUGUC UUCUC UCACUCGUG GUUUUCGU  AUCAUAU  A
   C       U     U^        C        U-       AU
 90        80        70        60         50
```

gma-MIR156k UUGACAGAAGAUAGAGAGCAC
Gm07:9347139-9347259, 121nt, (+)
GGUAAGGUUGUUGACAGAAGAUAGAGAGCACAGAUGAUGAUAUGCACAUAUACAUGGAACAGGAAUUUAAGCAAUUGCAUCUCACUCCUUUGUGCUCUCUAAGCUUCUGUCAUCCACCUCC

```
Structure 1 Folding bases 1 To 121 of 10Sep29-09-47-04 Initial dG = -45.60

        10        20         30         40        50        60
| UA    UGU         A-             U  -   U     CAUAUACAUGGAAC
GG  AGGU   UGACAGAAG  UAGAGAGCACAGA GA UGA AUGCA              \
||  ||||   |||||||||  ||||||||||||| || ||| |||||
CC  UCCA   ACUGUCUUC  AUCUCUCGUGUUU CU ACU UACGU              A
^ --    CCU         GA             C  C   C     UAACGAAUUUAAGG
 .         110       100        90        80        70
```

gma-MIR156l UUGACAGAAGAUAGAGAGCAC
Gm09:37843750-37843864, 115nt, (-)
AGGGUUGUUGACAGAAGAUAGAGAGCACAGAUAGUGAUAUGCAUAAAAAUAUGGAACGGGAAAGCAAUUGCAUCUCACUCCUUUGUGCUCUCUAGGCUUCUGUCAUCCACACCUU

```
Structure 1 Folding bases 1 To 115 of 10Sep29-10-08-02 Initial dG = -49.30
          10        20        30          40        50
```

```
     -  U-          A-              U-|    U     UAAAAAUAUGGA
AGGGU UG  UGACAGAAG  UAGAGAGCACAGA  AGUGA AUGCA            \
||||| ||  |||||||||  |||||||||||||  ||||| |||||
UUCCA AC  ACUGUCUUC  AUCUCUCGUGUUU  UCACU UACGU            A
     C  CU          GG              CC^    C     UAACGAAAGGGC
   110       100        90         80         70         60
```

gma-MIR156m UUGACAGAAGAGAGUGAGCAC
Gm14:10664512-10664600, 89nt, (-)
AAAUUGACAGAAGAGAGUGAGCACAAAGAGGCACUUGAUAUAAUUCUAUAUCACUGCUUUUGUGUGCUCACCACUCUUUCUGUCGGUUU

```
Structure 1 Folding bases 1 To 89 of 10Sep29-10-09-55 Initial dG = -44.90
        10          20        30        40
          -     A-|        A       CU       A
AAAUUGACAG AAGAG  GUGAGCACA AGAGGCA  UGAUAUA U
|||||||||| |||||  ||||||||| |||||||  |||||||
UUUGGCUGUC UUCUC  CACUCGUGU UUUUCGU  ACUAUAU U
          U     AC^        G       C-       C
        80        70        60         50
```

gma-MIR156n UUGACAGAAGAGAGUGAGCAC
Gm17:37759446-37759535, 90nt, (+)
AAAUUGACAGAAGAGAGUGAGCACAAAGAGGCACUUGAUAUAAAUCUAUAUCACUGCUUUUGUGUGCUCACUACUCUUUCUGUCGGUUUU

```
Structure 1 Folding bases 1 To 90 of 10Sep29-10-11-28 Initial dG = -47.80
         10          20        30        40
-          -     -|         A       CU       A
 AAAUUGACAG AAGAG AGUGAGCACA AGAGGCA  UGAUAUA A
 |||||||||| ||||| |||||||||| |||||||  |||||||
 UUUGGCUGUC UUCUC UCACUCGUGU UUUUCGU  ACUAUAU U
U          U     A^         G       C-       C
.        80        70        60         50
```

**gma-MIR159a-5p**	GAGCUCCUUGAAGUCCAAUUG	Gm09	37672410	37672586
**gma-MIR159a-5p(iso)**	GAGCUCCUUGAAGUCCAAUU	Gm09	37672410	37672586
**gma-MIR159a-3p**	UUUGGAUUGAAGGGAGCUCUA	Gm09	37672410	37672586
**gma-MIR159a-3p(iso1)**	UUUGGAUUGAAGGGAGCUCU	Gm09	37672410	37672586
**gma-MIR159a-3p(iso2)**	UUGGAUUGAAGGGAGCUCUA	Gm09	37672410	37672586
**gma-MIR159a-3p(iso3)**	UGGAUUGAAGGGAGCUCUA	Gm09	37672410	37672586

Gm09:37672410-37672586, 177nt, (+)
GUGGAGCUCCUUGAAGUCCAAUUGAGGAUCUUACUGGGUGAAUUGAGCUGCUUAGCUAUGGAUCCCACAGUUCUACCCAUCAAUAAGUGCUUUUGUGGUAGUCUUGUGGCUUCCAUAUCUGGG
GAGCUUCAUUUGCCUUUAUAGUAUUAACCUUCUUUGGAUUGAAGGGAGCUCUAC

Structure 1 Folding bases 1 To 177 of 10Sep29-10-28-08 Initial dG = -85.40

```
        10        20        30            40        50        60        70         80
            GA       UU    AUCU    ----   UG   U     G     C      UC      UU     -| UCAAUA
GUGGAGCUCCUU  AGUCCAA  GAGG    UACU    GGG  AAU GAGCU CUUAG UAUGGA  CCACAG  CUACC CA      A
||||||||||||  |||||||  ||||    ||||    |||  ||| ||||| ||||| ||||||  ||||||  ||||| ||
CAUCUCGAGGGA  UUAGGUU  UUCC    AUGA    UCC  UUA UUCGA GGGUC AUACCU  GGUGUU  GAUGG GU      G
            AG       UC    AAUU    UAUU   GU   C     G     U      UC      CU     U^ UUUCGU
     170       160       150       140       130       120       110       100        90
```

**gma-MIR159b-5p**	GAGUUCCCUGCACUCCAAGUC	Gm07	5386107	5386292
**gma-MIR159b-3p**	AUUGGAGUGAAGGGAGCUCCA	Gm07	5386107	5386292

Gm07:5386107-5386292, 186nt,(-)
AAACCCAACUUGGAGUUCCCUGCACUCCAAGUCUGAAAGGAUAUGAUGGUAAACCUCUACUGCUAGUUCAUGGAUACCUCUGACUUCUUAACAACAUGCGUUCGAAGUCAAGGGUUUGCAUGC
CCUGGGAGAUGAGUUUACCUUGAUCUUUUGGUAUUGGAGUGAAGGGAGCUCCAGAGGGUAUUC
Structure 1 Folding bases 1 To 186 of 10Sep29-10-15-16 Initial dG = -83.80

```
          10        20        30        40        50        60        70        80        90
AA--|   AAC            G        GU  G       AUGAU       C  UA  G    UU    GAU   UC       UU   AAC
    ACCC   UUGGAGUUCCCU CACUCCAA  CU AAAGGAU     GGUAAAC UC  CU CUAG  CAUG   ACC  UGACUUC  AAC   \
    ||||   |||||||||||| ||||||||  || |||||||     ||||||| ||  || ||||  ||||   |||  |||||||  |||
    UGGG   GACCUCGAGGGA GUGAGGUU  GG UUUUCUA     CCAUUUG AG  GA GGUC  GUAC   UGG  ACUGAAG  UUG   A
CUUA^   A--            A        AU  -       GUU--       -  UA  G    CC    GUU   GA       C-   CGU
    180         170       160        150         140        130       120       110        100
```

**gma-MIR159e-5p**	GAGCUCCUUGAAGUCCAAUU	Gm07	9524917	9525127
**gma-MIR159e-3p**	UUUGGAUUGAAGGGAGCUCUA	Gm07	9524917	9525127
**gma-MIR159e-3p(iso1)**	UUUGGAUUGAAGGGAGCUCU	Gm07	9524917	9525127
**gma-MIR159e-3p(iso2)**	UUUGGAUUGAAGGGAGCUC	Gm07	9524917	9525127
**gma-MIR159e-3p(iso3)**	UUGGAUUGAAGGGAGCUCUA	Gm07	9524917	9525127

gma-MIR159e-3p(iso4) UGGAUUGAAGGGAGCUCUA Gm07 9524917 9525127

Gm07:9524917-9525127, 211nt, (-)

CAAAGGGGGUUAUGGAGUGGAGCUCCUUGAAGUCCAAUUGAGGAUCUUACUGGGUGGAUUGAGCUGCUUAGCUAUGGAUCCCACAGUUCUACCCAUCAUUAAGUGCUUUUGUGGUAGUCUUGU
GGCUUCCAUAUCUGGGGAGCUUCAUUUGCCUUUAUAGUAUUAUCCUUCUUUGGAUUGAAGGGAGCUCUACACCCUUCUCCCCUUUUGU

Structure 1 Folding bases 1 To 211 of 10Sep29-10-19-50 Initial dG = -108.00

```
         10         20        30        40        50            60        70        80        90        100
-         UUAU-  A            GA       UU      CU    ----   UG   U     G     C      UC      UU     -| UCAUUA
 CAAAGGGGG     GG GUGGAGCUCCUU  AGUCCAA  GAGGAU  UACU    GGG  GAU GAGCU CUUAG UAUGGA  CCACAG  CUACC CA      A
 |||||||||     || ||||||||||||  |||||||  ||||||  ||||    |||  ||| ||||| ||||| ||||||  ||||||  ||||| ||
 GUUUUCCCC     CC CAUCUCGAGGGA  UUAGGUU  UUCCUA  AUGA    UCC  UUA UUCGA GGGUC AUACCU  GGUGUU  GAUGG GU      G
U         UCUUC  A            AG       UC      UU    UAUU   GU   C     G     U      UC      CU     U^ UUUCGU
 .       200       190       180       170       160       150       140       130       120       110
```

gma-MIR159f-5p GAGUUCCCUGCACUCCAAGUC

gma-MIR159f-3p AUUGGAGUGAAGGGAGCUCCA

Gm16:2794128-2794307, 180nt, (-)

ACCCAAGUUGGAGUUCCCUGCACUCCAAGUCUGAAAGGAUAUGAUGGUAAACCUCUGCUGCUAGUUCAUGGAUACCUCUGGCCUCGUAACAACAUGCGUUCGAAGUCAAGGGUUUGCAUGACC
UGGGAGAUGAGUUUACCUUGACCUUUUGGUAUUGGAGUGAAGGGAGCUCCAGAGGGU

Structure 1 Folding bases 1 To 180 of 10Sep29-10-34-39 Initial dG = -85.60

```
        10        20        30        40        50        60        70        80        90
|   AAG            G        GU UG     AUAUGAU       C  UG  G    U     GAU   UC    C   U   AAC
ACCC   UUGGAGUUCCCU CACUCCAA  C  AAAGG       GGUAAAC UC  CU CUAG UCAUG   ACC  UGGC UCG AAC   \
||||   |||||||||||| ||||||||  |  |||||       ||||||| ||  || |||| |||||   |||  |||| ||| |||
UGGG   GACCUCGAGGGA GUGAGGUU  G  UUUCC       CCAUUUG AG  GA GGUC AGUAC   UGG  ACUG AGC UUG   A
^   A--            A        AU GU     AGUU---       -  UA  G    C     GUU   GA    A   -   CGU
.         170       160       150          140        130       120       110        100
```

gma-MIR162a UCGAUAAACCUCUGCAUCCAG

Gm06:20176238-20176339, 102nt,(-)

GUGAAGUCACUGGAUGCAGCGGUUCAUCGAUCUCUUCCUGAAUCGUUGUUUAACAUCAGAACCAUGAAUCGAUCGAUAAACCUCUGCAUCCAGCGCUCACUC

Structure 1 Folding bases 1 To 102 of 10Sep29-10-38-42 Initial dG = -43.20

```
          10        20        30        40         50
--|   A  CA          C    C       UC   C  AA  GU-  UU
  GUGA GU  CUGGAUGCAG GGUU AUCGAUC  UUC UG  UC   UG  \
  |||| ||  |||||||||| |||| |||||||  ||| ||  ||   ||
  CACU CG  GACCUACGUC CCAA UAGCUAG  AAG AC  AG   AC  U
CU^   -  C-          U    A       CU   U  CA  ACU  AA
100          90        80        70        60
```

gma-MIR162b UCGAUAAACCUCUGCAUCCAG
Gm05:7692594-7692698, 105nt, (-)
GGUGAAGUCACUGGAGGCAGCGGUUCAUCGAUCUCUUCCUGAAUUUGGUUGUGGAAGAACACAAAGCAAGAAUCGGUCGAUAAACCUCUGCAUCCAGCGCUCACU

Structure 1 Folding bases 1 To 105 of 10Sep29-10-36-20 Initial dG = -41.80

```
        10        20        30        40        50
|    A  CA     G    C    C       UC   C  AAUUUGG     GA
GGUGA GU  CUGGA GCAG GGUU AUCGAUC  UUC UG       UUGUG  A
||||| ||  ||||| |||| |||| |||||||  ||| ||       |||||
UCACU CG  GACCU CGUC CCAA UAGCUGG  AAG AC       AACAC  G
^    -  C-     A    U    A       CU   A  GA-----     AA
    100         90        80        70             60
```

gma-MIR162c UCGAUAAACCUCUGCAUCCAG
Gm17:10181489-10181607, 119nt, (+)
GAGAUGAGGUGAAGUCACUGGAGGCAGCGGUUCAUCGAUCUCUUCCUGAAUUUGGUUGUGGAAGAACACAAAGCAAGAAUCGGUCGAUAAACCUCUGCAUCCAGCGCUCACUUUGCCUC

Structure 1 Folding bases 1 To 119 of 10Sep29-10-40-19 Initial dG = -48.40

```
        10        20        30        40        50        60
|  AU        A  CA     G    C    C       UC   C  AAUUUGG     GA
GAG  GAGGUGA GU  CUGGA GCAG GGUU AUCGAUC  UUC UG       UUGUG  A
|||  ||||||| ||  ||||| |||| |||| |||||||  ||| ||       |||||
CUC  UUUCACU CG  GACCU CGUC CCAA UAGCUGG  AAG AC       AACAC  G
^  CG        -  C-     A    U    A       CU   A  GA-----     AA
       110         100        90        80             70
```

gma-MIR166a-5p GGAAUGUUGUCUGGCUCGAGG
gma-MIR166a-3p UCGGACCAGGCUUCAUUCCCC
Gm16:1912570-1912715, 146nt, (-)
ACGGAAGCUUUGUCUUUUGAGGGGAAUGUUGUCUGGCUCGAGGACCCUUCUUCAUCUUGAUCUUGUGUAGACUACUAUGCUUGUGGUCAAGGAAUACAUAGUGUUGUCGGACCAGGCUUCAUU
CCCCCCAAUUAUAUGCUUCCAAA

Structure 1 Folding bases 1 To 146 of 10Sep29-10-59-40 Initial dG = -62.10

```
         10        20        30        40        50         60          70
AC-      UU   CUU   A        UU      CU   G   C  UCUUCA-        UU--|     A
   GGAAGC  UGU   UUG GGGGAAUG  GUCUGG  CGA GAC CU       UCUUGAUC    GUGUAG C
   ||||||  |||   ||| ||||||||  ||||||  ||| ||| ||       ||||||||    ||||||
   CCUUCG  AUA   AAC CCCCUUAC  CGGACC  GCU UUG GA       GGAACUGG    CGUAUC U
AAA      U-   UU-   C        UU      AG   G   U  UACAUAA        UGUU^     A
    140         130       120       110       100        90        80
```

gma-MIR166c-5p GGAAUGUCGUCUGGUUCGAG
gma-MIR166c-3p UCGGACCAGGCUUCAUUCCCC
Gm02:14340767-14340863, 97nt, (+)
GUUGAGGGGAAUGUCGUCUGGUUCGAGACCAUUCAUGCAAGUAGUCUCAGACAUGACUCUUCUGAGUGAUUUCGGACCAGGCUUCAUUCCCCUCAGC

Structure 1 Folding bases 1 To 97 of 10Sep29-10-43-46 Initial dG = -57.00

```
        10        20        30        40        50
|            UC             C      UGC   U    UCA
GUUGAGGGGAAUG  GUCUGGUUCGAGA CAUUCA   AAG AGUC   G
|||||||||||||  ||||||||||||| ||||||   ||| ||||
CGACUCCCCUUAC  CGGACCAGGCUUU GUGAGU   UUC UCAG   A
^            UU             A      C--   -    UAC
      90        80        70          60
```

gma-MIR166d-5p GGAAUGUUGUUUGGCUCGAGG
gma-MIR166d-3p UCGGACCAGGCUUCAUUCCCG
Gm08:14990547-14990731, 185nt, (+)
GGAUGAUGGGAAUGUUGUUUGGCUCGAGGUAACUGCAUGGUCUUAAUUUUGUUCAUCUUUUGAAGCUUUAAUUUAUUUAUGGGUUUCAAUCUUUUUUGAUCCCUUGAAACAGAAAAAGCUUUA
AAGGUUGGAUUUUGAGGCUAUCCCUUUAUGUGAUCUCGGACCAGGCUUCAUUCCCGUCAACC

Structure 1 Folding bases 1 To 185 of 10Sep29-10-45-36 Initial dG = -72.60

```
        10        20        30             40        50        60        70        80        90
  A           UU      CU      A  UGC-----|         UUUU     U             AAUUUA    U   UU    UC
GG UGAUGGGAAUG  GUUUGG  CGAGGU AC        AUGGUCUUAA    GUUCA CUUUUGAAGCUUU      UUUA GGG  UCAA  U
|| |||||||||||  ||||||  |||||| ||        ||||||||||    ||||| |||||||||||||      |||| |||  ||||
CC ACUGCCCUUAC  CGGACC  GCUCUA UG        UAUCGGAGUU    UAGGU GGAAAUUUCGAAA      AAGU CCC  AGUU  U
  A           UU      AG      G  UAUUUCCC^         U---     U             AAGACA    U   U-    UU
   180       170       160       150       140          130       120       110       100
```

gma-MIR166e-5p GGAAUGUUGUUUGGCUCGAGG
gma-MIR166e-3p UCGGACCAGGCUUCAUUCCCG
Gm15:3688764-3688931, 168nt, (-)
GGUUGAUGGGAAUGUUGUUUGGCUCGAGGUAACUAUGCAUGGUCUUAAUUUUGUUCAUCUUUGAAGCUUUAAUUUAUGGGUUUCGAUCUCUUUGAUCCCUUGAAACAAAGAAAGCUUUAAAGG
UUGGAUUUUGAGGCUUUCUCGGACCAGGCUUCAUUCCCGUAAACC

Structure 1 Folding bases 1 To 168 of 10Sep29-10-51-35 Initial dG = -63.90

```
        10        20          30
    G         UU      CU      |  A
GGUU AUGGGAAUG  GUUUGG  CGAG--GUA C
|||| |||||||||  ||||||  ||||  |||
CCAA UGCCCUUAC  CGGACC  GCUC  CGU U
    A         UU      AG    \ ^  A
      160       150
```

```
                              40        50          60        70            80
                              AU        UUUU     -            AA----    U   UU    UC
                                GGUCUUAA    GUUCA UCUUUGAAGCUUU      UUUA GGG  UCGA  \
                                ||||||||    ||||| |||||||||||||      |||| |||  ||||
                                UCGGAGUU    UAGGU GGAAAUUUCGAAA      AAGU CCC  AGUU  U
                              UU        U---     U            GAAACA    U   U-    UC
                            140       130          120        110       100         90
```

gma-MIR166f UCUCGGACCAGGCUUCAUUCC
gma-MIR166f(iso) UCUCGGACCAGGCUUCAUUC
Gm20:43105394-43105500, 107nt, (-)
AGGAGUUGAGGGGAAUGGUGUCUGGUUCGAGACCAUUCUUCUGAAGCAAAGAUCAUCAUCAUCACCCUUGAGAAUGAUCUCGGACCAGGCUUCAUUCCCCCUAGCUC

Structure 1 Folding bases 1 To 107 of 10Sep29-11-01-57 Initial dG = -52.70

```
        10        20        30        40        50
AG|     A         U             C       CUG   CAAA   CAU
  GAGUUG GGGGAAUGG GUCUGGUUCGAGA CAUUCUU   AAG    GAU   \
  |||||| ||||||||| ||||||||||||| |||||||   |||    |||
  CUCGAU CCCCUUACU CGGACCAGGCUCU GUAAGAG   UUC    CUA   C
--^     C         U             A       ---   CCA-   CUA
       100        90        80        70            60
```

gma-MIR167c UGAAGCUGCCAGCAUGAUCUG
Gm07:39778512-39778886, 375nt, (+)
UUUGAGAGGUUGAAGCUGCCAGCAUGAUCUGGUAAAUCACAUACUUUUUUUUUUCUCACCUCUCAUGCCUAAUUUUUAAGCACCAGUCAUUAGAGAAAAUAAUGGUGAAAAAUCCAUCUAUUC
AAUUUUUUUUUUCAAAUUCAAGGUUUCCAGUAUGUAUCACUAAUGGUGAAAAAAGUGAUGGAAUUUUGUAGAACAUGGGUUAAAUUUACUUUUUUUUUUUUUGAGUUUUCAUUUUCUUCAAGU

UUCUGAGCCAAGAAAUAAAAGAGACUUAUAAAUUGGAAUUAAUACUUAAAGGAAACCCACCAGAAGGGCAAUUUGGUUAUCAUAAGAUGUGGUUUCCAUCAGGUCAUCUUGCAGCUUCAAUCA
CUCAAU

Structure 1 Folding bases 1 To 375 of 10Sep29-11-04-02 Initial dG = -97.70

```
        10        20        30          40        50        60        70            80
U     A            C  C          --         A   UUUUUUUUUCUC  UCUCA     .-AA       --| A
 UUGAG GGUUGAAGCUGC AG AUGAUCUGGU  AAAUCACAU CUU           ACC      UGCCU    UUUUUAA  GC C
 ||||| |||||||||||| || ||||||||||  ||||||||| |||           |||      |||||    |||||||  ||
 AACUC CUAACUUCGACG UC UACUGGACUA  UUUGGUGUA GAA           UGG      ACGGG    AGAGAUU  UG C
U     A            U  -          CC         -   UACUAU-----   UUUA-     \ --       AC^ A
   370       360        350       340        330            320                   90
```

```
  100         110          120       130       140       150         160
 AAAUAA     .-AAA      ---    UUCAAUUUUUUUUUUCAAAUU       U   G   GUA--    UA
       UGGUG     AAUCCA   UCUA                     CAAGGUU CCA UAU     UCAC  \
       |||||     ||||||   ||||                     ||||||| ||| |||     ||||
       ACCAC     UUGGGU   AGAU                     GUUUUAA GGU GUG     AGUG  A
 AAG---     \ ---      ACA    ---------------------       -   A   AAAAA    GU
310                200                            190        180       170
```

```
   210       220            230       240       250
AAAUUUACUUUUU            -----     U   CUUC        GAGCCAA
             UUUUUUUUGAGU     UUUCA UUU    AAGUUUCU       \
             ||||||||||||     ||||| |||    ||||||||
             AAAGGAAAUUCA     AAGGU AAA    UUCAGAGA       G
CC-----------            UAAUU     U   UA--        AAAUAAA
            300       290       280         270       260
```

gma-MIR167g(iso) UGAAGCUGCCAGCAUGAUCUG

Gm10:39044877-39044954, 78nt, (+)

CAGCAGUUGAAGCUGCCAGCAUGAUCUGAGUUUACCUUCUAUUGGUAAGAACAGAUCAUGUGGCUGCUUCACCUGUUG

Structure 1 Folding bases 1 To 78 of 10Sep29-11-14-32 Initial dG = -44.90

```
        10        20        30
      U      U    G         AG      UUC
CAGCAG UGAAGC GCCA CAUGAUCUG  UUUACC   \
|||||| |||||| |||| |||||||||  ||||||
GUUGUC ACUUCG CGGU GUACUAGAC  GAAUGG   U
      C      U    -         AA      UUA
       70         60        50
```

gma-MIR167h UGAAGCUGCCAGCAUGAUCUG

Gm20:44765096-44765173, 78nt, (+)

CAGCAGUUGAAGCUGCCAGCAUGAUCUGAGUUUACCUUCUAUUGGUAAGAACAGAUCAUGUGGCUGCUUCACCUGUUG

Structure 1 Folding bases 1 To 78 of 10Sep29-11-16-29 Initial dG = -44.90

```
        10        20        30
|     U      U    G         AG      UUC
CAGCAG UGAAGC GCCA CAUGAUCUG  UUUACC   \
|||||| |||||| |||| |||||||||  ||||||
GUUGUC ACUUCG CGGU GUACUAGAC  GAAUGG   U
^     C      U    -         AA      UUA
       70         60        50
```

gma-MIR168a UCGCUUGGUGCAGGUCGGGAA
Gm09:41353226-41353350, 125nt, (-)
CACUGUGCGGUCUCUAAUUCGCUUGGUGCAGGUCGGGAACCGGUUUUCGCGCGGAAUGGAGGAGCGGUCGCCGGCGCCGAAUUGGAUCCCGCCUUGCAUCAACUGAAUCGGAGGCCGCGGUGA
AC

Structure 1 Folding bases 1 To 125 of 10Sep29-11-19-40 Initial dG = -66.20

```
           10        20        30        40        50          60
---|   GU        UA     C          U     A       UC     -  AA--  GA
   CACU  GCGGUCUC  AUUCG UUGGUGCAGG CGGGA CCGGUUU  GCGC GG     UG  G
   ||||  ||||||||  ||||| |||||||||| ||||| |||||||  |||| ||     ||
   GUGG  CGCCGGAG  UAAGU AACUACGUUC GCCCU GGUUAAG  CGCG CC     GC  G
CAA^   --        GC     C          C     A       C-     G  GCUG  GA
   120         110       100        90        80         70
```

gma-MIR168b UCGCUUGGUGCAGGUCGGGAA
Gm01:48070311-48070420, 110nt, (-)
CGGUCUCUAAUUCGCUUGGUGCAGGUCGGGAACCGGUUUUCGCGCGGAAUGGAGGAACGGUCGCCGGCGGCGAAUUGGAUCCCGCCUUGCAUCAACUGAAUCGGAGGCCG

Structure 1 Folding bases 1 To 110 of 10Sep29-11-18-03 Initial dG = -60.40

```
        10        20        30         40          50
       UA     C          U     A       -    G   AA--| GA
CGGUCUC  AUUCG UUGGUGCAGG CGGGA CCGGUUU UCGC CGG    UG  G
|||||||  ||||| |||||||||| ||||| ||||||| |||| |||    ||
GCCGGAG  UAAGU AACUACGUUC GCCCU GGUUAAG GGCG GCC    GC  G
       GC     C          C     A       C    -   GCUG^ AA
        100        90        80         70         60
```

gma-MIR169a CAGCCAAGGAUGACUUGCCGG
Gm09:35771804-35771924, 121nt, (+)
AAGAGGAAGAGAGAGUGAUGCAGCCAAGGAUGACUUGCCGGCGUUAUUAUUUGCUCAUGUUCAUGCUCACCGGUUUCCUUGCCGGCAAGUUGUGUUUGGCUAUGUUUUGCUCUCUUCUUCU

Structure 1 Folding bases 1 To 121 of 10Sep29-12-01-27   Initial dG = -57.20

```
        10        20        30        40        50          60
A            AGU   GC       G UG           UUAUU   --| CU   G
 AGAGGAAGAGAG   GAU  AGCCAAG A  ACUUGCCGGCG     AUU  UG  CAU U
 ||||||||||||   |||  ||||||| |  |||||||||||     |||  ||  |||
 UCUUCUUCUCUC   UUG  UCGGUUU U  UGAACGGCCGU     UGG  AC  GUA U
-            GUU   UA       G GU           UCCUU   CC^ UC   C
120       110       100        90        80        70
```

gma-MIR169f-5p UAGCCAAGAAUGACUUGCCGG
gma-MIR169f-3p UUUCGACGAGUUGUUCUUGGC
Gm02:46876643-46876727, 85nt, (-)
GUAGCCAAGAAUGACUUGCCGGAAUGCAUGCAUUUAUUAGGUACCAAGGUGUAUUGUAUGAUUUCGACGAGUUGUUCUUGGCUAC

Structure 1 Folding bases 1 To 85 of 10Sep29-11-50-35 Initial dG = -39.10

```
        10        20        30         40
|          UG     C      G      UU-    AGGU
GUAGCCAAGAA  ACUUG CGGAAU CAUGCA   UAUU    \
|||||||||||  ||||| |||||| ||||||   ||||
CAUCGGUUCUU  UGAGC GCUUUA GUAUGU   GUGG    A
^          GU     A      -      UAU    AACC
    80        70         60        50
```

gma-MIR169g CAGCCAAGAAUGACUUGCCGG
gma-MIR169g(iso) AGCCAAGAAUGACUUGCCGG
Gm09:5263992-5264096, 105nt, (+)
GAGUGAUUUGCAGCCAAGAAUGACUUGCCGGAAUGCUUGCAUUAGGCAUAAUAUAUAGUUGUAUACUUUAUAAUCCGGCAAGUUGUUUUUGGCUACACUUUUUUC

Structure 1 Folding bases 1 To 105 of 10Sep29-11-54-56 Initial dG = -42.90

```
        10        20        30          40
   U  UU   C         UG            |      C
GAG GA  UG AGCCAAGAA  ACUUGCCGGA--AUGCUUG \
||| ||  || |||||||||  ||||||||||  |||||||
CUU UU  AC UCGGUUUUU  UGAACGGCCU  UACGGAU A
   U  UC  A         GU          \ ^      U
   100        90        80


                                 50        60
                                  AA    U   UG
                                    UAUA AGU  \
                                    |||| |||
```

```
                                    AUAU UCA  U
                                  A-    U   UA
                                     70
```

gma-MIR169h CAGCCAAGAAUGACUUGCCGG
gma-MIR169h(iso) AGCCAAGAAUGACUUGCCGG
Gm14:5324798-5324911, 114nt, (+)
GAGUGAUUUGCAGCCAAGAAUGACUUGCCGGAAUGCAUAUAUAUGCAUUAGGUACCAACAUAUAUAGUUGUAUUGUAUAAUUUCGGGCAAGUUGUUUUUGGCUACAUUUAUCUC

Structure 4 Folding bases 1 To 114 of 10Sep29-11-57-42 Initial dG = -40.60

```
       10        20        30        40         50
   UGAUU  C         UG       G   UGC|    U     -   G   CC
GAG     UG AGCCAAGAA  ACUUGCC GAA   AUAUA AUGCA UUA GUA  A
|||     || |||||||||  ||||||| |||   ||||| ||||| ||| |||
CUC     AC UCGGUUUUU  UGAACGG CUU   UAUGU UAUGU GAU UAU  A
   UAUUU  A         GU       G   UAA^    -     U   A   AC
  110       100        90        80         70        60
```

gma-MIR169i CAGCCAAGGAUGACUUGCCGG
Gm10:40332790-40332926, 137nt, (-)
AGAGAGUGUAGUGCAGCCAAGGAUGACUUGCCGGCAUUAGCCAAGUGAAUGAGCAUCAUAUAUAUAUAUAUAUAUAUAUAUAUAUAUAUAUGACUCAUGUUCUUGUCGGCAAGUUGGCCUUGGCU
AUAUUGGACUCUCU

Structure 1 Folding bases 1 To 137 of 10Sep29-12-03-25 Initial dG = -71.40

```
        10        20        30        40        50        60        70
       G     C        AU             UU   CAAGUGA     CA                 A
AGAGAGU UAGUG AGCCAAGG  GACUUGCCGGCA  AGC       AUGAG  UCAUAUAUAUAUAUAU U
||||||| ||||| ||||||||  ||||||||||||  |||       |||||  ||||||||||||||||
UCUCUCA GUUAU UCGGUUCC  UUGAACGGCUGU  UUG       UACUC  AGUAUAUAUAUAUAUA A
       G     A        GG             UC   -------     --                 U
     130       120       110       100                 90        80
```

gma-MIR169j CAGCCAAGGAUGACUUGCCGG
Gm13:368441-368563, 123nt,(-)
GAAAGUAGAGUGCAGCCAAGGAUGACUUGCCGGAGCAAUAAGCAAUAAUAUCUAUCUAUUUUAUCUUUCUUUUCUUCUUAUUCUUUCACGCCGGCAAGUUGUUCUUGGCUACAUUUUGUUUUC

Structure 1 Folding bases 1 To 123 of 10Sep29-12-06-19   Initial dG = -44.80

```
        10        20        30        40        50        60
            C         UG         AGCAAU   C     UAUCUAUCUAUUUU
GAAAGUAGAGUG AGCCAAGGA  ACUUGCCGG      AAG AAUAA              \
|||||||||||| |||||||||  |||||||||      ||| |||||
CUUUUGUUUUAC UCGGUUCUU  UGAACGGCC      UUC UUAUU              A
            A         GU         GCACU-   -     CUUCUUUUCUUUCU
 120       110       100        90          80        70
```

gma-MIR169k CAGCCAAGGGUGAUUUGCCGG
Gm15:14150069-14150183, 115nt, (+)
GGAGUGCAGCCAAGGGUGAUUUGCCGGCACAGGCACUAAUUAGUUCAAUAUUGAAUAGUUAGUUCUGUUUGAUUGAUUUACUUCUGUGCCGGCAAGUUUCUCUUGGCUACAUUUC

Structure 1 Folding bases 1 To 115 of 10Sep29-12-10-27   Initial dG = -56.80

```
        10        20        30        40        50
      C         U                CACU        C    UU    AG
GGAGUG AGCCAAGGG GAUUUGCCGGCACAGG    AAUUAGUU AAUA  GAAU  \
|||||| ||||||||| ||||||||||||||||    |||||||| ||||  ||||
CUUUAC UCGGUUCUC UUGAACGGCCGUGUCU    UUAGUUAG UUGU  CUUG  U
      A         U                UCAU        U    --    AU
   110       100        90        80        70          60
```

gma-MIR169l CAGCCAAGGAUGACUUGCCGG
Gm17:4861816-4861963, 148nt, (-)
AGAGGUAGAAAGUAGAGUGCAGCCAAGGAUGACUUGCCGGAGCAAUAAGAAUAAACAAUAAAAUGUAUCUAUUUAUAACAUUCUUCUUCUUCUUCUUCUUCUUUCACGCCGGCAAGUUGUUCU
UGGCUACAUUUUGUUUUCUUCUUCU

Structure 1 Folding bases 1 To 148 of 10Sep29-12-14-23   InitialdG = -52.80

```
        10        20        30        40              50        60        70
     U             C         UG          ------| C  U     UAAACAAUAA     AUCUA
AGAGG AGAAAGUAGAGUG AGCCAAGGA  ACUUGCCGG      AG AA AAGAA          AAUGU     \
||||| ||||||||||||| |||||||||  |||||||||      || || |||||          |||||
UCUUC UCUUUUGUUUUAC UCGGUUCUU  UGAACGGCC      UC UU UUCUU          UUACA     U
     U             A         GU          GCACUU^ -  C     CUUCUUCUUC     AUAUU
      140       130       120       110        100        90        80
```

gma-MIR171d UUGAGCCGUGCCAAUAUCACG
Gm06:48920631-48920715, 85nt, (-)
CGGGAUAUUGGUCCGGUUCAAUAAGAAAGCAAUGCUCAAAAUGUUAUUGGGUCCUGUUUUUUCAUUGAGCCGUGCCAAUAUCACG

Structure 1 Folding bases 1 To 85 of 10Sep29-12-17-50   Initial dG = -39.80

```
        10        20        30        40
| G        UC         A        AU      AAU
CG GAUAUUGG  CGGUUCAAU AGAAAGCA  GCUCAA   \
|| ||||||||  ||||||||| ||||||||  ||||||
GC CUAUAACC  GCCGAGUUA UUUUUUGU  UGGGUU   G
^ A        GU         C        CC      AUU
    80        70        60        50
```

gma-MIR171e CGAUGUUGGUGAGGUUCAAUC
Gm13:26271135-26271232, 98nt, (+)
GAGAAAGCGAUGUUGGUGAGGUUCAAUCCGAAGACGGAUUUACAUGUAGAAGCAGUAAAAUACGAUCUCAGAUUGAGCCGCGCCAAUAUCACUUUAUC

Structure 1 Folding bases 1 To 98 of 10Sep29-12-19-16 Initial dG = -41.20

```
        10        20        30          40
  G    C          A         C A   -  GA-|    A   A
GA AAAG GAUGUUGGUG GGUUCAAUC G AGA CG   UUUAC UGU G
|| |||| |||||||||| ||||||||| | ||| ||   ||||| |||
CU UUUC CUAUAACCGC CCGAGUUAG C UCU GC   AAAUG ACG A
  A    A          G         A -   A  AUA^    -   A
       90        80        70         60         50
```

gma-MIR171f CGAUGUUGGUGAGGUUCAAUC
Gm17:9101701-9101798, 98nt, (-)
GAGAAAGCGAUGUUGGUGAGGUUCAAUCCGAAGACGGAUUUACAUGUAGAAGCAGUAAAAUACGAUCUCAGAUUGAGCCGCGCCAAUAUCACUUUAUC

Structure 1 Folding bases 1 to 98 of 10Oct10-16-34-43   Initial dG = -41.20

```
        10        20        30          40
  G    C          A         C A   -  GA-|    A   A
GA AAAG GAUGUUGGUG GGUUCAAUC G AGA CG   UUUAC UGU G
|| |||| |||||||||| ||||||||| | ||| ||   ||||| |||
CU UUUC CUAUAACCGC CCGAGUUAG C UCU GC   AAAUG ACG A
  A    A          G         A -   A  AUA^    -   A
       90        80        70         60         50
```

gma-MIR172b-5p   GUAGCAUCAUCAAGAUUCAC
Gm13:40401688-40401809, 122nt, (-)
GCGGAUGUAGCAUCAUCAAGAUUCACAUGCAAAUGAAGGUGGGUGGGACUAUGAUGCAAUCCAAGUGCUCUGCCAAUCCAUCGGUCUUUUUGAUGUGAGAAUCUUGAUGAUGCUGCAUCAGC
Structure 1 Folding bases 1 To 122 of 10Sep29-12-45-16   Initial dG = -57.40

```
        10        20         30          40        50        60
  G                     A    -    U-  A-|      G  ACUAU  U   AUC
GC GAUGUAGCAUCAUCAAGAUUC CAUG CAAA  GA  GGUGGGU GG     GA GCA   \
|| ||||||||||||||||||||| |||| ||||  ||  ||||||| ||     || |||
CG CUACGUCGUAGUAGUUCUAAG GUGU GUUU  CU  CUACCUA CC     CU CGU   C
  A                     A    A    UU  GG^     A  GU---  -   GAA
120       110       100        90        80           70
```

gma-MIR172c GGAAUCUUGAUGAUGCUGCAG

gma-MIR172c(iso) GAAUCUUGAUGAUGCUGCAG

Gm18:2968986-2969138, 153nt, (+)

AAAUCAGUCACUGUUUGCCGGUGGAGCAUCAUCAAGAUUCACAAGCUUUAGGGGCAUUAAUUUGUUUGAGGUGGUUCCUUAUUGCUCCAAAACCAAUUAGCCCUUUUGCUAUGGGAAUCUUGA
UGAUGCUGCAGCAGCAAUAAAUGACUAAUA

Structure 1 Folding bases 1 To 153 of 10Sep29-12-47-32 Initial dG = -61.40

```
        10        20        30        40         50        60         70
AAAUC     CUGU    CGG  G                A  -|  UUU      AU     UG-    AG UG  UC
     AGUCA    UUGC   UG AGCAUCAUCAAGAUUC CA AGC   AGGGGC  UAAUU   UUUG  G  GU  \
     |||||    ||||   || |||||||||||||||| || |||   ||||||  |||||   ||||  |  ||
     UCAGU    AACG   AC UCGUAGUAGUUCUAAG GU UCG   UUCCCG  AUUAA   AAAC  C  UA  C
AUAA-     AAAU    ACG  G                G  A^  UU-      --     CCA    CU GU  UU
 150        140       130       120       110        100          90        80
```

gma-MIR172d(iso1) GGAAUCUUGAUGAUGCUGCAG

gma-MIR172d(iso2) GAAUCUUGAUGAUGCUGCAG

Gm14:5548752 - 5548901 150nt Frame: +1/+1

AAAACAGUCGCUGAUUGCAGAUGGAGCAUCAUCAAGAUUCACAAGCUUCAGGGGUUUUUUGUUUGGGGUGGUCCCUUAUUGCUCCCAAAUGAAUUAAGCCCUUUGAUAUGGGAAUCUUGAUGA
UGCUGCAGCAGCAAUAAACGACUAAACA

Structure 1 Folding bases 1 To 150 of 10Sep29-12-46-31 InitialdG = -66.90

```
        10        20        30        40         50          60         70
AAAAC     CUG     AGA  G                A  AGCU  -        --  UG      -|UG  CC
     AGUCG   AUUGC   UG AGCAUCAUCAAGAUUC CA    UC AGGGGUUU  UU  UUUGGG G  GU  \
     |||||   |||||   || |||||||||||||||| ||    || ||||||||  ||  |||||| |  ||
     UCAGC   UAACG   AC UCGUAGUAGUUCUAAG GU    AG UUCCCGAA  AA  AAACCC C  UA  C
ACAA-     AAA     ACG  G                G  AU--  U        UU  GU      U^GU  UU
.        140       130       120       110         100        90        80
```

gma-MIR172e(iso1) GGAAUCUUGAUGAUGCUGCAG

gma-MIR172e(iso2) GAAUCUUGAUGAUGCUGCAG

Gm11:35957808-35957960, 153nt, (-)
AAAACAGUCACUGUUUGCCGGUGGAGCAUCAUCAAGAUUCACAAGCUUUAGGGGGCAUUAAUUUGUUUGAGGUGGUCCCUUAUUGAUCCAAACCAAUUAGCCCUUUUGCUAUGGGAAUCUUGA
UGAUGCUGCAGCAGCAAUAAAUGACUAAUA

Structure 1 Folding bases 1 To 153 of 10Sep29-12-40-39   Initial dG = -58.30

```
        10        20        30        40         50        60        70        80
AAAAC     CUGU    CGG  G                A  -|  UUU       AU     U     AGGU    CCU
     AGUCA    UUGC   UG AGCAUCAUCAAGAUUC CA AGC   AGGGGGC  UAAUU GUUUG    GGUC   \
     |||||    ||||   || |||||||||||||||| || |||   |||||||  ||||| |||||    ||||
     UCAGU    AACG   AC UCGUAGUAGUUCUAAG GU UCG   UUUCCCG  AUUAA CAAAC    CUAG   U
AUAA-     AAAU    ACG  G                G  A^  U--       --     C     ----    UUA
 150        140       130       120       110         100          90
```

gma-MIR172g GCAGCACCAUCAAGAUUCAC
Gm10:31592576-31592689,114nt, (-)
GCAGGUGCAGCACCAUCAAGAUUCACAUGCAAUUUACACCCUAUAAGAGAUUUUUGACAAAAUUUCUUUCCUAGGAGAGUUUUGACUUGAGAAUCUUGAUGAUGCUGCAUCAGC

Structure 1 Folding bases 1 To 114 of 10Sep29-12-23-59   InitialdG = -45.20

```
        10        20         30        40          50
  A         C           A  UG-   UUUACAC    U--|         U
GC GGUGCAGCA CAUCAAGAUUC CA   CAA       CCUA   AAGAGAUUUU G
|| ||||||||| ||||||||||| ||   |||       ||||   ||||||||||
CG CUACGUCGU GUAGUUCUAAG GU   GUU       GGAU   UUCUUUAAAA A
  A         A           A  UCA   UUGAGA-    CCU^         C
  110       100        90        80         70        60
```

gma-MIR172h-5p GCAGCAGCAUCAAGAUUCACA
gma-MIR172h-5p(iso) GCAGCAGCAUCAAGAUUCAC
gma-MIR172h-3p AGAAUCUUGAUGAUGCUGCAU
Gm10:43474733-43474823, 91nt,(+)
GCAGGUGCAGCAGCAUCAAGAUUCACACACAGAUUUACCUCCUUGGGGGCGUGUGUUUCGGUGCUGAGAAUCUUGAUGAUGCUGCAUCAGC
Structure 1 Folding bases 1 To 91 of 10Sep29-12-27-57InitialdG = -44.10

```
        10        20         30           40
  A         G           A  -   A  UU--   -|   U
GC GGUGCAGCA CAUCAAGAUUC CA CAC GA    UAC CUCC \
|| ||||||||| ||||||||||| || ||| ||    ||| ||||
CG CUACGUCGU GUAGUUCUAAG GU GUG CU    GUG GGGG U
  A         A           A  C   G  UUGU   C^   G
90        80        70        60        50
```

gma-MIR172i GCAGCAGCAUCAAGAUUCACA
gma-MIR172i(iso) GCAGCAGCAUCAAGAUUCAC
Gm15:2892962-2893122, 161nt, (-)
GCAGGUGCAGCAGCAUCAAGAUUCACACCGCCUAAUUUGCUAGGACUUCAGGACUGCACACGCUUAAUUAUACAUACAUAUAUACAUAUAUGUUAGCUCUUUGUGGAGUGCGGAAUAAAGUUC
UAUUUUAGAUGUGGGAAUCUUGAUGAUGCUGCAUCAGC

Structure 1 Folding bases 1 To 161 of 10Sep29-12-49-36 Initial dG = -60.10

```
        10        20        30        40        50        60         70        80
| A         G           A   CGC    UUUGC        CAGGA      A-   UUAAUUAUACAU       U
GC GGUGCAGCA CAUCAAGAUUC CAC   CUAA     UAGGACUU     CUGCAC  CGC            ACAUAUA A
|| ||||||||| ||||||||||| |||   ||||     ||||||||     ||||||  |||            |||||||
CG CUACGUCGU GUAGUUCUAAG GUG   GAUU     AUCUUGAA     GGCGUG  GUG            UGUAUAU C
^ A         A           G   UA-    UU---        AUAA-      AG   UUUCUCGAU---       A
 .       150       140        130          120        110       100           90
```

gma-MIR172j GCAGCAGCAUCAAGAUUCACA
gma-MIR172j(iso) GCAGCAGCAUCAAGAUUCAC
Gm20:40895747-40895836, 90nt, (-)
GCAGGUGCAGCAGCAUCAAGAUUCACACACAGAUUCCACCUCCUUGGGGGAGUGUUUAGGUGCUGAGAAUCUUGAUGAUGCUGCAUCAGC
Structure 1 Folding bases 1 To 90 of 10Sep29-12-51-11 Initial dG = -44.50

```
        10        20         30         40
  A         G           A  -|  AGAUUC   CU  U
GC GGUGCAGCA CAUCAAGAUUC CA CAC      CAC  CC U
|| ||||||||| ||||||||||| || |||      |||  ||
CG CUACGUCGU GUAGUUCUAAG GU GUG      GUG  GG G
  A         A           A  C^  GAUUU-   AG  G
.        80        70        60         50
```

gma-MIR319a(iso) UUGGACUGAAGGGAGCUCCCU
Gm05:40832097-40832279, 183nt, (+)

AAGGUAAGAGAGCUUUCUUCAGUCCACUCAUGGGUGACAGUAAGAUUCAAUUAGCUGCCGACUCAUUCAUCCAAAUGUUGAGUGUAAGCGAAUAAAUAUACUCAGCAGAUGAGUGAAUGAUGC
GGGAGACAAAUUGAAUCUUAAGUUUCCUGUACUUGGACUGAAGGGAGCUCCCUUUUCCUU

Structure 1 Folding bases 1 To 183 of 10Sep29-13-02-53   InitialdG = -88.00

```
         10        20        30        40        50         60        70        80        90
    U-|  A     UU          CUC     U   AG            AG-  G   AC       UC  AA           AGCG
AAGG  AAG GAGCU  CUUCAGUCCA   AUGGG GAC   UAAGAUUCAAUU   CU CCG  UCAUUCA  CA  UGUUGAGUGUA    A
||||  ||| |||||  ||||||||||   ||||| |||   ||||||||||||   || |||  |||||||  ||  |||||||||||
UUCC  UUC CUCGA  GAAGUCAGGU   UGUCC UUG   AUUCUAAGUUAA   GA GGC  AGUAAGU  GU  ACGACUCAUAU    A
    UU^  C     GG          UCA     U   A-            ACA  G   GU       GA  AG           AAAU
 180       170       160       150        140       130       120       110       100
```

gma-MIR319b(iso) UUGGACUGAAGGGAGCUCCCU
Gm08:1647811-1647990, 180nt, (-)
GGUAAGAGAGCUUUCUUCAGUCCACUUAUGGGUGACAAUAAGAUUUCAAUUAGCUGCCGACUCAUUCAUCCAAAUGCUGAGUGAAAGCGAAGAAAGAUACUCAGCAAAUGAGUGAAUGAUGCG
GGAGACAAAUUGAUUCUUAAGUUUCCUGUACUUGGACUGAAGGGAGCUCCCUUUUUC

Structure 1 Folding bases 1 To 180 of 10Sep29-13-05-47   InitialdG = -78.50

```
         10        20        30        40        50         60        70        80        90
GGU-|  A     UU          CU      U   AA     UU      AG-  G   AC       UC  AA         AAAGCG
    AAG GAGCU  CUUCAGUCCA  UAUGGG GAC  UAAGA  UCAAUU   CU CCG  UCAUUCA  CA  UGCUGAGUG      A
    ||| |||||  ||||||||||  |||||| |||  |||||  ||||||   || |||  |||||||  ||  |||||||||
    UUC CUCGA  GAAGUCAGGU  AUGUCC UUG  AUUCU  AGUUAA   GA GGC  AGUAAGU  GU  ACGACUCAU      A
CUUU^  C     GG          UC      U   A-     U-      ACA  G   GU       GA  AA         AGAAAG
.       170       160       150        140        130       120       110       100
```

gma-MIR319d UUGGACUGAAGGGAGCUCCUUC
gma-MIR319d(iso) GGACUGAAGGGAGCUCCUUC
Gm02:43885398-43885595, 198nt, (+)
GGGAAAGAGAGUGAAGGAGUUUCCCUCAGCCCAUUCAUGGAUAUAAUGAAAGAUUGGGUUGCUGAAUUAACUGAUUCAUUCAUACAAUAGUAUUCAAUUAGGGUAAUAUUGUGUGAAUGAAGA
GAGUAUAGUAUCUAUAUUGGAACCCCCUCUUUCUCUGUGCUUGGACUGAAGGGAGCUCCUUCUUUUUCUGUUCCC

Structure 2 Folding bases 1 To 198 of 10Sep29-12-55-08   Initial dG = -83.80

```
         10        20        30        40        50        60                70          80        90
     -      U            C    C   UU      UAUAAU      UU     G UG -------|   -   GA-               G     AA
GGGAA AGAGAG GAAGGAGUUUCC UCAG CCA  CAUGGA      GAAAGA  GGGUU C  A       AUUA ACU   UUCAUUCAUACAAUA UAUUC  \
||||| |||||| |||||||||||| |||| |||  ||||||      ||||||  ||||| |  |       |||| |||   ||||||||||||||| |||||
CCCUU UCUUUU CUUCCUCGAGGG AGUC GGU  GUGUCU      CUUUCU  CCCAA G  U       UGAU UGA   AAGUAAGUGUGUUAU AUGGG  U
     G      U            A    A   UC      ------      CC     - GU AUAUCUA^   A   GAG               A     AU
      190       180       170       160             150        140       130       120       110       100
```

gma-MIR319e UUGGACUGAAGGGAGCUCCCU

Gm02:45704227-45704412, 186nt, (+)

AGUUGAAGAGAGCUUCCUUCAGUCCACUCAUGGAUGGGAAAGGGGUUUGAAUUAGCUGCUGACUCAUUCAUUCAAACACAAUAGAUUCGGCUUCAUGAUAUGUUAUUGUGAAUGUGUGAAUGA
UGCGGGAGGUAAAUUUCUUCCUUUUCUUGUCCUUGCUUGGACUGAAGGGAGCUCCCUCUAACU

Structure 1 Folding bases 1 To 186 of 10Sep29-12-58-00 Initial dG = -87.20

```
        10        20        30        40        50        60        70        80          90
     A  A                 CU  U   UG         UUU  A   G  G   AC        U  AA        AU--|  GC
AGUUG AG GAGCUUCCUUCAGUCCA  CA GGA  GGAAAGGGG   GA UUA CU CUG  UCAUUCAU CA  CACAAUAG    UCG  U
||||| || |||||||||||||||||  || |||  |||||||||   || ||| || |||  |||||||| ||  ||||||||    |||
UCAAU UC CUCGAGGGAAGUCAGGU  GU CCU  UCUUUUCCU   UU AAU GA GGC  AGUAAGUG GU  GUGUUAUU    AGU  U
     C  C                 UC  U   GU         UCU  A   G  G   GU        U  AA        GUAU^  AC
    180       170       160       150       140       130       120       110       100
```

gma-MIR319f UUGGACUGAAGGGGAGCUCCUUC

gma-MIR319f(iso) UGGACUGAAGGGGAGCUCCUUC

Gm04:46348798-46348991, 194nt, (+)

AAGAGAGUGAAGGAGCUUCCUUCAGCCCACGCAUGGGUUCGGGGGAUUGAAGGGUUGCUGAAGCAUCUGCUGACUCAUUCAUACACACAGAAUAGUGAUUCAAAUUGCUAAGGUAAUUGUGUG
AAUGAAGCAGGAGAUAUUUUGCAUCCCUCUUUCUUUGUGCUUGGACUGAAGGGGAGCUCCUUCUUUCUGUU

Structure 2 Folding bases 1 To 194 of 10Sep29-12-59-20 Initial dG = -84.40

```
        10         20        30        40        50        60        70        80        90       100
  G    U         -        C   C   UG  UUC GG   UUGAA   U   U   GC    G   AC          CACAGAA   UG| U
AA AGAG GAAGGAGCU UCCUUCAG CCA GCA  GG   G  GGA     GGG UGC GAA  AUCU CUG  UCAUUCAUACA       UAG  AU C
|| |||| ||||||||| |||||||| ||| |||  ||   |  |||     ||| ||| |||  |||| |||  |||||||||||       |||  ||
UU UCUU CUUCCUCGA GGGAAGUC GGU CGU  UU   C  UCU     CCC ACG UUU  UAGA GAC  AGUAAGUGUGU       AUC  UA A
  G    U         G        A   U   GU  --- UU   -----   U   U   A-    G   GA          UAAUGGA   GU^ A
  190       180       170       160               150       140        130       120       110
```

gma-MIR319g UUGGACUGAAGGGAGCUCCCU

Gm11:1374020-1374198, 179nt, (+)
AGGUAAGAGAGCUCUCUUCAGCCCACUCAUAGGUGAUAAUAGGAUUUAAUUAGCUGCCGACUCAUUCAUACACAUGCUGAGUGAAUUAAUGAAUAAUACUCAGUAAAUGAGUGAAUGAUACGG
GAGACAAAUUGAAUCUUAUGUUUUCUGUACUUGGACUGAAGGGAGCUCCCUUUUCU

Structure 1 Folding bases 1 To 179 of 10Sep29-13-10-14   Initial dG = -74.20

```
        10        20        30        40        50         60        70        80        90
|  U   A             C   CUC     U    A            AG-  G   AC        A  CA        GA    AU
AGG AAG GAGCUCUCUUCAG CCA   AUAGG GAUA UAGGAUUUAAUU   CU CCG  UCAUUCAU CA  UGCUGAGU  AUUA  \
||| ||| ||||||||||||| |||   ||||| |||| ||||||||||||   || |||  |||||||| ||  ||||||||  ||||
UCU UUC CUCGAGGGAAGUC GGU   UGUCU UUGU AUUCUAAGUUAA   GA GGC  AGUAAGUG GU  AUGACUCA  UAAU  G
^  U   C             A   UCA     U    -            ACA  G   AU        A  AA        --    AA
       170       160       150        140       130       120       110       100
```

gma-MIR319h UUGGACUGAAGGGAGCUCCCU
Gm11:32902062-32902247, 186nt, (+)
AGUUUAAGAGAGCUUCCUUCAGUCCACUCAUGGAUGGGUAGAGGGUUUGAAUUAGCUGCUGACUCAUUCAUUCAAACACAAUAGAAUUAGUAUCAUGGUAUGCUAUUGUGAAUGUGUGAAUGA
UGCAGGAGGUAAAGUUCAUCCUUUUCUUGUCUUUGCUUGGACUGAAGGGAGCUCCCUUUUACU

Structure 1 Folding bases 1 To 186 of 10Sep29-14-00-51   Initial dG = -83.30

```
        10        20        30        40        50          60        70        80        90
|  UU   A                CU  U   UG  U      UU     --  G  G   AC         U  AA         AAUUA    C
AGU   AAG GAGCUUCCUUCAGUCCA  CA GGA   GG AGAGGG  UGAAU  UA CU CUG  UCAUUCAU CA  CACAAUAG     GUAU A
|||   ||| |||||||||||||||||  || |||   || ||||||  |||||  || || |||  |||||||| ||  ||||||||     ||||
UCA   UUC CUCGAGGGAAGUCAGGU  GU UCU   UC UUUUCC  ACUUG  AU GA GAC  AGUAAGUG GU  GUGUUAUC     UAUG U
^  UU   C                UC  U   GU  -      U-     AA  G  G   GU         U  AA         G----    G
    180       170       160       150         140       130       120       110           100
```

gma-MIR319i UUGGACUGAAGGGAGCUCCCU
Gm14:47959350-47959535, 186nt, (+)
AGUUGAAGAGAGCUUCCUUCAGUCCACUCAUGGAUGGGUAAGGGGUUAGAAUUAGCUGCUGACUCAUUCAUUCAAACACAAUAGAUCUGGCAUCAUGAUAUGCUAUUGUGAAUGUGUGAAUGA
UGCGGGAGGUAAAUUUCUUCCUUUUCCUGUCUGUGCUUGGACUGAAGGGAGCUCCCUCUAACU

Structure 1 Folding bases 1 To 186 of 10Sep29-14-03-37   Initial dG = -87.50

```
        10        20        30        40        50        60        70        80             90
     A  A                CU          U      UU   A   G  G   AC         U  AA         ---   -| G
AGUUG AG GAGCUUCCUUCAGUCCA  CAUGGAUGGG AAGGGG  AGA UUA CU CUG  UCAUUCAU CA  CACAAUAG   AUC UG C
||||| || |||||||||||||||||  |||||||||| ||||||  ||| ||| || |||  |||||||| ||  ||||||||   ||| ||
UCAAU UC CUCGAGGGAAGUCAGGU  GUGUCUGUCC UUUCCU  UUU AAU GA GGC  AGUAAGUG GU  GUGUUAUC   UAG AC A
     C  C                UC          U      UC   A   G  G   GU         U  AA         GUA   U^ U
    180       170       160       150       140       130       120       110       100
```

gma-MIR319j UUGGACUGAAGGGAGCUCCUUC

gma-MIR319j(iso) GGACUGAAGGGAGCUCCUUC
Gm14:45953433-45953649, 217nt, (-)
GGAGGGAAAGAGAGUGAAGGAGCUUCCCUCAGCCCAUUCAUGGAGAUAACGAAAGAUUGGGUUGCUGAAUUAACUGCUAGCUCACACAUUCAUUCAUACAAUAGUAUUCAAUUAGGGUAAUAU
UGUGUGAAUGAAGCGGGAGUAUAUAGUAUCUAUAUUGGAACCCUCUUUCUCUGUGCUUGGACUGAAGGGAGCUCCUUCUUUUUCUGCUCCCUCC

Structure 1 Folding bases 1 To 217 of 10Sep29-14-05-19 Initial dG = -101.20

```
         10        20        30        40        50        60        70           80        90       100       110
       A-|     U            C    C   UU        UAACGA    UU     G UG AU  AC     ---    ACACA               G     AA
GGAGGGA  AGAGAG GAAGGAGCUUCC UCAG CCA  CAUGGAGA      AAGA  GGGUU C  A  UA  UGCUA   GCUC     UUCAUUCAUACAAUA UAUUC  \
|||||||  |||||| |||||||||||| |||| |||  ||||||||      ||||  ||||| |  |  ||  |||||   ||||     ||||||||||||||| |||||
CCUCCCU  UCUUUU CUUCCUCGAGGG AGUC GGU  GUGUCUCU      UUCU  CCCAA G  U  AU  AUGAU   UGAG     AAGUAAGUGUGUUAU AUGGG  U
       CG^     U            A    A   UC        ------    --     - GU AU  CU     AUA    GGCG-               A     AU
     210       200       190       180             170          160       150       140        130       120
```

gma-MIR319k UUGGACUGAAGGGAGCUCCUUC
gma-MIR319k(iso1) UGGACUGAAGGGAGCUCCUUC
gma-MIR319k(iso2) GGACUGAAGGGAGCUCCUUC
Gm17:9436178-9436279, 102nt, (-)
UGAAAGGUGCAGAAAUAGGAGUUCCCUUGCAGCCCAAAACACCCUGCAUGAACUACUUCAUGUUGUUUUGGACUGAAGGGAGCUCCUUCUUCUUCACUUUCA

Structure 1 Folding bases 1 To 102 of 10Sep29-14-06-49 Initial dG = -51.90

```
        10        20        30        40        50
U|    G  C    AU            G   C        CCCU       C
 GAAAG UG AGAA  AGGAGUUCCCUU CAG CCAAAACA    GCAUGAA U
 ||||| || ||||  |||||||||||| ||| ||||||||    |||||||
 CUUUC AC UCUU  UCCUCGAGGGAA GUC GGUUUUGU    UGUACUU A
A^    -  U    CU            -   A        ----       C
100         90        80         70            60
```

gma-MIR3191 UUGGACUGAAGGGAGCUCCCU
Gm18:4278883-4279072, 190nt, (-)
AUAGUUUAAGAGAGCUUCCUUCAGUCCACUCAUGGAUGGAUAGAGGGUUUGAAUUAGCUGCUGACUCAUUCAUUCAAACACAAUAGAACGGGUGUCAUGGUAUGCUAUUGUGAAUGCCUGAAU
GAUGCAGGAGGUAAAGUUCAUCCUUUUCUUGUCUGUGCUUGGACUGAAGGGAGCUCCCUUUUACUGU

Structure 1 Folding bases 1 To 190 of 10Sep29-14-08-20 Initial dG = -82.90

```
        10        20        30        40        50          60        70        80        90
|    UU   A                 CU      UG AU      UU     --  G  G   AC       UU  AA        AACGG UGU
AUAGU  AAG GAGCUUCCUUCAGUCCA  CAUGGA  G  AGAGGG  UGAAU  UA CU CUG  UCAUUCA  CA  CACAAUAG     G   C
|||||  ||| |||||||||||||||||  ||||||  |  ||||||  |||||  || || |||  |||||||  ||  ||||||||     |
UGUCA  UUC CUCGAGGGAAGUCAGGU  GUGUCU  U  UUUCCU  ACUUG  AU GA GAC  AGUAAGU  GU  GUGUUAUC     U   A
^    UU   C                 UC      GU CU      --     AA  G  G   GU       CC  AA        GUA-- GGU
.       180       170       160       150         140       130       120       110         100
```

gma-MIR396a-5p UUCCACAGCUUUCUUGAACUG
gma-MIR396a-5p(iso1) UCCACAGCUUUCUUGAACUG
gma-MIR396a-3p UUCAAUAAAGCUGUGGGAAG
gma-MIR396a-3p(iso1) GUUCAAUAAAGCUGUGGGAAG
gma-MIR396a-3p(iso2) GUUCAAUAAAGCUGUGGGA
Gm13:26338134-26338273, 140nt, (-)
UCAUGGCUCUCUUUGUAUUCUUCCACAGCUUUCUUGAACUGCAUCCAAAGAGUUCCUUUGCAUGCAUGCCAUGGCACUCUUACUCCCAAAUCUUGUUUUGCGGUUCAAUAAAGCUGUGGGAAG
AUACAGAUAGGGUCAAC

Structure 1 Folding bases 1 To 140 of 10Sep29-14-12-04 Initial dG = -61.90

```
        10        20        30        40          50        60
UCA       C       UC            C          .-UCCA|     U  UUU   U
   UGGCUCU UUUGUAU  UUCCACAGCUUU UUGAACUGCA      AAGAGU CC   GCA G
   ||||||| |||||||  |||||||||||| ||||||||||      |||||| ||   |||
   ACUGGGA AGACAUA  AGGGUGUCGAAA AACUUGGCGU      UUCUCA GG   CGU C
CA-       U       GA            U          \ ----^     C  UAC   A
.        130       120       110       100       80        70


                                                    90
                                             ACUCC   A
                                                  CAA \
                                                  |||
                                                  GUU U
                                             UUU--   C
```

gma-MIR396b-5p UUCCACAGCUUUCUUGAACUU
gma-MIR396b-3p GCUCAAGAAAGCUGUGGGAGA
gma-MIR396b-3p(iso1) CUCAAGAAAGCUGUGGGAGA
Gm13:26329931-26330056, 126nt, (+)
CUCAAGUCCUGGUCAUGCUUUUCCACAGCUUUCUUGAACUUCUUAUGCAUCUUAUAUCUCUCCACCUCCAGGAUUUUAAGCCCUAGAAGCUCAAGAAAGCUGUGGGAGAAUAUGGCAAUUCAG
GCU

Structure 1 Folding bases 1 To 126 of 10Sep29-14-14-01 Initial dG = -49.30

```
        10            20        30        40        50        60
CUCA  U    ----|     C                   A     UAU  AUCUUAU   U  CCA
    AG CCUG    GUCAUG UUUUCCACAGCUUUCUUGA CUUCU   GC       AUC CU   C
    || ||||    |||||| ||||||||||||||||||| |||||   ||       ||| ||
    UC GGAC    CGGUAU AGAGGGUGUCGAAAGAACU GAAGA   CG       UAG GA   C
----  -    UUAA^     A                   C     UCC  AAUUU--   -  CCU
         120       110       100        90        80            70
```

gma-MIR396c UUCCACAGCUUUCUUGAACUU
Gm13:43804777-43804893, 117nt, (+)
CAACAAGUCCUGUUAUGCUUUUCCACAGCUUUCUUGAACUUCUUAUGCCUAGUGCAAUUAUUGAUGUGGCAUAGAAGUUUAAGAAAAAUGUGGAAAAACAUGUCAAAUCUAGGACUU

Structure 1 Folding bases 1 To 117 of 10Sep29-14-16-58 Initial dG = -46.80

```
        10              20        30        40        50
CAAC        U------|   C         GC              U     UAGUG    U
    AAGUCCUG       UAUG UUUUCCACA  UUUCUUGAACUUCU AUGCC     CAA \
    ||||||||       |||| |||||||||  |||||||||||||| |||||     |||
    UUCAGGAU       GUAC AAAAGGUGU  AAAGAAUUUGAAGA UACGG     GUU U
----        CUAAACU^   A         AA              -     UGUA-    A
         110       100        90        80         70         60
```

gma-MIR396d-5p UUCCACAGCUUUCUUGAACUU
gma-MIR396d-5p(iso) UUCCACAGCUUUCUUGAACU
gma-MIR396d-3p(iso1) GCUCAAGAAAGCUGUGGGAGA
gma-MIR396d-3p(iso2) CUCAAGAAAGCUGUGGGAGA

Gm17:9053051-9053155, 105nt, (-)

GUCAUGCUUUUCCACAGCUUUCUUGAACUUCUUAUGCAUCUUAUAUCUCUCCACUUCCAGCAUUUUAAGCCCUAGAAGCUCAAGAAAGCUGUGGGAGAAUAUGGC

Structure 1 Folding bases 1 To 105 of 10Sep29-14-30-13 Initial dG = -41.76

```
        10        20        30        40        50
|     C                   A     UAU  AUCUUAUAUCUCUCC
GUCAUG UUUUCCACAGCUUUCUUGA CUUCU   GC               \
|||||| ||||||||||||||||||| |||||   ||
CGGUAU AGAGGGUGUCGAAAGAACU GAAGA   CG               A
^     A                   C     UCC  AAUUUUACGACCUUC
   100        90        80        70        60
```

gma-MIR396e(iso1) UUCCACAGCUUUCUUGAACUG
gma-MIR396e(iso2) UUCCACAGCUUUCUUGAACU
gma-MIR396e(iso3) UCCACAGCUUUCUUGAACUG

Gm17:35366535-35366668, 134nt, (-)

GGUCUUUUCGUGAUCUUCCACAGCUUUCUUGAACUGUGUUGUGAGGCUUCUCUCCAAUGAAGGUUUAUACCCUAUGCAAAAGAAAUUCUAUGAGCACAAUUCAAGAUAGCUGUGGAAAAUCA
CUGAGAUGAUC

Structure 2 Folding bases 1 To 134 of 10Sep29-14-32-05 Initial dG = -50.50

```
        10        20        30        40         50        60
    U    C     C          U       C      -   AG C    CUCCA   AA|  U
GGUC UUUU GUGAU UUCCACAGCU UCUUGAA UGUGUU GUG  G UUCU     AUG  GGU U
|||| |||| ||||| |||||||||| ||||||| |||||| |||  | ||||     |||  |||
CUAG AGAG CACUA AAGGUGUCGA AGAACUU ACACGA UAU  U AAGA     UAU  CCA A
    U    U     A          U       A      G   CU A    AAACG   C-^  U
  130       120       110       100        90        80         70
```

gma-MIR396f   UCCACAGCUUUCUUGAACUG

Gm14:13971419-13971566, 148nt, (+)

GAAUGGUCUUUUUCGUGAUCUUCCACAGCUUUCUUGAACUGUGUUGUGUGAGGUUUCUCCAAGUGAAGGUUUAAGAUCCCUUAUGCAACAUAAAUUCUUUGAGCACAAUUCAAGAUAGCUGUG
GAAAAUCACUGAGAUGAUCUCGUUC

Structure 2 Folding bases 1 to 148 of Gm14 Initial dG = -56.90

```
          10        20        30        40          50             60
    --    U    C     C          U       C                UU   ----  AAG
GAAU  GGUC UUUU GUGAU UUCCACAGCU UCUUGAA UGUGU--UGUGUGAGG  UCU    CC   \
||||  |||| |||| ||||| |||||||||| ||||||| |||||  |||||||||  |||    ||
CUUG  CUAG AGAG CACUA AAGGUGUCGA AGAACUU ACACG  ACGUAUUCC  AGA    GG   U
    CU    U    U     A          U       A     \          CU   AUUU  AAG
      140       130       120       110               80        70



                                                 90
                                                A| UAAA
                                                 CA     \
                                                 ||
                                                 GU     U
                                                A^ UUCU
                                                100
```

gma-MIR396g   UUCCACAGCUUUCUUGAACUU

gma-MIR396g(iso)   UUCCACAGCUUUCUUGAACU

Gm15:556702 - 556796   95nt   Frame: +1/-1

UGUUAUGCUUUUCCACAGCUUUCUUGAACUUCUUAUGCCUAAUGCAGCUAUUGAUGUGGCAUUGAAGUUUAAGAAAAAUGUGGAAAAACAUGUCA

Structure 1 Folding bases 1 To 95 of 10Sep29-14-20-45 Initial dG = -39.00

```
        10        20        30        40        50
U|U    C         GC             UU      UAAU   GCU
 G UAUG UUUUCCACA  UUUCUUGAACUUC  AUGCC    GCA   A
 | |||| |||||||||  |||||||||||||  |||||    |||
 C GUAC AAAAGGUGU  AAAGAAUUUGAAG  UACGG    UGU   U
A^U    A         AA             U-      ----   AGU
    90        80        70         60
```

gma-MIR396h-5p UUCCACAGCUUUCUUGAACUG
gma-MIR396h-5p(iso1) UUCCACAGCUUUCUUGAACU
gma-MIR396h-5p(iso2) UCCACAGCUUUCUUGAACUG
gma-MIR396h-3p GUUCAAUAAAGCUGUGGGAAG
gma-MIR396h-3p(iso1) UUCAAUAAAGCUGUGGGAAG
gma-MIR396h-3p(iso2) GUUCAAUAAAGCUGUGGGA
Gm17:9044850-9044984, 135nt, (+)
UGGCCCUCUUUGUAUUCUUCCACAGCUUUCUUGAACUGCAUCCAUAGAGUUCCUUUGCAUGCAUGCCAAGGCACUCUUGCUCCCACACCUUGUUUUGCGGUUCAAUAAAGCUGUGGGAAGAUACAGAUAGGGUCA

Structure 2 Folding bases 1 To 135 of 10Sep29-14-22-31 Initial dG = -63.10

```
        10        20        30        40        50        60
U      C       UC             C          UCCAU  A| UCCUUU   UGCA   C
 GGCCCU UUUGUAU  UUCCACAGCUUU UUGAACUGCA     AG GU      GCA    UGC A
 |||||| |||||||  |||||||||||| ||||||||||     || ||      |||    |||
 CUGGGA AGACAUA  AGGGUGUCGAAA AACUUGGCGU     UC CA      CGU    ACG A
A      U       GA             U          UUUGU  -^ CACCCU   UCUC   G
   130       120       110       100        90         80         70
```

gma-MIR482a-5p(iso1) AUUUGUGGGAAUGGGCUGAUUGG
gma-MIR482a-5p(iso2) GGAAUGGGCUGAUUGGGAAGC
gma-MIR482a-5p(iso3) GAAUGGGCUGAUUGGGAAGC
gma-MIR482a-5p(iso4) AAUGGGCUGAUUGGGAAGC
Gm02:7783819-7783913, 95nt, (+)
UCAGAAUUUGUGGGAAUGGGCUGAUUGGGAAGCAAUGUGUGCUGGUGCAAUGCAUUUAAUUUCUUCCCAAUUCCGCCCAUUCCUAUGAUUUCUGA

Structure 1 Folding bases 1 To 95 of 10Sep29-14-34-28 Initial dG = -49.50

```
        10        20          30         40
      U UG             U-          C   GU-|   UGGU
UCAGAA U  UGGGAAUGGGC  GAUUGGGAAG AAU   GUGC    \
|||||| |  |||||||||||  |||||||||| |||   ||||
```

```
AGUCUU A  AUCCUUACCCG  UUAACCCUUC UUA   UACG    G
      U GU          CC          U   AUU^   UAAC
    90        80        70        60        50
```

gma-MIR482b-5p UAUGGGGGGAUUGGGAAGGAAU
gma-MIR482b-5p(iso1) UAUGGGGGGAUUGGGAAGGAA
gma-MIR482b-5p(iso2) UAUGGGGGGAUUGGGAAGGA
gma-MIR482b-5p(iso3) AUGGGGGGAUUGGGAAGGA
gma-MIR482b-3p UCUUCCCUACACCUCCCAUACC

Gm20:35360312-35360406, 95nt, (+)

GGGGGAAGGCAUGGGUAUGGGGGGAUUGGGAAGGAAUAUCCAUAAGCAAAAUAUGCUAUUUCUUCCCUACACCUCCCAUACCACUGUUUUUCCUC

Structure 1 Folding bases 1 To 95 of 10Sep29-14-40-10InitialdG = -50.40

```
        10        20         30        40
           UG           AUU-|          UC    AGC
GGGGGAAGGCA  GGUAUGGGGGG    GGGAAGGAAUA  CAUA   \
|||||||||||  |||||||||||    |||||||||||  ||||
CUCCUUUUUGU  CCAUACCCUCC    CCCUUCUUUAU  GUAU   A
           CA           ACAU^          C-    AAA
    90        80        70        60         50
```

gma-MIR482c AUUUGUGGGAAUGGGCUGAUUGG

Gm18:61452904-61453003, 100nt, (-)

GCAAUCAGAAUUUGUGGGAAUGGGCUGAUUGGGAAGUAAUGAGAUUGAGCAAUACAUUUAAUUUCUUCCCAAUUCCGCCCAUUCCUAUGAUUUCUGAUGC

Structure 1 Folding bases 1 To 100 of 10Sep29-14-35-41 Initial dG = -48.10

```
        10        20         30        40        50
|  A      U UG            U-         GUAAU          CAA
GCA UCAGAA U  UGGGAAUGGGC  GAUUGGGAA     GAGAUUGAG   \
||| |||||| |  |||||||||||  |||||||||     |||||||||
CGU AGUCUU A  AUCCUUACCCG  UUAACCCUU     CUUUAAUUU   U
^  -      U GU            CC         -----          ACA
.         90        80        70             60
```

gma-MIR482d-5p UAUGGGGGGAUUGGGAAGGAAU
gma-MIR482d-5p(iso1) UAUGGGGGGAUUGGGAAGGAA
gma-MIR482d-5p(iso2) UAUGGGGGAUUGGGAAGGA
gma-MIR482d-3p UCUUCCCUACACCUCCCAUACC
Gm10:48569629-48569723, 95nt, (-)
GGGGGAAGACAUGGGUAUGGGGGGAUUGGGAAGGAAUAUCCAUAAGCAAAAUAUGUUAUUUCUUCCCUACACCUCCCAUACCACUGUUUUUCCUC

Structure 1 Folding bases 1 To 95 of 10Sep29-14-37-51 Initial dG = -51.10

```
        10        20         30        40
           UG            AUU-|          UC     AGC
GGGGGAAGACA  GGUAUGGGGGG    GGGAAGGAAUA  CAUA   \
|||||||||||  |||||||||||    |||||||||||  ||||
CUCCUUUUUGU  CCAUACCCUCC    CCCUUCUUUAU  GUAU   A
           CA            ACAU^          U-     AAA
    90        80        70        60         50
```

gma-MIR1507a UCUCAUUCCAUACAUCGUCUGA
gma-MIR1507a(iso1) UCUCAUUCCAUACAUCGUCUGAC
gma-MIR1507a(iso2) UCUCAUUCCAUACAUCGUCUG
gma-MIR1507a(iso3) UCUCAUUCCAUACAUCGUCU
gma-MIR1507a(iso4) UCUCAUUCCAUACAUCGUC
gma-MIR1507a(iso5) UCAUUCCAUACAUCGUCUGA
gma-MIR1507a(iso6) CAUUCCAUACAUCGUCUGA
gma-MIR1507a(iso7) CAUUCCAUACAUCGUCUGACGA
Gm13:25849777-25849883, 107nt, (+)
CAGUGUUUGGCAGAGGUGUAUGGAGUGAGAGAAGGGAAAGGGUAUUUUCCGAUUCUGUCGUUACUCUCUUCCCUCUCUCAUUCCAUACAUCGUCUGACGAACGUAUC

Structure 1 Folding bases 1 To 107 of 10Sep29-14-43-01 Initial dG = -54.70

```
         10         20        30         40        50
CAG-      G    -                 A     -      UUUUC    U
    UGUUUG CAGA GGUGUAUGGAGUGAGAGA GGGAA AGGGUA     CGAU \
    |||||| |||| |||||||||||||||||| ||||| ||||||     ||||
    GCAAGC GUCU CUACAUACCUUACUCUCU CCCUU UCUCAU     GCUG C
CUAU      A    G                 -     C      U----    U
     100        90        80         70            60
```

gma-MIR1507b UCUCAUUCCAUACAUCGUCUG
gma-MIR1507b(iso1) UCUCAUUCCAUACAUCGUCUGAC
gma-MIR1507b(iso2) UCUCAUUCCAUACAUCGUCUGA
gma-MIR1507b(iso3) UCUCAUUCCAUACAUCGUCU
gma-MIR1507b(iso4) UCUCAUUCCAUACAUCGUC
gma-MIR1507b(iso5) UCAUUCCAUACAUCGUCUGA
gma-MIR1507b(iso6) CAUUCCAUACAUCGUCUGA
gma-MIR1507b(iso7) CAUUCCAUACAUCGUCUGACGA

Gm17:6190604-6190701, 98nt, (+)
GUUUGACAGAGAUGUAUGGAGUGAGAGAAGGGAAAUGAUAUUUUCCGAUCCCAUCGUUACUCUCUUCCCUCUCUCAUUCCAUACAUCGUCUGACGAAC

Structure 1 Folding bases 1 To 98 of 10Sep29-14-45-20 Initial dG = -47.70

```
        10         20        30        40        50
     A    -|                 A     AU  UAUUUUC   UC
GUUUG CAGA GAUGUAUGGAGUGAGAGA GGGAA  GA        CGA  \
||||| |||| |||||||||||||||||| |||||  ||        |||
CAAGC GUCU CUACAUACCUUACUCUCU CCCUU  CU        GCU  C
     A    G^                 -     CU  CAUU---   AC
       90        80        70          60
```

gma-MIR1508a(iso1) CUAGAAAGGGAAAUAGCAGUUG
gma-MIR1508a(iso2) CUAGAAAGGGAAAUAGCAGU
gma-MIR1508a(iso3) UAGAAAGGGAAAUAGCAGUUG
gma-MIR1508a(iso4) AGAAAGGGAAAUAGCAGUUG
gma-MIR1508a(iso5) GAAAGGGAAAUAGCAGUUG

Gm16:32903737-32903831, 95nt, (+)
AAUUGCUAUCCAACUGCUAUUCCCAUUUCUAAACCUUGUUACACGAGCAUCUUGAUCAAUGGUCUAGAAAGGGAAAUAGCAGUUGAGUGGUGCUU

Structure 1 Folding bases 1 To 95 of 10Sep29-16-03-59 Initial dG = -37.10

```
        10         20        30         40
AAUU    UC         -|    A      A   -   UUACA    C
    GCUA  CAACUGCUA UUCCC UUUCUA ACC UUG     CGAG \
```

```
    ||||  ||||||||| ||||| |||||| ||| |||     ||||
    UGGU  GUUGACGAU AAGGG AAAGAU UGG AAC     GUUC A
UUCG    GA         A^    -      C   U   UA---    U
    90        80         70        60            50
```

gma-MIR1508b UAGAAAGGGAAAUAGCAGUUG
gma-MIR1508b(iso1) AGAAAGGGAAAUAGCAGUUG
gma-MIR1508b(iso2) GAAAGGGAAAUAGCAGUUG
Gm09:28530168-28530271, 104nt, (+)
GUUGAAUCGCUACUCAACUGCUAUUUUCCUUUUUGAACCUUGUUACCUUGAGCAUCUUGAUCAAUUGUUUAGAAAGGGAAAUAGCAGUUGAGUAGUGCUUCAAC

Structure 1 Folding bases 1 To 104 of 10Sep29-16-02-07 Initial dG = -52.40

```
        10        20        30          40        50
      U                               C-|  UUACC UG  C
GUUGAA CGCUACUCAACUGCUAUUUUCCUUUUUGAAC  UUG     U  AG \
|||||| |||||||||||||||||||||||||||||||  |||     |  ||
CAACUU GUGAUGAGUUGACGAUAAAGGGAAAGAUUUG  AAC     A  UC A
      C                               UU^  U---- GU  U
  100        90        80        70            60
```

gma-MIR1509a UUAAUCAAGGAAAUCACGGUCG
Gm17:10099759-10099869, 111nt, (+)
CUGCAUCUUCUUAAUCAAGGAAAUCACGGUCGCGUGUGUGCCGGAAAGAAAGUGGCCUGUGAUCUCCGGUUUCUCUUUCUCGACCGUGUUUCCUUGGUUAACGAUAUGUGC

Structure 1 Folding bases 1 To 111 of 10Sep29-16-05-32 Initial dG = -42.00

```
         10         20        30           40        50
CU    CU-  -             U        CGUGUGU---      AAGAA-| U
  GCAU   UC UUAAUCAAGGAAA CACGGUCG          GCCGGA      AG G
  ||||   || ||||||||||||| ||||||||          ||||||      ||
  CGUG   AG AAUUGGUUCCUUU GUGCCAGC          UGGCCU      UC G
--    UAU  C             -        UCUUUCUCUU      CUAGUG^ C
 110      100        90         80        70        60
```

gma-MIR1509b(iso) UUAAUCAAGGAAAUCACGGUUG
Gm05:7774098-7774206, 109nt, (-)
CUGCAUCUUUUUAAUCAAGGAAAUCACGGUUGAGUGUGAAGGAGAGAAAGUGGCUUCAGAUUUCCGGGUUUUCCUUCUCCACUGUGUUUCCUUGGUUAAAGAUAUGUGC

Structure 1 Folding bases 1 To 109 of 10Sep29-16-06-55   Initial dG = -40.40

```
        10        20        30        40        50
CU|   C                U      U  GUGU         AAAG   CUUC
  GCAU UUUUUAAUCAAGGAAA CACGGU GA    GAAGGAGAG    UGG    A
  |||| |||||||||||||||| |||||| ||    |||||||||    |||
  UGUA AGAAAUUGGUUCCUUU GUGUCA CU    CUUCCUUUU    GCC    G
CG^   U                -      C  ----         GG--   UUUA
       100        90         80            70          60
```

gma-MIR1510a(iso1) UGUUGUUUUACCUAUUCCACCC
gma-MIR1510a(iso2) UGUUGUUUUACCUAUUCCACC
gma-MIR1510a(iso3) UGUUGUUUUACCUAUUCCAC
gma-MIR1510a(iso4) UGUUGUUUUACCUAUUCCA
gma-MIR1510a(iso5) UUGUUUUACCUAUUCCACCCAUU
gma-MIR1510a(iso6) UUGUUUUACCUAUUCCACCCAU
Gm16:31518908-31519000, 93nt, (+)
UUAUGGAACUGGAGGGAUAGGUAAAACAAUGACUGCUGUAUAAGUAAUUGUUAUAGUUAGUUGUUGUUUUACCUAUUCCACCCAUUCCAUGUA

Structure 1 Folding bases 1 To 93 of 10Sep29-16-12-02   Initial dG = -46.30

```
         10        20        30          40
U-       CU  A                UG   -     -|   G
  UAUGGAA  GG GGGAUAGGUAAAACAA  ACU GCUGU AUAA U
  |||||||  || ||||||||||||||||  ||| ||||| ||||
  GUACCUU  CC CCUUAUCCAUUUUGUU  UGA UGAUA UGUU A
AU       AC  A                GU   U     U^   A
  90        80        70        60        50
```

gma-MIR1510b-5p AGGGAUAGGUAAAACAACUACU
gma-MIR1510b-5p(iso1) GGGAUAGGUAAAACAACUAC
gma-MIR1510b-5p(iso2) GAUAGGUAAAACAACUACU
gma-MIR1510b-5p(iso3) AGGGAUAGGUAAAACAACUAC
gma-MIR1510b-5p(iso4) AGGGAUAGGUAAAACAACUA

gma-MIR1510b UGUUGUUUUACCUAUUCCACC
gma-MIR1510b-3p(iso1) UGUUGUUUUACCUAUUCCACCA
gma-MIR1510b-3p(iso2) UGUUGUUUUACCUAUUCCAC
gma-MIR1510b-3p(iso3) UGUUGUUUUACCUAUUCCA
Gm02:6599299-6599392, 94nt, (+)
UUUAUGGAAGUGGAGGGAUAGGUAAAACAACUACUUCUGUAAAAGUAAUUGUUAUAGUUAGUUGUUGUUUUACCUAUUCCACCAAUUCCAUCUA

Structure 1 Folding bases 1 To 94 of 10Sep29-16-09-45 Initial dG = -43.30

```
        10        20        30         40
UUU      G   A                 U   U-      -| G
   AUGGAA UGG GGGAUAGGUAAAACAAC ACU  CUGUAA AA U
   |||||| ||| ||||||||||||||||| |||  |||||| ||
   UACCUU ACC CCUUAUCCAUUUUGUUG UGA  GAUAUU UU A
AUC      A   A                 U   UU      G^ A
   90        80        70        60        50
```

gma-MIR1511(iso) AACCAGGCUCUGAUACCAUGG
Gm18:21161236-21161334, 99nt, (+)
UCAGCCGUGGUAUCAGGUCCUGCUUCAUCAAGUGGUCUUGUGUUCAAAUCCAGCCUCAAGCACAUGGUUAACCAGGCUCUGAUACCAUGGUGAAUAUAA

Structure 1 Folding bases 1 To 99 of 10Sep29-16-13-47 Initial dG = -38.60

```
              10        20        30        40
------|  G            GU    C  C    A   GU    U-   CAA
      UCA CCGUGGUAUCAG  CCUG UU AUCA GUG  CUUG  GUU   \
      ||| ||||||||||||  |||| || |||| |||  ||||  |||
      AGU GGUACCAUAGUC  GGAC AA UGGU CAC  GAAC  CGA   A
AAUAUA^  -            UC    C  U    A   --    UC   CCU
         90        80        70          60        50
```

gma-MIR1512b UAACUGGAAAUUCUUAAAGCAU
Gm02:8618692-8618781, 89nt,(-)
UUGAUACCUAACUGGAAAUUCUUAAAGCAUUCCCAUCAUCAAAAUGAUGUGAAAAUUAGGAAUGCUUUAAGAAUUUCAGUUAUGCAUCA

Structure 1 Folding bases 1 To 89 of 10Sep29-16-15-43 Initial dG = -35.30

```
        10        20        30                 40
U    ACC     G                   ---------|     C
 UGAU   UAACU GAAAUUCUUAAAGCAUUCC         CAUCAU A
 ||||   ||||| |||||||||||||||||||         ||||||
 ACUA   AUUGA CUUUAAGAAUUUCGUAAGG         GUAGUA A
```

```
-    CGU     -                   AUUAAAAGU^     A
         80         70        60        50
```

gma-MIR1513a UGAGAGAAAGCCAUGACUUAC
Gm07:43245809-43245901, 93nt,(+)
GGAUCAGAUAUGAGAGAAAGCCAUGACUUACACACACAUUGAAAUCUUAGUUUUAAAUGUGUAUAAGUCAUGGUUUUCUCUCAUAUCUAAUCC

Structure 1 Folding bases 1 To 93 of 10Sep29-16-21-09 Initial dG = -53.30

```
        10        20        30        40
|   C                         C     CA       C
GGAU AGAUAUGAGAGAAAGCCAUGACUUA ACACA  UUGAAAU U
|||| ||||||||||||||||||||||||| |||||  |||||||
CCUA UCUAUACUCUCUUUUGGUACUGAAU UGUGU  AAUUUUG U
^   A                         A     A-       A
  90        80        70        60         50
```

gma-MIR1513b UAUGAGAGAAAGCCAUGAC
Gm17:1401433-1401523, 91nt, (-)
GAUCUGAUAUGAGAGAAAGCCAUGACUUACACACGCAUUGAAAUCUAAGUUAAAUAUGUGUGUAAGUCAUGGCAUUAUCUCAUAUCUAAUC

Structure 1 Folding bases 1 To 91 of 10Sep29-16-18-55 Initial dG = -43.80

```
        10        20        30        40
|  CU       G  A                C   GAAAUC
GAU  GAUAUGAGA AA GCCAUGACUUACACACG AUU      \
|||  ||||||||| || ||||||||||||||||| |||
CUA  CUAUACUCU UU CGGUACUGAAUGUGUGU UAA      U
^  AU       A  A                A   AUUGAA
90        80        70        60        50
```

gma-MIR1513c AAAGCCAUGACUUACACACGC
Gm20:223679-223766, 88nt, (-)
GUUUCUAUGCGUUUGUAAAUCAUGACUUUCUCUUGUAUCUCAUCUGAGUAUAUGAGAGAAAGCCAUGACUUACACACGCAUAUGAAAC

Structure 1 Folding bases 1 To 88 of 10Sep29-16-17-18 Initial dG = -44.70

```
         10        20        30         40
    -       U     A     A        UG   -|  A
```

```
GUUUC UAUGCGU UGUAA UCAUG CUUUCUCU  UAU CUC U
||||| ||||||| ||||| ||||| ||||||||  ||| |||
CAAAG AUACGCA ACAUU AGUAC GAAAGAGA  AUA GAG C
     U       C     C     C        GU    U^   U
       80        70        60        50
```

gma-MIR1514a(iso) UUCAUUUUUAAAAUAGGCAUUG

Gm07:43175810-43175908, 99nt, (-)

CUUUGCUAUUUUCAUUUUUAAAAUAGGCAUUGGGGUCCCUUCUUGUUCCUCCUUUUCCUUUCCUAUCCCAAUGCCUAUUUUAAAAUGAAAACAACGAUA

Structure 1 Folding bases 1 To 99 of 10Sep29-16-22-57 Initial dG = -31.80

```
        10        20        30        40        50
CU|  CUA          U                UCCCUUCUUGUUCC
  UUG   UUUUCAUUUU AAAAUAGGCAUUGGGG              U
  |||   |||||||||| ||||||||||||||||
  AGC   AAAAGUAAAA UUUUAUCCGUAACCCU              C
AU^  AAC          -                AUCCUUUCCUUUUC
        90         80        70        60
```

gma-MIR1523(iso) UAUGGGAUAAAUGUGAGCUC

Gm02:12253303-12253397, 95nt, (-)

AGGACCCAUUAUGGGAUAAAUGUGAGCUCAGGAGCGAUGAAUAAAUCCUUUCAUCACUCCUCAUUACUCCCGAGCUCACAUUUAUUUCAAAAUGA

Structure 1 Folding bases 1 To 95 of 10Sep29-16-24-01InitialdG = -42.80

```
        10        20                  30        40
AGGACC    A  GG              ----------|    C      UAA
      CAUU UG  AUAAAUGUGAGCUC          AGGAG GAUGAA   A
      |||| ||  ||||||||||||||          ||||| ||||||
      GUAA AC  UAUUUACACUCGAG          UCCUC CUACUU   U
A-----    A  UU              CCCUCAUUAC^    A      UCC
         90        80        70        60        50
```

gma-MIR2109a-5p (iso1) UGCGAGUGUCUUCGCCUCUGA
gma-MIR2109a-3p GGAGGCGUAGAUACUCACACCU
gma-MIR2109a-3p (iso1) GGAGGCGUAGAUACUCACACC
gma-MIR2109a-3p (iso2) GGAGGCGUAGAUACUCACAC
gma-MIR2109a-3p (iso3) AGGCGUAGAUACUCACACC

Gm04:28532441-28532537, 97nt, (-)

AGCCAGUGAAAUCGGUGCGAGUGUCUUCGCCUCUGAGAGAGAUACUAUGAGAUCUCAAGCCUCGGAGGCGUAGAUACUCACACCUCUUUUUCUGGCU

Structure 1 Folding bases 1 To 97 of 10Sep29-16-25-45 Initial dG = -54.50

```
        10        20        30           40
      U    UC    C        U          A---|    ACU
AGCCAG GAAA  GGUG GAGUGUCU CGCCUCUGAG    GAGAU   A
|||||| ||||  |||| |||||||| ||||||||||    |||||
UCGGUC UUUU  CCAC CUCAUAGA GCGGAGGCUC    CUCUA   U
      U    CU    A        U          CGAA^    GAG
      90        80        70        60        50
```

gma-MIR4345(iso1) AGACGGAACUUACAAAGAU
gma-MIR4345(iso2) ACUUACAAAGAUUGUUCAGGUAGA
Gm14:49069099-49069193, 95nt, (+)
GGAAGCUAAGACGGAACUUACAAAGAUUGUUCAGGUAGACAUUUGAGAAUAAAUGUCUGUCUUGAACAAUCUUUUUAAGUUUCGUCUUAACUUUC

Structure 1 Folding bases 1 To 95 of 10Sep29-16-27-25 Initial dG = -51.80

```
        10        20        30         40
     C              C              -|          A
GGAAG UAAGACGGAACUUA AAAGAUUGUUCAGG UAGACAUUUG G
||||| |||||||||||||| |||||||||||||| ||||||||||
CUUUC AUUCUGCUUUGAAU UUUCUAACAAGUUC GUCUGUAAAU A
     A              U              U^          A
    90        80        70        60        50
```

gma-MIR4376-3p AGCAUCAUAUCUCCUGCAUAG
Gm13:40845925-40846034, 110nt, (+)
AAGGUUUGCUACGCAGGAGAGAUGACGCUGUCCCUUGCACCCAUCCUAGCUUCCCUUGAGUAGGUAAGAGCAAGGCCAGCCAGCAUCAUAUCUCCUGCAUAGUAAACCUU

Structure 1 Folding bases 1 To 110 of 10Sep29-16-33-31 Initial dG = -52.40

```
        10        20        30           40        50
           C        G    C    UC---|     ACCCAU    G   CC
AAGGUUUGCUA GCAGGAGA AUGA GCUG     CCUUGC      CCUA CUU  \
||||||||||| |||||||| |||| ||||     ||||||      |||| |||
UUCCAAAUGAU CGUCCUCU UACU CGAC     GGAACG      GGAU GAG  C
           A        A    A    CGACC^     AGAAU-    -   UU
.       100        90        80        70          60
```

gma-MIR4413b UAAGAGAAUUGUAAGUCACU
Gm13:5170460-5170527, 68nt, (+)
CAUCAAUAAGAGAAUUGUAAGUCACUUGAUUAGGAAAUUUUACGGAGACUUACAAUUCCGUAUUGAUG

Structure 1 Folding bases 1 To 68 of 10Sep29-16-35-09 Initial dG = -23.00

```
        10        20          30
|       AGA            A  -   UUAG
CAUCAAUA   GAAUUGUAAGUC CU UGA    G
```

```
||||||||   |||||||||||| || |||
GUAGUUAU   CUUAACAUUCAG GG AUU    A
^       GC-            A  C   UUAA
       60         50        40
```

gma-MIR4413a AAGAGAAUUGUAAGUCACUG
gma-MIR4413a(iso1) UAAGAGAAUUGUAAGUCACUG
gma-MIR4413a(iso2) UAAGAGAAUUGUAAGUCACU
Gm19:1788518-1788620, 103nt, (+)
GCAUCCUCAUCAAUAAGAGAAUUGUAAGUCACUGUAUUAAUUAGGAACUGUUGAUUAGAUGCAUGAUACAGUGACUUACAAUUCUCUUAUUAAUGAUUUGUGC

Structure 1 Folding bases 1 To 103 of 10Sep29-16-36-36 Initial dG = -45.40

```
         10        20        30        40        50
    CC-|   C                            AUUAGGAA   UU
GCAU   UCAU AAUAAGAGAAUUGUAAGUCACUGUAUUA        CUG  \
||||   |||| ||||||||||||||||||||||||||||        |||
CGUG   AGUA UUAUUCUCUUAACAUUCAGUGACAUAGU        GAU  G
    UUU^   A                            ACGUA---   UA
 100      90        80        70        60
```

gma-MIR4415b UUGAUUCUCAUCACAACAUGG
Gm08:23142767-23142922, 156nt, (+)
GGCUGCAUCAAGUUGUGAUGGGAAUCAAUGGCAGCAAUCACGCCAAGAAAAUGAAAUCCCAUUAUCUUCUCACAGUAUAUCAUUAAUUAGGCUAACUGGUGUUGACGGAUAUAAAUCCAUUAC
UGCCAUUGAUUCUCAUCACAACAUGGUCCAGUC

Structure 1 Folding bases 1 To 156 of 10Sep29-16-37-59 Initial dG = -63.90

```
       10        20        30           40          50
     C    A                       .-CAA  -      .-A| A    AA
GGCUG AUCA GUUGUGAUGGGAAUCAAUGGCAG     UC ACGCCA   GA AAUG  A
||||| |||| |||||||||||||||||||||||     || ||||||   || ||||
CUGAC UGGU CAACACUACUCUUAGUUACCGUC     AG UGUGGU   CU UUAC  U
     C    A                       \ ---  U      \ -^ A    CC
    150       140       130                 100         60
```

```
  70        80
UUCUCAC   A  UCA
       AGU UA   U
       ||| ||
       UCG AU   U
CAA----   G  UAA
         90
```

```
     110
C---    A
    GGAU U
    ||||
    CCUA A
AUUA    A
120
```

gma-MIR4415a-3p UUGAUUCUCAUCACAACAUGG

Gm18:60474198-60474369, 172nt, (+)

CUGCAGCAAGUUGUGAUGAGAAUCAAUGGCAAGCAGUGACACCAAGAAAAAAAAAUCCCAUUAUAAACAUUUUUUCCCCUCAAUCUUCUUCUCAUAGUAUAUCAUUAGGCUAACUGGUGUUGA
AGGAUAUAAAUCCACAACUGCCAUUGAUUCUCAUCACAACAUGGUCCAG

Structure 1 Folding bases 1 To 172 of 10Sep29-16-39-07 Initial dG = -55.90

```
        10        20        30          40          50        60
   CAG  A                      .-AGCAGU       .-A|      AAAUCCCA
CUG   CA GUUGUGAUGAGAAUCAAUGGCA        GACACCA   GAAAAAA        \
|||   || ||||||||||||||||||||||        |||||||   |||||||
GAC   GU CAACACUACUCUUAGUUACCGU        UUGUGGU   CUUUUUU        U
   CUG  A                      \ ------       \ -^      ACAAAUAU
170       160       150               120             70
```

```
  80        90       100
CCCUCAAUCUUCUUCUCA    A  U
                  UAGU UA C
                  |||| ||
                  AUCG AU A
CA----------------    G  U
                  110
```

```
         130
 GAA--    A
      GGAU U
      ||||
      CCUA A
 CAACA    A
140
```
