# Supplementary material for: Identification of novel soybean microRNAs involved in abiotic and biotic stresses
Source: BMC Genomics. 2011 Jun 10;12:307. doi: 10.1186/1471-2164-12-307 (PMC3141666; doi:10.1186/1471-2164-12-307)
Supplement: Additional file 2 — Identified targets of known conserved plant miRNAs families. a The Data from Phytozome version 6.0. b Pairing obtained in psRNATarget Server: "|" indicates a Watson-Crick base pairing; ":" is a G:U base pairing, and "-"indicates a mismatch. [file 1471-2164-12-307-S2.PDF]

Additional file 2. Predicted *Glycine max* mRNA targets for conserved miRNAs.

<table>
<tr><th>miRNA ID</th><th>Locus target[a]</th><th>Target description[a]</th><th>miRNA/mRNA pairing[b]</th></tr>
<tr><td>gma-MIR156d,h,i,k,l</td><td>Glyma02g13370</td><td>SBP-domain protein</td><td>|||||||||-||||||||||:</td></tr>
<tr><td></td><td>Glyma02g30670</td><td>SBP-domain protein</td><td>|||||||||-|||||||||||</td></tr>
<tr><td></td><td>Glyma03g27200</td><td>SBP-domain protein</td><td>-|||||||||-||||||||||</td></tr>
<tr><td></td><td>Glyma03g29900</td><td>SBP-domain protein</td><td>|||||||||-|||||||||||</td></tr>
<tr><td></td><td>Glyma04g37390</td><td>SBP-domain protein</td><td>-|||||||||-||||||||||</td></tr>
<tr><td></td><td>Glyma05g00200</td><td>SBP-domain protein</td><td>-|||||||||-||||||||||</td></tr>
<tr><td></td><td>Glyma05g38180</td><td>SBP-domain protein</td><td>-|||||||||-||||||||||</td></tr>
<tr><td></td><td>Glyma06g17700</td><td>SBP-domain protein</td><td>-|||||||||-||||||||||</td></tr>
<tr><td></td><td>Glyma07g31880</td><td>SBP-domain protein</td><td>-||||||||||||||||||||</td></tr>
<tr><td></td><td>Glyma08g01450</td><td>SBP-domain protein</td><td>-|||||||||-||||||||||</td></tr>
<tr><td></td><td>Glyma11g36980</td><td>SBP-domain protein</td><td>|||||||||-|||||||||||</td></tr>
<tr><td></td><td>Glyma13g24590</td><td>SBP-domain protein</td><td>-||||||||||||||||||||</td></tr>
<tr><td></td><td>Glyma13g35000</td><td>SBP-domain protein</td><td>-|||||-||-|||||||||||</td></tr>
<tr><td></td><td>Glyma17g08840</td><td>SBP-domain protein</td><td>-|||||||||-||||||||||</td></tr>
<tr><td></td><td>Glyma18g36960</td><td>SBP-domain protein</td><td>|||||||||-|||||||||||</td></tr>
<tr><td></td><td>Glyma19g32800</td><td>SBP-domain protein</td><td>|||||||||-|||||||||||</td></tr>
<tr><td>gma-MIR156j,m,n</td><td>Glyma02g13370</td><td>SBP-domain protein</td><td>||||||-|||||||||||||:</td></tr>
<tr><td></td><td>Glyma02g30670</td><td>SBP-domain protein</td><td>||||||-||||||||||||||</td></tr>
<tr><td></td><td>Glyma03g27200</td><td>SBP-domain protein</td><td>||||||-|||||||||||||-</td></tr>
<tr><td></td><td>Glyma03g29900</td><td>SBP-domain protein</td><td>||||||-||||||||||||||</td></tr>
<tr><td></td><td>Glyma04g37390</td><td>SBP-domain protein</td><td>||||||-|||||||||||||-</td></tr>
<tr><td></td><td>Glyma05g00200</td><td>SBP-domain protein</td><td>||||||-|||||||||||||-</td></tr>
<tr><td></td><td>Glyma05g38180</td><td>SBP-domain protein</td><td>||||||-|||||||||||||-</td></tr>
<tr><td></td><td>Glyma06g17700</td><td>SBP-domain protein</td><td>||||||-|||||||||||||-</td></tr>
<tr><td></td><td>Glyma07g31880</td><td>SBP-domain protein</td><td>-|||||-||-|||||||||||</td></tr>
<tr><td></td><td>Glyma08g01450</td><td>SBP-domain protein</td><td>||||||-|||||||||||||-</td></tr>
<tr><td></td><td>Glyma11g36980</td><td>SBP-domain protein</td><td>||||||-||||||||||||||</td></tr>
<tr><td></td><td>Glyma13g24590</td><td>SBP-domain protein</td><td>-|||||-||-|||||||||||</td></tr>
<tr><td></td><td>Glyma13g35000</td><td>SBP-domain protein</td><td>-|||||-||||||||||||||</td></tr>
<tr><td></td><td>Glyma17g08840</td><td>SBP-domain protein</td><td>||||||-|||||||||||||-</td></tr>
</table>

<table>
<tr><td></td><td>Glyma18g36960</td><td>SBP-domain protein</td><td>||||||-||||||||||||||</td></tr>
<tr><td></td><td>Glyma19g32800</td><td>SBP-domain protein</td><td>||||||-||||||||||||||</td></tr>
<tr><td>gma-MIR159a-3p</td><td>Glyma04g10930</td><td>plant organelle RNA recognition domain</td><td>|:||||||:|||||||::||:</td></tr>
<tr><td></td><td>Glyma06g36770</td><td>ankyrin repeat-containig</td><td>|-|||:|:||||||||:|||:</td></tr>
<tr><td>gma-MIR159b-3p</td><td>Glyma13g04030</td><td>MYB-related protein</td><td>|||||||||||||||||||||</td></tr>
<tr><td></td><td>Glyma13g25720</td><td>MYB-related protein</td><td>|||||||||||||||||||||</td></tr>
<tr><td></td><td>Glyma15g33630</td><td>SF3 transcription factor X1-Like</td><td>|||||||||||||||||||||</td></tr>
<tr><td></td><td>Glyma19g37570</td><td>protein tyrosine kinase</td><td>-|||||||||||||:|-||||</td></tr>
<tr><td></td><td>Glyma20g11040</td><td>MYB-related protein</td><td>|||||||||||||||||||||</td></tr>
<tr><td>gma-MIR159e-5p</td><td>Glyma15g37290</td><td>LRR-containing protein</td><td>||-||||||-|||||:||||</td></tr>
<tr><td></td><td>Glyma14g40230</td><td>hydrolase activity</td><td>|:|||:|-|:|||||||||:</td></tr>
<tr><td></td><td>Glyma17g37900</td><td>hydrolase activity</td><td>|:|||:|-|:|||||||||:</td></tr>
<tr><td></td><td>Glyma18g01780</td><td>hydrolase activity</td><td>|:||||||||:||||-|||:</td></tr>
<tr><td>gma-MIR162a,b,c</td><td>Glyma07g06590</td><td>60S ribosomal protein L5</td><td>||-||||||||||||-||||:</td></tr>
<tr><td>gma-MIR166a-3p</td><td>Glyma05g06070</td><td>Myb-like DNA-binding domain</td><td>|||:|||--||||||||||:|</td></tr>
<tr><td>gma-MIR166c-5p</td><td>Glyma09g34720</td><td>predicted calmodulin-binding protein</td><td>|||||||||-||-|::||||</td></tr>
<tr><td>gma-MIR166d-5p</td><td>Glyma17g21240</td><td>LRR-containing protein</td><td>|:||||-|:|||||::|||||</td></tr>
<tr><td>gma-MIR168a,b</td><td>Glyma10g30650</td><td>Calcium-responsive transcription coactivator</td><td>||||||-:||||-||:|||||</td></tr>
<tr><td></td><td>Glyma11g04200</td><td>protein tyrosine kinase</td><td>||:||||-||||||||||-||</td></tr>
<tr><td>gma-MIR169f-5p</td><td>Glyma14g01080</td><td>PPR-containig protein</td><td>--|||:|||:|||||||||||</td></tr>
<tr><td>gma-MIR169k</td><td>Glyma09g07960</td><td>transcription factor NF-Y alpha-related</td><td>|-|||||||||:||||||||-</td></tr>
<tr><td></td><td>Glyma13g16770</td><td>transcription factor NF-Y alpha-related</td><td>|:|||||||||:||||||||-</td></tr>
<tr><td></td><td>Glyma15g18970</td><td>transcription factor NF-Y alpha-related</td><td>|-|||||||||:||||||||-</td></tr>
<tr><td></td><td>Glyma17g05920</td><td>transcription factor NF-Y alpha-related</td><td>|:|||||||||:||||||||-</td></tr>
<tr><td></td><td>Glyma19g35620</td><td>ADP-ribosylation factor GTPase activator</td><td>::||:||||:|:|||||||||</td></tr>
<tr><td>gma-MIR170</td><td>Glyma08g41980</td><td>iron/ascorbate family oxidoreductases</td><td>|:||:||||||:|||||||-|</td></tr>
<tr><td></td><td>Glyma18g13610</td><td>iron/ascorbate family oxidoreductases</td><td>|:||:||||||:|||||||-|</td></tr>
<tr><td>gma-MIR171d</td><td>Glyma10g40780</td><td>serine/threonine protein kinase</td><td>:|||:|||||||::||:||||</td></tr>
<tr><td></td><td>Glyma11g17490</td><td>GRAS family transcription factor</td><td>||-|||||||||:||||||||</td></tr>
<tr><td></td><td>Glyma20g26510</td><td>serine/threonine protein kinase</td><td>:|||:|||||||::||:||||</td></tr>
<tr><td>gma-MIR172b-5p</td><td>Glyma01g39520</td><td>transcription factor activity</td><td>|-|||||-||||||||||:|</td></tr>
<tr><td></td><td>Glyma03g33470</td><td>transcription factor activity</td><td>|-|||||-||||||||||:|</td></tr>
<tr><td></td><td>Glyma05g09400</td><td>protein kinase C activation</td><td>-||-||||||||||||:||:</td></tr>
</table>

<table>
<tr><td></td><td>Glyma11g05720</td><td>transcription factor activity</td><td>|-|||||-||||||||||:|</td></tr>
<tr><td></td><td>Glyma11g10790</td><td>RNA-binding protein</td><td>|||:||:|||:|||||||:-</td></tr>
<tr><td></td><td>Glyma14g01950</td><td>A2L zinc ribbon domain</td><td>||||||-|||||||||:-||</td></tr>
<tr><td></td><td>Glyma19g36200</td><td>transcription factor activity</td><td>|-|||||-||||||||||:|</td></tr>
<tr><td>gma-MIR172c</td><td>Glyma01g39520</td><td>AP2 domain-containing transcription factor</td><td>||||||||||||||:||||||</td></tr>
<tr><td></td><td>Glyma03g33470</td><td>AP2 domain-containing transcription factor</td><td>||||||||||||||:||||||</td></tr>
<tr><td></td><td>Glyma05g18170</td><td>AP2 domain-containing transcription factor</td><td>||||||||||||||||-||||</td></tr>
<tr><td></td><td>Glyma05g31790</td><td>GTPase Rab2, small G protein superfamily</td><td>-||||-|||||-|||||||||</td></tr>
<tr><td></td><td>Glyma08g15040</td><td>GTPase Rab2, small G protein superfamily</td><td>-||||-|||||-|||||||||</td></tr>
<tr><td></td><td>Glyma11g05720</td><td>AP2 domain-containing transcription factor</td><td>||||||||||||||:||||||</td></tr>
<tr><td></td><td>Glyma11g15650</td><td>AP2 domain-containing transcription factor</td><td>||||||||||||||:|||||:</td></tr>
<tr><td></td><td>Glyma12g07800</td><td>AP2 domain-containing transcription factor</td><td>||||||||||||||:|||||:</td></tr>
<tr><td></td><td>Glyma13g40470</td><td>AP2 domain-containing transcription factor</td><td>||||||||||||||:|||||:</td></tr>
<tr><td></td><td>Glyma15g04930</td><td>AP2 domain-containing transcription factor</td><td>||||||||||||||:|||||:</td></tr>
<tr><td></td><td>Glyma17g18640</td><td>AP2 domain-containing transcription factor</td><td>||||||||||||||||-||||</td></tr>
<tr><td></td><td>Glyma19g36200</td><td>AP2 domain-containing transcription factor</td><td>||||||||||||||:||||||</td></tr>
<tr><td>gma-MIR172g</td><td>Glyma06g15630</td><td>ubiquitin-protein ligase activity</td><td>|||||||||-|||||-:|||</td></tr>
<tr><td></td><td>Glyma10g27970</td><td>ATP binding cassette protein</td><td>||||||:||||--|||||||</td></tr>
<tr><td>gma-MIR172h-3p</td><td>Glyma01g39520</td><td>AP2 domain-containing transcription factor</td><td>-|||||||||||||:|||||</td></tr>
<tr><td></td><td>Glyma03g33470</td><td>AP2 domain-containing transcription factor</td><td>-|||||||||||||:|||||</td></tr>
<tr><td></td><td>Glyma11g05720</td><td>AP2 domain-containing transcription factor</td><td>-|||||||||||||:|||||</td></tr>
<tr><td></td><td>Glyma11g15650</td><td>AP2 domain-containing transcription factor</td><td>-|||||||||||||:||||||</td></tr>
<tr><td></td><td>Glyma12g07800</td><td>AP2 domain-containing transcription factor</td><td>-|||||||||||||:||||||</td></tr>
<tr><td></td><td>Glyma13g40470</td><td>AP2 domain-containing transcription factor</td><td>-|||||||||||||:||||||</td></tr>
<tr><td></td><td>Glyma15g04930</td><td>AP2 domain-containing transcription factor</td><td>-|||||||||||||:||||||</td></tr>
<tr><td></td><td>Glyma19g36200</td><td>AP2 domain-containing transcription factor</td><td>-|||||||||||||:|||||</td></tr>
<tr><td>gma-MIR172h-5p</td><td>Glyma06g13450</td><td>putative ATP-dependent Clp-type protease</td><td>||||||--||||||||||||</td></tr>
<tr><td></td><td>Glyma10g08730</td><td>nitrate, fromate, iron dehydrogenase</td><td>||-|:-|||||:|||||||||</td></tr>
<tr><td></td><td>Glyma10g30570</td><td>targeting protein for Xklp2</td><td>||||::|||||:||:||:|||</td></tr>
<tr><td></td><td>Glyma11g05580</td><td>GTP-binding ADP-ribosylation factor</td><td>-|||::||||||||||||||-</td></tr>
<tr><td></td><td>Glyma11g06830</td><td>ubiquitin-protein ligase</td><td>--||||||-||||||||||||</td></tr>
<tr><td></td><td>Glyma15g12600</td><td>protease inhibitor</td><td>||-|:|||||||-|:||||||</td></tr>
<tr><td>gma-MIR2118b-5p</td><td>Glyma02g36360</td><td>replication termination factor 2</td><td>|||||-|||:||||||:||-</td></tr>
</table>

<table>
<tr><th></th><th></th><th></th><th></th></tr>
<tr><td></td><td>Glyma17g08320</td><td>replication termination factor 2</td><td>|||||-|||:||||||:||-</td></tr>
<tr><td>gma-MIR395a,b,c</td><td>Glyma06g11150</td><td>sulfate transporter</td><td>||||||--||||||||||||</td></tr>
<tr><td></td><td>Glyma10g38760</td><td>ATP sulfurylase</td><td>|||||||:||||||:|||||-</td></tr>
<tr><td></td><td>Glyma17g10050</td><td>gibberellin regulated protein</td><td>|-|||||||||::-|||||||</td></tr>
<tr><td></td><td>Glyma18g02240</td><td>sulfate transporter</td><td>-|||||:|||||||||||||-</td></tr>
<tr><td></td><td>Glyma18g03110</td><td>phospholipase C-related</td><td>|-|||:|:|||||:|:|||||</td></tr>
<tr><td></td><td>Glyma20g28980</td><td>ATP sulfurylase</td><td>|||||||:||||||:|||||-</td></tr>
<tr><td>gma-MIR396a-3p</td><td>Glyma11g12580</td><td>adenylate cyclase-associated protein</td><td>:||:|||::|-|||||||||</td></tr>
<tr><td></td><td>Glyma12g04790</td><td>adenylate cyclase-associated protein</td><td>:||:|||::|-|||||||||</td></tr>
<tr><td></td><td>Glyma16g00260</td><td>core-2/I-branching enzyme</td><td>||||||-|||||||:|||||</td></tr>
<tr><td>gma-MIR396a-5p</td><td>Glyma01g34650</td><td>growth-regulating transcription factor</td><td>-||||||||||||-|||||||</td></tr>
<tr><td></td><td>Glyma01g44470</td><td>growth-regulating transcription factor</td><td>-||||||||||||-|||||||</td></tr>
<tr><td></td><td>Glyma03g02500</td><td>growth-regulating transcription factor</td><td>-||||||||||||-|||||||</td></tr>
<tr><td></td><td>Glyma03g35010</td><td>growth-regulating transcription factor</td><td>-||||||||||||-|||||||</td></tr>
<tr><td></td><td>Glyma04g40880</td><td>growth-regulating transcription factor</td><td>-||||||||||||-|||||||</td></tr>
<tr><td></td><td>Glyma06g13960</td><td>growth-regulating transcription factor</td><td>-||||||||||||-|||||||</td></tr>
<tr><td></td><td>Glyma07g04290</td><td>growth-regulating transcription factor</td><td>-||||||||||||-|||||||</td></tr>
<tr><td></td><td>Glyma09g07990</td><td>growth-regulating transcription factor</td><td>-||||||||||||-|||||||</td></tr>
<tr><td></td><td>Glyma10g07790</td><td>growth-regulating transcription factor</td><td>-||||||||||||-|||||||</td></tr>
<tr><td></td><td>Glyma11g01060</td><td>growth-regulating transcription factor</td><td>-||||||||||||-|||||||</td></tr>
<tr><td></td><td>Glyma11g11820</td><td>growth-regulating transcription factor</td><td>-||||||||||||-|||||||</td></tr>
<tr><td></td><td>Glyma12g01730</td><td>growth-regulating transcription factor</td><td>-||||||||||||-|||||||</td></tr>
<tr><td></td><td>Glyma13g16920</td><td>growth-regulating transcription factor</td><td>-||||||||||||-|||||||</td></tr>
<tr><td></td><td>Glyma13g21630</td><td>growth-regulating transcription factor</td><td>-||||||||||||-|||||||</td></tr>
<tr><td></td><td>Glyma14g10090</td><td>growth-regulating transcription factor</td><td>-||||||||||||-|||||||</td></tr>
<tr><td></td><td>Glyma15g19460</td><td>growth-regulating transcription factor</td><td>-||||||||||||-|||||||</td></tr>
<tr><td></td><td>Glyma16g00970</td><td>growth-regulating transcription factor</td><td>-||||||||||||-|||||||</td></tr>
<tr><td></td><td>Glyma17g05800</td><td>growth-regulating transcription factor</td><td>-||||||||||||-|||||||</td></tr>
<tr><td></td><td>Glyma17g35090</td><td>growth-regulating transcription factor</td><td>-||||||||||||-|||||||</td></tr>
<tr><td></td><td>Glyma19g37740</td><td>growth-regulating transcription factor</td><td>-||||||||||||-|||||||</td></tr>
<tr><td>gma-MIR396b-3p</td><td>Glyma02g01370</td><td>AMP dependent ligase/synthetase</td><td>|||:||||-||||||||||-|</td></tr>
<tr><td></td><td>Glyma13g12120</td><td>anticodon binding domain</td><td>|||:||||-||||||||||-|</td></tr>
<tr><td></td><td>Glyma16g03560</td><td>PPR-containing protein</td><td>|||:|||-:|||-||||||||</td></tr>
</table>

<table>
<tr><td>gma-MIR396b-5p</td><td>Glyma12g30730</td><td>stress responsive A/B Barrel Domain</td><td>|||||||||:|||-|||||-|</td></tr>
<tr><td></td><td>Glyma17g08020</td><td>heat shock protein 70KDa</td><td>|||-|::||||:||||||||:</td></tr>
<tr><td></td><td>Glyma18g01000</td><td>rubber elongation factor protein</td><td>||-||||:-||||:|||||||</td></tr>
<tr><td>gma-MIR396f</td><td>Glyma04g14970</td><td>drug transporter-related</td><td>||||||||-||:-:||||||</td></tr>
<tr><td></td><td>Glyma08g37250</td><td>SEL-1-Like proteins</td><td>:|-||-:|||||||||||||</td></tr>
<tr><td></td><td>Glyma18g01030</td><td>protein binding</td><td>:|-|||-|||||||||||||</td></tr>
<tr><td></td><td>Glyma18g47180</td><td>SEL-1-Like proteins</td><td>:|-||-:|||||||||||||</td></tr>
<tr><td>gma-MIR396h-3p</td><td>Glyma11g12580</td><td>adenylate cyclase-associated protein</td><td>:||:|||::|-||||||||||</td></tr>
<tr><td></td><td>Glyma12g04790</td><td>adenylate cyclase-associated protein</td><td>:||:|||::|-||||||||||</td></tr>
<tr><td></td><td>Glyma12g28570</td><td>core-2/I-branching enzyme</td><td>||||||-|||||||:|||||:</td></tr>
<tr><td></td><td>Glyma16g00260</td><td>Core-2/I-branching enzyme</td><td>||||||-|||||||:|||||:</td></tr>
<tr><td>gma-MIR397a,b</td><td>Glyma01g26750</td><td>multicopper oxidases</td><td>:|||||||||||||||||||</td></tr>
<tr><td></td><td>Glyma02g38990</td><td>multicopper oxidases</td><td>-||||||||||||||-|||||</td></tr>
<tr><td></td><td>Glyma03g14450</td><td>multicopper oxidases</td><td>:|||||:||||||||||||||</td></tr>
<tr><td></td><td>Glyma03g15800</td><td>multicopper oxidases</td><td>:|||||:||||||||||||||</td></tr>
<tr><td></td><td>Glyma07g16080</td><td>multicopper oxidases</td><td>-||||||||||||||:|||||</td></tr>
<tr><td></td><td>Glyma07g17170</td><td>multicopper oxidases</td><td>-||||||||||||:|||||-|</td></tr>
<tr><td></td><td>Glyma08g47380</td><td>multicopper oxidases</td><td>-||||||||||||||:|||||</td></tr>
<tr><td></td><td>Glyma11g07430</td><td>multicopper oxidases</td><td>||||||:|||||-||-|||||</td></tr>
<tr><td></td><td>Glyma11g14600</td><td>multicopper oxidases</td><td>-|||||:||||||||||||||</td></tr>
<tr><td></td><td>Glyma12g06480</td><td>multicopper oxidases</td><td>-|||||:||||||||||||||</td></tr>
<tr><td></td><td>Glyma14g37040</td><td>multicopper oxidases</td><td>-||||||||||||||-|||||</td></tr>
<tr><td></td><td>Glyma18g02690</td><td>multicopper oxidases</td><td>||||||:||||||||||||-|</td></tr>
<tr><td></td><td>Glyma18g38710</td><td>multicopper oxidases</td><td>-|||||:||||||||||||||</td></tr>
<tr><td></td><td>Glyma18g40070</td><td>multicopper oxidases</td><td>-||||||||||||||:|||||</td></tr>
<tr><td></td><td>Glyma18g41910</td><td>multicopper oxidases</td><td>-||||||||||||:|||||-|</td></tr>
<tr><td>gma-MIR408a</td><td>Glyma03g26060</td><td>plastocyanin-like domain</td><td>||:||||||||||||||||--</td></tr>
<tr><td></td><td>Glyma04g42120</td><td>plastocyanin-like domain</td><td>-:|||||||||||||||||||</td></tr>
<tr><td></td><td>Glyma06g12680</td><td>plastocyanin-like domain</td><td>-:|||||||||||||||||||</td></tr>
<tr><td></td><td>Glyma07g13840</td><td>plastocyanin-like domain</td><td>||:||||||||||||||||--</td></tr>
<tr><td>gma-MIR408b-5p</td><td>Glyma11g20520</td><td>transcription factor HEX</td><td>||||||||||:|-||||||-</td></tr>
<tr><td></td><td>Glyma12g08080</td><td>transcription factor HEX</td><td>||||||||||:|-||||||-</td></tr>
<tr><td>gma-MIR1508b</td><td>Glyma14g02680</td><td>Ca2+/calmodulin-dependent protein kinase</td><td>|||||-:||||||:||||:|:</td></tr>
</table>

<table>
<tr><td></td><td>Glyma16g28020</td><td>PPR-containig protein</td><td>|-|||||-|||||||||||||</td></tr>
<tr><td></td><td>Glyma19g43790</td><td>protein phosphatase</td><td>||:|||:||||||::|||||-</td></tr>
<tr><td>gma-MIR1510b</td><td>Glyma02g04750</td><td>ATP binding</td><td>|||||-||||||||:|||||-</td></tr>
<tr><td></td><td>Glyma04g39740</td><td>transmembrane receptor activity</td><td>|||||||||||-|||||||||</td></tr>
<tr><td></td><td>Glyma09g38390</td><td>oxidoreductase activity</td><td>-|||||||-||||-|||||||</td></tr>
<tr><td></td><td>Glyma13g25440</td><td>LRR-cointaing protein</td><td>|||||:||:|||||:||-|||</td></tr>
<tr><td></td><td>Glyma13g26230</td><td>LRR-cointaing protein</td><td>||-|||||:||||||||-|||</td></tr>
<tr><td></td><td>Glyma13g26250</td><td>LRR-cointaing protein</td><td>|||||:||:|||||:||-|||</td></tr>
<tr><td></td><td>Glyma16g22620</td><td>ATP binding</td><td>|||||-||||||||:|||||-</td></tr>
<tr><td></td><td>Glyma16g23800</td><td>LRR-cointaing protein</td><td>||-||:|||||-|||||||||</td></tr>
<tr><td></td><td>Glyma17g02100</td><td>F-box domain-containing protein</td><td>||||||||||||||||||-||</td></tr>
<tr><td></td><td>Glyma19g07660</td><td>LRR-cointaing protein</td><td>||||||:||||||||||:|||</td></tr>
<tr><td></td><td>Glyma19g07700</td><td>LRR-cointaing protein</td><td>|||||||||||-|||||:|||</td></tr>
<tr><td></td><td>Glyma20g26970</td><td>predicted E3 ubiquitin ligase</td><td>|||-||:||||||||:||::|</td></tr>
<tr><td>gma-MIR1512b</td><td>Glyma13g43710</td><td>CASC3/Barentsz eIF4AIII binding</td><td>|||:||-||||||||::|||||</td></tr>
<tr><td></td><td>Glyma15g01650</td><td>CASC3/Barentsz eIF4AIII binding</td><td>|||:||-||||||||::|||||</td></tr>
<tr><td>gma-MIR1513a</td><td>Glyma08g10360</td><td>F-box domain-containing protein</td><td>|-|||||||||||||:|||-|</td></tr>
<tr><td></td><td>Glyma08g27820</td><td>F-box domain-containing protein</td><td>|||||||||||||||||-|||</td></tr>
<tr><td></td><td>Glyma08g27850</td><td>F-box domain-containing protein</td><td>||||||||||||||||-||-|</td></tr>
<tr><td></td><td>Glyma08g27950</td><td>F-box domain-containing protein</td><td>|:||-||||||||||||||||</td></tr>
<tr><td></td><td>Glyma10g26670</td><td>F-box domain-containing protein</td><td>|-|||||||||||||:|||-|</td></tr>
<tr><td></td><td>Glyma18g52630</td><td>protein geranylgeranyltransferase</td><td>|||||||||||||||||||-|</td></tr>
<tr><td>gma-MIR1513b</td><td>Glyma08g10360</td><td>F-box domain-containing protein</td><td>|||||||||||:|||-|||</td></tr>
<tr><td></td><td>Glyma08g27950</td><td>F-box domain-containing protein</td><td>|||||||||||||||-||-</td></tr>
<tr><td></td><td>Glyma15g18380</td><td>transcription factor activity</td><td>-||||||||||-|||:||:</td></tr>
<tr><td></td><td>Glyma13g00200</td><td>transcription factor activity</td><td>-||||||||||-|||:||:</td></tr>
<tr><td></td><td>Glyma17g06290</td><td>transcription factor activity</td><td>-||||||||||-|||:||:</td></tr>
<tr><td>gma-MIR1513c</td><td>Glyma02g16510</td><td>F-box domain-containing protein</td><td>|:||-|||||-||||||||||</td></tr>
<tr><td></td><td>Glyma08g10360</td><td>F-box domain-containing protein</td><td>|||||||-|||||||||||||</td></tr>
<tr><td></td><td>Glyma08g27850</td><td>F-box domain-containing protein</td><td>|:||-||||||||||||||||</td></tr>
<tr><td></td><td>Glyma08g27950</td><td>F-box domain-containing protein</td><td>|:||-||||||||||||||||</td></tr>
<tr><td></td><td>Glyma17g02100</td><td>F-box domain-containing protein</td><td>-:||-||||||||||||||||</td></tr>
<tr><td>gma-MIR4376a-3p</td><td>Glyma02g07160</td><td>Predicted membrane protein</td><td>||-||::|-||||||||||||</td></tr>
</table>

<table>
<tr><td></td><td>Glyma02g15650</td><td>inositol 5-phosphatase</td><td>||:-|:|-|||||||||||||</td></tr>
<tr><td></td><td>Glyma05g01180</td><td>ribosomal protein S4</td><td>||:|||--|||||||||||:|</td></tr>
<tr><td></td><td>Glyma07g32780</td><td>inositol 5-phosphatase</td><td>||:-|:|-|||||||||||||</td></tr>
<tr><td></td><td>Glyma16g26110</td><td>predicted membrane protein</td><td>||-||::|-||||||||||||</td></tr>
<tr><td>gma-MIR4413a</td><td>Glyma09g07290</td><td>PPR-containing protein</td><td>|||||||-||||||||||||</td></tr>
<tr><td></td><td>Glyma13g30610</td><td>ATP-dependent RNA helicase</td><td>-:||-|:|||||||||||||</td></tr>
<tr><td></td><td>Glyma13g36030</td><td>GH3 auxin-responsive promoter</td><td>||-|||:||::||||||:||</td></tr>
<tr><td></td><td>Glyma16g25410</td><td>PPR-containing protein</td><td>|||||||-||||:|||||||</td></tr>
<tr><td></td><td>Glyma16g27600</td><td>PPR-containing protein</td><td>|||||||-||||||||||||</td></tr>
<tr><td>gma-MIR4413b</td><td>Glyma08g47900</td><td>multifunctional chaperone</td><td>|||||:|||:||-|:|:|||</td></tr>
<tr><td></td><td>Glyma09g07290</td><td>PPR-containing protein</td><td>||||||-|||||||||||||</td></tr>
<tr><td></td><td>Glyma11g07490</td><td>transcriptional repressor activity</td><td>|||||:|||:||-|:|:|||</td></tr>
<tr><td></td><td>Glyma13g30610</td><td>ATP-dependent RNA helicase</td><td>:||-|:|||||||||||||:</td></tr>
<tr><td></td><td>Glyma14g04000</td><td>isomerase activity</td><td>:||||:|||::|||:|||||</td></tr>
<tr><td></td><td>Glyma16g25410</td><td>PPR-containing protein</td><td>||||||-||||:||||||||</td></tr>
<tr><td></td><td>Glyma16g27600</td><td>PPR-containing protein</td><td>||||||-|||||||||||||</td></tr>
<tr><td></td><td>Glyma17g03670</td><td>N-acetyltransferase activity</td><td>-|||:|||:-||||||||||</td></tr>
<tr><td>gma-MIR4415a-3p</td><td>Glyma08g43800</td><td>importin alpha-related</td><td>-:||||||||||||||||:::</td></tr>
<tr><td></td><td>Glyma08g44150</td><td>RNA-binding protein</td><td>|::-|||||||||:|:|||||</td></tr>
<tr><td></td><td>Glyma08g44170</td><td>RNA-binding protein</td><td>|::-|||||||||:|:|||||</td></tr>
<tr><td></td><td>Glyma13g03650</td><td>multicopper oxidases</td><td>-||-|||||||||:|||||||</td></tr>
<tr><td></td><td>Glyma18g08590</td><td>RNA-binding protein</td><td>|::-|||||||||:|:|||||</td></tr>
<tr><td></td><td>Glyma20g12150</td><td>multicopper oxidases</td><td>-||-|||||||||:|||||||</td></tr>
<tr><td></td><td>Glyma20g12230</td><td>multicopper oxidases</td><td>-||-|||||||||:|||||||</td></tr>
</table>

[a] Data from Phytozome version 6.0. [b] Pairing obtained in psRNATarget Server: "|" indicates a Watson-Crick base pairing; ":" is a G:U base pairing, and "-" indicates a mismatch.
