# Supplementary material for: Identification of novel soybean microRNAs involved in abiotic and biotic stresses
Source: BMC Genomics. 2011 Jun 10;12:307. doi: 10.1186/1471-2164-12-307 (PMC3141666; doi:10.1186/1471-2164-12-307)
Supplement: Additional file 3 — The soybean transcript loci which were identified as new-miRNA families target by degradome sequencing. The miRNA target site is indicated in red and underlined while the degradome sequence is highlighted. [file 1471-2164-12-307-S3.PDF]

Additional file 3. Evidence of novel miRNA cleavage sites on mRNA target genes after degradome sequence analysis. The miRNA target site is indicated in red and underlined while the degradome sequence is highlighted.

```
Glyma13g01690 TCCTGATGGTCTACCTGAGACTGATCTGGATGCCACACAGGATATTCCTTCCC
                     -|||||||||||||||-:|
miR-Seq01         19 ACAGAUGGACUCUGACAGG 1

Glyma16g26070 GAGGGGCAGGGCACGAGGTTCCACTTCATAAACCCCG
                   |||||-|-|:|:||||||||
miR-Seq06       20 CGTCCGGAGTTTCAAGGTGA 1

Glyma04g01020 TTCTGCTACTCTTCTCAAGTCATCTCCTGTTCTTGACAAGT
                   ||||-|:|||||||||||-|
miR- Seq07      20 GATGTGGAGAGTTCAGTAAA 1

Glyma19g27280 GTAGCTGCATTTTTAGGTCATTTAATTTCTTGTTATTCAGA
                  ||:||::|:|:|:|||||||
miR- Seq07     20 GATGTGGAGAGTTCAGTAAA 1

Glyma15g13500 TGTGATGCATCAGTTTTGTTGAACAACACTGCCACCATAG
                   ::||-|||||||:|:||||||
miR-Seq11       21 GTGTTGTCAAAATAGCTTGTT 1

Glyma09g02600 GTGACGCATCAGTTTTGTTGAACAACACTGCCACCATAG
                   |:||-|||||||:|:||||||
miR-Seq11       21 GTGTTGTCAAAATAGCTTGTT 1

Glyma08g20670 TTGGTGCATCATCTAGGATTAAGAGTACATGCATCTATGGTG
                   :||||||||||:||:|||||-
miR- Seq12      21 TGTAGTAGATCTTAGTTCTCT 1

Glyma07g01260 TTGGTGCATCATCTAGGATTAAGAGTACATGCATCTATGGTG
                   :||||||||||:||:|||||-
miR- Seq12      21 TGTAGTAGATCTTAGTTCTCT 1

Glyma17g20860 TTTCAATTCCAGATGTCTGATTATAAAATTGTGGTGACTTCA
                   ||||||||||||-||:|||||
miR-Seq16       21 TAAGGTCTACAGTCTGATATT 1

Glyma05g09440 TTTCAATTCCAGATGTCTGATTATAAAATTGTGGTGACTTCT
                   ||||||||||||-||:|||||
miR-Seq16       21 TAAGGTCTACAGTCTGATATT 1
```
